# Convergent Synthesis of *N,S-bis* Glycosylquinolin-2-ones via a Pd-G3-XantPhos Precatalyst Catalysis

**DOI:** 10.3390/molecules23030519

**Published:** 2018-02-26

**Authors:** Wafa Redjdal, Nada Ibrahim, Belkacem Benmerad, Mouad Alami, Samir Messaoudi

**Affiliations:** 1 Laboratoire de Physico-Chimie des Matériaux et Catalyse, Faculté des Sciences Exactes, Université de Bejaia, 0600 Bejaia, Algeria; wafasmanalyse@gmail.com (W.R.); belkacem.benmerad@univ-bejaia.dz (B.B.); 2 BioCIS, Université Paris-Sud, CNRS, University Paris-Saclay, 92296 Châtenay-Malabry, France; nada.makky@u-psud.fr

**Keywords:** thiosugars, Buchwald-Hartwig-Migita coupling, *N*-glycosylquinolin-2-ones, *bis*-*N*,*S*-glycosyl quinolin-2-ones

## Abstract

Buchwald-Hartwig-Migita cross-coupling of 1-thiosugars with α- or β-3-iodo-*N*-glycosylquinolin-2-ones has been accomplished under mild and operationally simple reaction conditions through the use of a Pd-G3 XantPhos palladacycle precatalyst. This new methodology has been successfully applied to a variety of α- or β-mono-, di-, and poly-thiosugar derivatives to efficiently synthesize a series of α- or β-*N*,*S*-*bis*-glycosyl quinolin-2-ones, which are difficult to synthesize by classical methods.

## 1. Introduction

**Figure 1:**
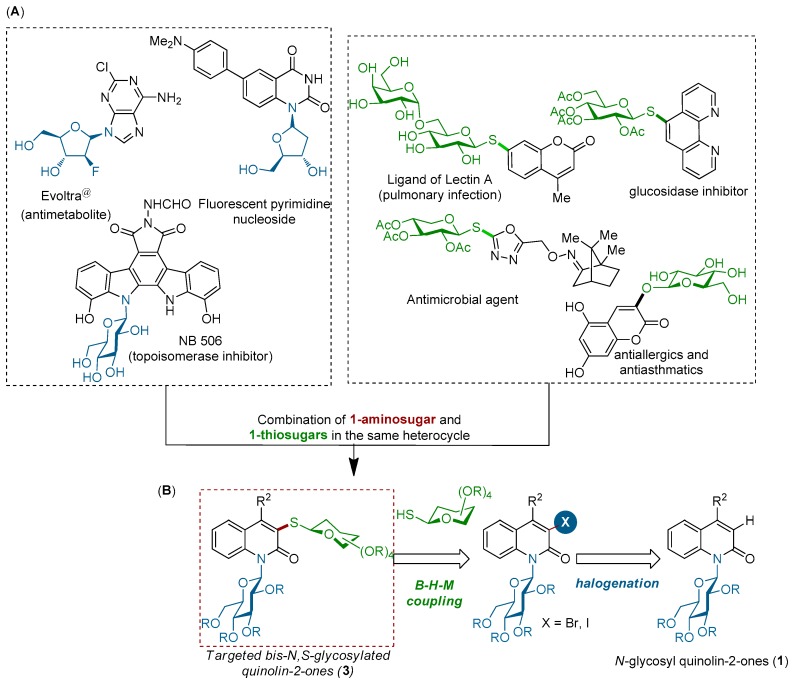
(**A**) Heteroaryl glycoside-based bioactive molecules; (**B**) General structure of the targeted -*N,S*-*bis*-glycosylated quinolinones (**3**).

Heteroaryl-glycosides are of high importance in medicinal chemistry and commonly found in many compounds of enormous practical importance, ranging from natural compounds to pharmaceutical agents [1,2,3,4,5,6,7,8,9,10,11,12,13,14,15,16,17,18,19] (Figure 1). While these derivatives clearly hold great potential in medicinal chemistry, relatively little attention has been devoted to the synthesis of heteroaryl-*bis*-glycosides bearing two different sugar moieties, such as thiosugars and aminosugars, in the same heterocycle. One of the most important subfamilies of heteroaryl-*N*-glycosides is *N*-glycosyl quinolin-2-ones, in which a glycosyl unit is attached to a quinolin-2-one core. Quinolin-2-(1*H*)-ones are present in many biologically active compounds and pharmaceutical agents [20,21,22,23,24,25,26,27]. Thus, the attachment of *S*- and *N*-glycosyl units to a quinolin-2-one nucleus can cause several changes in their features, including their chemical, physical, biochemical, and biological properties. Thus, developing a simple method for the synthesis of *bis N*,*S*-glycosyl quinolin-2-ones would be of great interest for the preparation of large libraries of new potentially active compounds. Recently, our group reported an efficient protocol for the synthesis of *N*-glycosyl quinolin-2-ones (Figure 1) via a palladium-catalyzed intramolecular *N*-arylation of various substituted (2-iodophenyl)-acrylamidosugars [28,29]. As part of our continued efforts to functionalize sugars under transition-metal catalysis to access complex glycosides [30,31,32,33,34,35,36], we envisioned whether *N*-glycosyl quinolin-2-ones of type (**1**) could be utilized as a building block in the synthesis of 3-thioglycosyl *N*^1^-glycosyl quinolin-2-ones (**3**) through C3-halogenation followed by the Pd(0)-catalyzed Buchwald-Hartwig-Migita (B-H-M) coupling reaction with various thiosugars (Figure 1). This modular strategy is conceptually attractive in terms of diversifying the *N*-glycosyl quinolinone frameworks with the aim of identifying novel scaffolds of biological interest. Herein, we report our success in the development of such a strategy.

## 2. Results and Discussion

### 2.1. Synthesis of Starting Materials

**Scheme 1:**
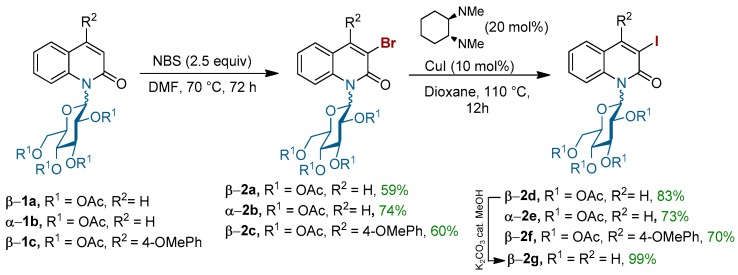
Halogenation of *N*-(β-glucopyranosyl)quinolin-2-ones.

To establish the appropriate conditions for the coupling of 3-halo-*N*-glycosyl quinolin-2-ones with various thiosugars, we initially started our chemistry by the synthesis of the appropriate α- or β-3-halo-*N*-glycosyl quinolin-2-ones **2a**–**g** (Scheme 1). The compounds **2a**–**c** were prepared by the electrophilic regioselective aromatic bromination of *N*-glycosyl quinolin-2-one **1a**–**c** using *N*-bromosuccinimide in anhydrous dimethylformamide (DMF). Under these conditions, 3-bromo-*N*-glucopyranosylquinolin-2-ones **2a**–**c** were isolated in good yields (Scheme 1). To compare the reactivity of brominated quinolinones **2a**–**c** with its analogue, in which the bromine atom is replaced by the iodine one, derivatives **2d**–**f** were synthesized from **2a**–**c** through a halogen exchange by a Cu-catalyzed Finkelstein reaction [37]. Finally, compound β-**2g** bearing an unprotected sugar was also prepared in order to study the influence of a free hydroxyls group on the outcome of the coupling.

### 2.2. Optimization of the Reaction Conditions on the Model Study

**Scheme 2:**
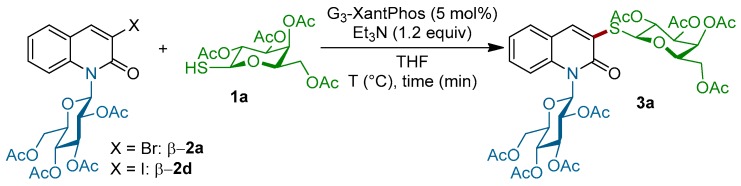
Coupling of the quinolones β-**2a** and β-**2d** with *tetra*-*O*-acetylated 1-thio-β-d-galactopyranose **1a** under various reaction conditions.

With these starting materials in hand, we turned our attention to explore the feasibility of the coupling of the quinolones β-**2a** and β-**2d** with *tetra*-*O*-acetylated 1-thio-β-d-galactopyranose **1a** under various reaction conditions (Scheme 2). When β-**2a** and **1a** were mixed under our previously reported conditions [38] (G3-XantPhos (5 mol %), Et_3_N (1.5 equiv.) in tetrahydrofuran (THF) at room temperature), only the starting material was recovered unchanged; however, when the reaction mixture was heated at 60 °C, product **3a** was detected by NMR of the crude reaction mixture and the conversion rate was calculated to be around 35% (Table 1, entry 2). The conversion rate has never exceeded 50%, even when the amount of the thiogalactose **1a** was increased until 2.5 equiv. and the reaction temperature was at 100 °C, probably due to the fact that the formation of disulfide dimer was faster than the coupling of product **3a**. In the next set of experiences, we decided to use the iodinated quinolinone β-**2d** instead of β-**2a**. Delightfully, the coupling of β-**2d** with **1a** in the presence of Pd-G3-XantPhos (5 mol %), with Et_3_N (1.2 equiv.) as the base in THF at room temperature, led to *N*-β-glycosyl *S*-β-galactosyl quinolin-2-one **3a** (*J*_1,2_ = 9.9 Hz) in 70% yield (entry 3, Table 1). Decreasing the amount of thiogalactose **1a** into 1.5 equiv. led to a lower conversion rate (40%, entry 4), indicating that the thiosugar concentration plays a critical role in the outcome of the reaction. It should be noted that the palladium catalyst is necessary to achieve this transformation, since no reaction occurs when the coupling is conducted in the absence of the Pd-G3-precatalyst.

### 2.3. Scope and Limitation of the Cross-Coupling

**Scheme 3:**
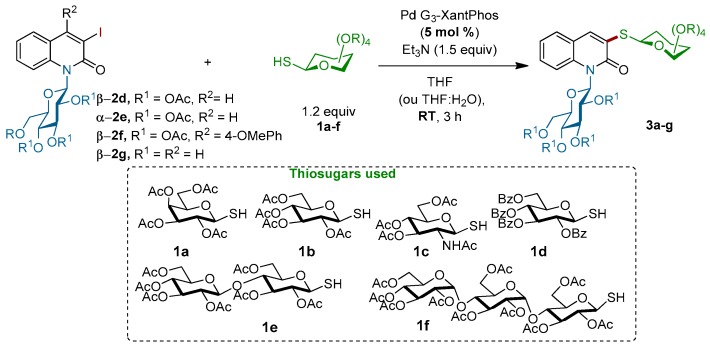
Coupling of the quinolones **2d**–**g** with thiosugars **1a**–**f** under the optimized conditions.

**Figure 2:**
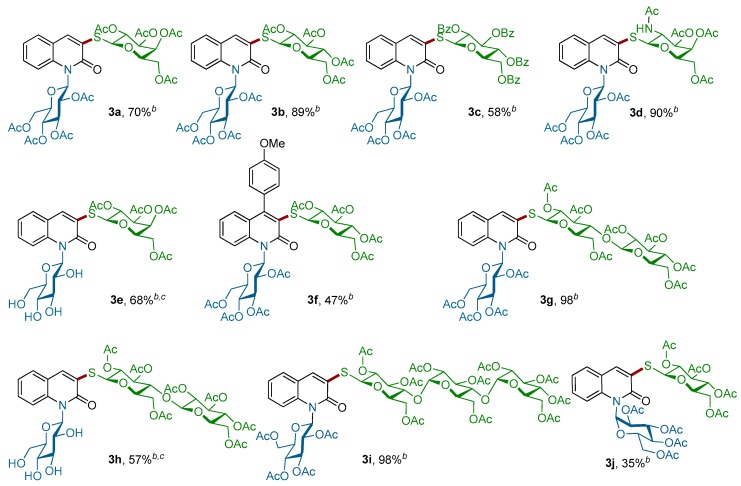
Scope of thiosugars **1a**–**f** for the Pd-catalyzed coupling with *N*-glucosylquinolinones **2d**–**g** *^a^*. *^a^* Conditions: Reactions of **1** (2.5 equiv.) with **2** (1.0 equiv.) were performed in a resealable tube by using Pd-G3-XantPhos (5 mol %) and Et_3_N (1.5 equiv.) in 1,4-dioxane (0.1 M) at room temperature for 3 h; *^b^* Yield of isolated; *^c^* THF:H_2_O (8:2) was used as a solvent.

Motivated by these results, we next explored the scope of the coupling reaction of structurally diverse mono-, di-, and tri-thiosugar derivatives **1a**–**f** with various α- or β-*N*-glucosylquinolinones **2d**–**g** (Scheme 3). Gratifyingly, all of the couplings proceeded in good yields as well as with a retention of the anomeric configuration. The nature of the *N*-β-glucosylquinolinone partner does not interfere with the outcome of the reaction, since both *O*-acetylated β-glucosylquinolinone β-**2d** and unprotected β-glucosylquinolinone β-**2g** were successfully coupled. Regarding the thio-nucleophilic partners, this coupling reaction tolerates a large variety of thiosugars **1a**–**f**: *O*-acetylated 1-thio-β-d-galactopyranose **1a**, *O*-acetylated 1-thio-β-d-glycopyranose **1b**, *O*-acetylated *N*-Ac-1-thio-β-d-glucopyranose **1c**, and *O*-benzoylated 1-thio-β-d-glucopyranose **1d** were coupled with both glucosylquinolinones β-**2d** and β-**2g** to give the β-*N*,*S*-*bis*-glycosyl quinolin-2-ones **3a**–**f** without any loss of reactivity, except for the *O*-acetylated β-glucosylquinolinone β-**2f** due to the steric effects (Figure 2).

**Scheme 4:**
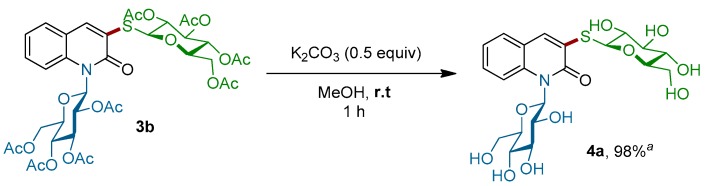
Deprotection of β-*N*,*S*-*bis* glycosyl quinolin-2-one **3b**. ***^a^*** isolated yield.

Importantly, this procedure is not limited to only β-glucosyl quinolin-2-ones, but it also worked successfully with 1-*N*-glucosylquinolin-2-one α-**2e**, which had an anomeric α-configuration. In this case, the corresponding α-*N*,*S*-*bis*-glycosyl quinolin-2-one **3j** was obtained with a slightly lower yield of 35%. Finally, the efficiency of this C–S bond-forming reaction was well-demonstrated by the coupling of more complex di- and trisaccharide derivatives. Thus, 1-thio-β-d-cellobiose **1e** as well as 1-thio-β-d-maltotriose **1f** were readily reacted with β-**2d** and β-**2g** to give the corresponding thioglycosides **3g**–**i** in 97%, 57% and 98% yields, respectively. More importantly, the stereochemistry of the β-1,4′-*O*-glycosidic bond in the di-saccharides **3g**,**h** and the α-1,4′ in β-tri-saccharide **3i** remained intact. It is worth noting that all our attempts to react an unprotected thiogalactose with β-**2d** or β-**2g** under our optimized conditions failed. Alternatively, in order to produce completely unprotected β-*N*,*S*-*bis*-glycosyl quinolin-2-ones and show that their purification and characterization may be achieved easily, the deprotection of representative β-*N*,*S*-*bis*-glycosyl quinolin-2-one was performed (Scheme 4). Thus, acetyl protecting groups of **3b** could be removed through the Zemplen reaction [39,40,41] by using a catalytic amount of potassium carbonate as the base in methanol. Under these conditions, unprotected β-*N*,*S*-*bis*-glycosyl quinolin-2-one **4a** was isolated in a quantitative yield.

## 3. Materials and Methods

### 3.1. General Experimental Methods

The compounds were all identified by the usual physical methods, that is, ^1^H-NMR, ^13^C-NMR, IR, and MS (ESI). ^1^H- and ^13^C-NMR spectra were measured in CDCl3 or DMSO-*d*_6_, Acetone-*d*_6_ or MeOH-*d*_4_ with NMR 300 and 400. ^1^H chemical shifts are reported in ppm from an internal standard trimethylsilane (TMS). The following abbreviations are used: m (multiplet), s (singlet), bs (broad singlet), d (doublet), t (triplet), dd (doublet of doublet), td (triplet of doublet), q (quadruplet), qui (quintuplet), sex (sextuplet). ^13^C chemical shifts are reported in ppm from the central peak of deuterochloroform (77.14), acetone d6 (29.84 and 206.26), MeOH (49.00), and DMSO (39.52). High resolution mass spectra (HR-MS) were recorded on a Micromass spectrometer, using ESI. IR spectra were measured and are reported in wave numbers (cm^−1^).

### 3.2. Typical Procedure A for the Synthesis of β or α 3-iodo N-glucosylquinolinones ***2a**–**c***

A 50-mL round tube flash was charged with **1a**–**c** (1 equiv.), freshly crystallized *N*-bromosuccinimide (NBS) (2.5 equiv.). Under an argon atmosphere, anhydrous DMF was added. The mixture was heated to 70 °C and stirred until reaction completeness (72 h) ascertained by thin layer chromatography (TLC). The crude was diluted with EtOAc and extracted with saturated NH_4_Cl (50 mL × 3). The organic layer was washed with water, dried by MgSO_4_, and concentrated under vacuum. The residue was purified by silica gel column chromatography.

### 3.3. Typical Procedure B for the Synthesis of β or α 3-iodo N-glucosylquinolinones ***2d**–**g***

A reactor tube was charged with CuI (10 mol %), *trans*-*N*,*N*′-dimethylcyclohexane-1,2-diamine (20 mol %), NaI (2 equiv.), and β- or α-3-bromo *N*-glucosylquinolinones **2a**–**c** (1 equiv., 0.721 mmol) followed by the addition of 1,4-dioxane (12 mL). The reaction under argon atmosphere was then stirred at 110 °C in an oil bath overnight. The mixture was cooled to room temperature. The crude product was purified by silica gel flash chromatography.

### 3.4. Typical Procedure C for Pd-Catalyzed Coupling of Thiosugars (***1a**–**f***) with β- or α-3-iodo N-glucosylquinolin-2-ones (***2d**–**g***)

A resealable and dry tube (5 mL) was charged with XantPhos Pd-G3 (5.0 mol %), thiosugar **1a**–**f** (2.5 equiv.), and 3-iodo *N*-glucosylquinolin-2-ones **2d**–**g** (0.083 mmol, 1.0 equiv.). The tube was capped with a rubber septum, evacuated, and backfilled with argon. Then, THF (1 mL) or THF/H_2_O (0.8 mL THF, 0.2 mL H_2_O) for unprotected compounds and Et_3_N (1.2 equiv.) were added. The tube was sealed and the mixture was stirred at room temperature for 2–3 h. After evaporation of the THF or THF/H_2_O, the residue was then purified by flash chromatography over silica gel. This first purification was followed by HPLC preparative for products **3e**, **3g**, **3h**, **3i** and **3j**. The column used was XSELECT 4.6 × 150 mm–5 μm.

### 3.5. Typical Procedure D for the Synthesis of Unprotected 3-iodo Glucosylquinolinone β-***2g***

A mixture of 3-iodo β-*N*-glucosylquinolinones (100 mg, 1.0 equiv.) and K_2_CO_3_ (12 mg, 0.5 equiv.) in methanol (3 mL) was placed in a small balloon and the mixture was stirred under argon at room temperature for 30 min to 1 h. The crude mixture was then filtered through celite, washed with 10 mL of methanol, and filtered for only 1 min. The filtrate was concentrated under reduced pressure at 25 °C for 1–2 h.

*(2R,3R,4S,5R,6R)-2-(acetoxymethyl)-6-(3-bromo-2-oxoquinolin-1(2H)-yl)tetrahydro-2H-pyran-3,4,5-triyl triacetate* **2a**: Following procedure A, A 50-mL round tube flash was charged with **1a** *N*-(2,3,4,6-tetra-*O*-acetyl-1-deoxy-α-d-glucopyranosyl)-quinolin-2-one (500 mg, 1 equiv.) and freshly crystallized NBS (469 mg, 2.5 equiv.). Under an argon atmosphere, 30 mL of anhydrous DMF was added. The mixture was heated to 70 °C and stirred until reaction completeness (72 h), ascertained by TLC. The crude was diluted with EtOAc and extracted with saturated NH_4_Cl (50 mL × 3). The organic layer was washed with water, dried by MgSO_4_, and concentrated under vacuum. The residue was purified by silica gel column chromatography (cyclohexane/EtOAc 6/4) to afford the desired product **2a** as white powder (345 mg, 59%); m.p. = 213–215 °C. ${\left[\alpha \right]}_{D}^{20}$ + 103.0 (c, 1.0 in CHCl_3_). ^1^H-NMR (300 MHz, CDCl_3_) δ 8.08 (s, 1H), 7.95 (d, *J* = 8.8 Hz, 1H), 7.58 (dd, *J* = 7.3 Hz, 8.6 Hz, 1H), 7.48 (d, *J* = 7.4 Hz, 1H), 7.29 (t, *J* = 7.9 Hz, 1H), 6.89 (d, *J* = 10.0 Hz, 1H), 5.89 (t, *J* = 9.3 Hz, 1H), 5.46 (t, *J* = 9.3 Hz, 1H), 5.36 (t, *J* = 9.8 Hz, 1H), 4.30 (dd, *J* = 12.4, 4.4 Hz, 1H), 4.23 (dd, *J* = 12.5, 2.0 Hz, 1H), 4.08–4.00 (m, 1H), 2.09 (s, 6H), 2.00 (s, 3H), 1.81 (s, 3H); ^13^C-NMR (75 MHz, CDCl3) δ 170.5 (C), 169.9 (C), 169.8 (C), 169.3 (C), 158.8 (C), 142.8 (CH), 137.1 (C), 130.8 (CH), 128.9 (CH), 123.9 (CH), 121.1 (C), 116.9 (CH), 82.4 (CH), 75.3 (CH), 73.8 (CH), 68.0 (CH), 67.9 (CH), 61.8 (CH2), 20.9 (CH3), 20.7 (CH3), 20.7 (CH3), 20.3 (CH3). FT-IR (neat, cm^−1^) 1757, 1652, 1366, 1229, 1032, 905, 837. HRMS (ESI, *m*/*z*) calcd. for C_23_H_24_NO_10_BrNa [M + Na]^+^: 576.0481 found 576.0485.

*(2S,3S,4R,5S,6R)-2-(acetoxymethyl)-6-(3-bromo-2-oxoquinolin-1(2H)-yl)tetrahydro-2H-pyran-3,4,5-triyl triacetate* **2b**: Following procedure A, a 30-mL round tube flash was charged with **1b** *N*-(2,3,4,6-tetra-*O*-acetyl-1-deoxy-α-d-glucopyranosyl)-quinolin-2-one (200 mg, 1 equiv.) and freshly crystallized NBS (188 mg, 2.5 equiv.). Under an argon atmosphere, 12 mL of anhydrous DMF was added. The mixture was heated to 70 °C and stirred until reaction completeness (72 h), ascertained by TLC. The crude was diluted with EtOAc and extracted with saturated NH_4_Cl (50 mL × 3). The organic layer was washed with water, dried by MgSO_4_, and concentrated under vacuum. The residue was purified by silica gel column chromatography (heptane/EtOAc 6/4) to afford the desired product **2b** as white powder (173 mg, 74%); ^1^H-NMR (300 MHz, CDCl_3_) δ 8.11 (s, 1H), 7.76 (d, *J* = 8.6 Hz, 1H), 7.54 (dd, *J* = 8.6 Hz, 7.4, 1H), 7.47 (d, *J* = 7.6 Hz, 1H), 7.26 (t, *J* = 7.4 Hz, 1H), 5.37 (t, *J* = 6.4 Hz, 1H), 5.24 (t, *J* = 7.8 Hz, 1H), 4.71–4.62 (m, 1H), 4.39 (dd, *J* = 12.4, 4.8 Hz, 1H), 4.16 (dd, *J* = 12.4, 2.7 Hz, 1H), 2.10 (s, 3H), 2.08 (s, 3H), 2.05 (s, 3H), 1.73 (s, 3H); ^13^C-NMR (75 MHz, CDCl_3_) δ 170.7 (C), 169.9 (C), 169.8 (C), 169.8 (C), 159.5 (C), 142.4 (CH), 139.4 (C), 130.7 (CH), 128.1 (CH), 123.7 (CH), 121.3 (C), 117.1 (C), 116.6 (CH), 80.8 (CH), 73.4 (CH), 72.5 (CH), 70.2 (CH), 68.0 (CH), 61.7 (CH_2_), 21.0 (CH_3_), 20.9 (CH_3_), 20.9 (CH_3_), 20.4 (CH_3_).

*(2R,3R,4S,5R,6R)-2-(acetoxymethyl)-6-(3-bromo-4-(4-methoxyphenyl)-2-oxoquinolin-1(2H)-yl)tetrahydro-2H-pyran-3,4,5-triyl triacetate* **2c**: Following procedure A, a 10-mL round tube flash was charged with **1c** (50 mg, 1 equiv.) and freshly crystallized NBS (39 mg, 2.5 equiv.). Under an argon atmosphere, 3 mL of anhydrous DMF was added. The mixture was heated to 70 °C and stirred until reaction completeness (4 h), ascertained by TLC. The crude was diluted with EtOAc and extracted with saturated NH_4_Cl (50 mL × 3). The organic layer was washed with water, dried by MgSO_4_, and concentrated under vacuum. The residue was purified by silica gel column chromatography (cyclohexane/EtOAc 5/5) to afford the desired product **2c** as pale yellow solid (34 mg, 60%); m.p.: 105–107 °C; R*f* = 0.48 (Ethyl/Cyclohexane: 5/5); ${\left[\alpha \right]}_{D}^{17}$ + 35.55 (c, 1.0 in CHCl_3_); IR (neat): 2963, 1756, 1650, 1604, 1554, 1511, 1455, 1366, 1260, 1248, 1033,1013,802, 763 cm^−1^; ^1^H-NMR (300 MHz, CDCl_3_) δ 8.00 (d, *J* = 8.5 Hz, 1H), 7.59–7.53 (m, 1H), 7.22–7.13 (m, 4H), 7.02 (dd, *J* = 17.8, 9.3 Hz, 3H), 5.97 (t, *J* = 9.3 Hz, 1H), 5.44 (dt, *J* = 30.0, 9.5 Hz, 2H), 4.33–4.24 (m, 2H), 4.17–4.09 (m, 1H), 3.90 (s, 3H), 2.11 (s, 6H), 2.02 (s, 4H), 1.85 (s, 2H); ^13^C-NMR (75 MHz, CDCl_3_) δ 170.56, (C) 169.95 (C), 169.77 (C), 169.65 (C), 169.28 (C), 160.00 (C), 158.88 (C), 152.51 (C), 136.55 (C), 130.55 (CH), 130.08 (CH), 129.90 (CH), 129.42 (C), 129.25 (CH), 123.56 (CH), 122.35 (C), 116.76 (CH), 114.23 (2CH), 82.55 (CH), 75.33 (CH), 73.96 (CH), 68.05 (CH), 61.85 (CH2), 55.49 (CH) 20.86 (CH_3_), 20.75 (CH_3_), 20.73 (2CH_3_), 20.37 (CH_3_); HR-MS (ESI positive, *m*/*z*): found 682.0894 ([M + Na]^+^), calc. for C_30_H_30_NO_11_NaBr (M + Na): 682.0900.

*(2R,3R,4S,5R,6R)-2-(acetoxymethyl)-6-(3-iodo-2-oxoquinolin-1(2H)-yl)tetrahydro-2H-pyran-3,4,5-triyl triacetate* **2d**: Following procedure B, a solution of β-3-bromo *N*-glycosylquinolinone **2a** (0.722 mmol, 400 mg), NaI (1.443 mmol, 217 mg), trans-*N*,*N*′-dimethylcyclohexane-1,2-diamine (21 mg, 0,14 mmol), and CuI (14 mg, 0.072 mmol) was stirred at 110 °C overnight. The crude product was purified by silica gel flash chromatography (Cyclohexane/EtOAc 5/5), and the product **2d** was isolated as a pale yellow solid (362 mg, 83%); m.p.: 222.7–223.8 °C; R*f* = 0.56 (Cyclohexane/EtOAc: 5/5); ${\left[\alpha \right]}_{D}^{17}$ + 96.29 (c, 1.0 in CHCl_3_); IR (neat): 1746, 1648, 1596, 1365, 1217, 1077, 914, 751 cm^−1^; ^1^H-NMR (300 MHz, CDCl_3_) δ 8.35 (s, 1H), 7.95 (d, *J* = 8.6 Hz, 1H), 7.58 (t, *J* = 7.9 Hz, 1H), 7.45 (d, *J* = 7.7 Hz, 1H), 7.31–7.24 (m, 1H), 6.89 (d, *J* = 9.8 Hz, 1H), 5.88 (t, *J* = 9.3 Hz, 1H), 5.49–5.29 (m, 2H), 4.33–4.19 (m, 2H), 4.07–3.97 (m, 1H), 2.10 (s, 6H), 2.00 (s, 3H), 1.80 (s, 3H); ^13^C-NMR (75 MHz, CDCl_3_) δ 170.55, 169.90, 169.76, 169.24, 159.22, 150.33, 137.80, 131.02, 128.61, 123.69, 122.32, 116.94, 92.17, 82.92, 75.30, 73.77, 68.03, 67.94, 61.82, 20.85, 20.74, 20.70, 20.27; HR-MS (ESI positive, *m*/*z*): found 624.0339 ([M + Na]^+^), calc. for C_23_H_24_NO_10_NaI (M + Na): 624.0343.

*(2S,3S,4R,5S,6R)-2-(acetoxymethyl)-6-(3-iodo-2-oxoquinolin-1(2H)-yl)tetrahydro-2H-pyran-3,4,5-triyl triacetate* **2e**: Following procedure B, α-3-bromo *N*-glycosylquinolinone **2b** (0.288 mmol, 160 mg), NaI (0.577 mmol, 87 mg), trans-*N*,*N*′-dimethylcyclohexane-1,2-diamine (8.2 mg, 0,057 mmol), and CuI (5.5 mg, 0.028 mmol) were stirred at 110 °C overnight. The crude product was purified by silica gel flash chromatography (EtOAc/heptan: 5/5), and the product **2e** was isolated as a pale yellow solid (124 mg, 73%): m.p.: 111.4–123.4 °C; R*f* = 0.26 (EtOAc/heptan: 5/5); ${\left[\alpha \right]}_{D}^{16}$ + 370 (c, 1.0 in CHCl_3_); IR (neat): 1739, 1641, 1596, 1367, 1260, 1205, 1031, 817, 798 cm^−1^; ^1^H-NMR (300 MHz, CDCl_3_) δ 8.37 (s, 1H), 7.77 (d, *J* = 8.7 Hz, 1H), 7.53 (t, *J* = 7.9 Hz, 1H), 7.44 (d, *J* = 7.8 Hz, 1H), 7.23 (d, *J* = 7.6 Hz, 1H), 6.70 (d, *J* = 6.2 Hz, 1H), 6.01 (t, *J* = 6.7 Hz, 1H), 5.36 (t, *J* = 6.3 Hz, 1H), 5.23 (t, *J* = 7.7 Hz, 1H), 4.70–4.63 (m, 1H), 4.39 (dd, *J* = 12.4, 4.9 Hz, 1H), 4.17 (dd, *J* = 12.4, 3.0 Hz, 1H), 2.13–2.04 (m, 9H), 1.73 (s, 3H); ^13^C-NMR (75 MHz, CDCl_3_) δ 170.73 (C), 169.93 (C), 169.76 (2C), 160.03 (C), 149.95 (CH), 140.12 (C), 130.86 (CH), 127.84 (CH), 123.48 (CH), 122.48 (C), 116.70(CH), 93.58 (C), 80.99 (CH), 73.35 (CH), 72.45 (CH), 70.17 (CH), 68.06 (CH), 61.67 (CH_2_), 21.06 (CH_3_), 20.94 (CH_3_), 20.89 (CH_3_), 20.46 (CH_3_); HR-MS (ESI positive, *m*/*z*): found 624.0344 ([M + Na]^+^), calc. for C_23_H_24_NO_10_NaI (M + Na): 624.0343.

*(2R,3R,4S,5R,6R)-2-(acetoxymethyl)-6-(3-iodo-4-(4-methoxyphenyl)-2-oxoquinolin-1(2H)-yl)tetrahydro-2H-pyran-3,4,5-triyl triacetate* **2f**: Following procedure B, *N*-glycosylquinolinone β-**2c** (0.136 mmol, 90 mg), NaI (0.272 mmol, 41 mg), trans-*N*,*N*′-dimethylcyclohexane-1,2-diamine (4 mg, 0,027 mmol), and CuI (3 mg, 0.0135 mmol) were stirred at 110 °C overnight. The crude product was purified by silica gel flash chromatography (Cyclohexane/EtOAc 6/4), and the product **2f** was isolated as a yellow solid (67 mg, yield 70%); m.p.: 106.6–121.8 °C; R*f* = 0.48 (Cyclohexane/EtOAc 6/4); ${\left[\alpha \right]}_{D}^{16}$ + 22.35 (c, 1.0 in CHCl_3_); IR (neat): 1756, 1652, 1604, 1511, 1367, 1249, 1217, 1113, 1097, 1035, 765 cm^−1^; ^1^H-NMR (300 MHz, Acetone) δ 8.34 (d, *J* = 8.6 Hz, 1H), 7.70–7.63 (m, 1H), 7.26–7.06 (m, 5H), 7.02 (d, *J* = 9.9 Hz, 1H), 5.98 (t, *J* = 10.6 Hz, 1H), 5.59 (t, *J* = 9.5, 1H), 5.47 (t, *J* = 9.2 Hz, 1H), 4.37–4,28 (m, 3H), 4.06 (d, *J* = 7.1 Hz, 1H), 3.92 (s, 3H), 2.11–2.01 (m, 9H), 1.97 (s, 3H); ^13^C-NMR (75 MHz, Acetone) δ 170.84, 170.25, 169.48, 167.30, 161.09, 154.96, 137.19, 131.79, 130.85, 130.54, 130.05, 124.11, 117.14, 115.16 (3), 83.93, 76.03, 74.54, 69.00 (2), 62.72, 55.89, 20.84 (2), 20.72, 20.30. HR-MS (ESI positive, *m*/*z*): found 730.0739 ([M + Na]^+^), calc. for C_30_H_30_NO_11_NaI (M + Na): 730.0761.

*3-iodo-1-((2R,3R,4S,5S,6R)-3,4,5-trihydroxy-6-(hydroxymethyl)tetrahydro-2H-pyran-2-yl)quinolin-2(1H)-one* **2g**: Following procedure D, a mixture of β-**2d** (100 mg, 1.0 equiv.) and K_2_CO_3_ (12 mg, 0.5 equiv.) in methanol (3 mL) was stirred under argon at room temperature for 30 min to 1 h. The crude mixture was then filtered through celite, washed with 10 mL of methanol, and filtered for only 1 min. The filtrate was concentrated under reduced pressure at 25 °C for 1–2 h. The product **2g** was isolated as a pale brown solid (100 mg, yield 99%); m.p.: 155.2–171.8 °C; R*f* = 0.4 (Ethyl/MeOH: 9/1); ${\left[\alpha \right]}_{D}^{18}$ + 45 (c, 1.0 in MeOH); IR (neat): 3360, 1629, 1589, 1451, 1314, 1277, 1082, 766, 749 cm^−1^; ^1^H-NMR (300 MHz, MeOD-*d*_4_) δ 8.59 (s, 1H), 8.10 (d, *J* = 8.9 Hz, 1H), 7.58 (t, *J* = 9.7 Hz, 2H), 7.27 (t, *J* = 7.4 Hz, 1H), 6.57 (d, *J* = 9.8 Hz, 1H), 4.27 (t, *J* = 9.0 Hz, 1H), 3.94 (d, *J* = 11.8 Hz, 1H), 3.81 (dd, *J* = 12.5, 4.6 Hz, 1H), 3.62–3.53 (m, 3H); ^3^C-NMR (75 MHz, MeOD) δ 161.39, 151.53, 132.47, 131.55, 129.90, 129.46, 124.33, 124.02, 119.17, 86.94, 81.86, 79.54, 71.30, 70.75, 62.62; HR-MS (ESI positive, *m*/*z*): found 455.9928 ([M + Na]^+^), calc. for C_15_H_16_NO_6_NaI (M + Na): 455.9920.

*(2R,3S,4S,5R,6S)-2-(acetoxymethyl)-6-((2-oxo-1-((2R,3R,5R,6R)-3,4,5-triacetoxy-6-(acetoxymethyl)tetrahydro-2H-pyran-2-yl)-1,2-dihydroquinolin-3-yl)thio)tetrahydro-2H-pyran-3,4,5-triyl triacetate* **3a**: Following procedure C, 3-iodo *N*-β-glycosylquinolinone β-**2d** (0.0831 mmol, 50 mg) and β-thiogalactose **1a** (0.2077 mmol, 76 mg) were stirred at room temperature for 3 h. The crude product was purified by silica gel flash chromatography (Cyclohexane/EtOAc 4/6). The product **3a** was isolated as pale yellow solid (46 mg, yield 70%); m.p.: 222.7–223.8 °C; R*f* = 0.4 (Cyclohexan/EtOAc: 4/6); ${\left[\alpha \right]}_{D}^{17}$ + 16.21 (c, 1.0 in CHCl_3_); IR (neat): 1745, 1648, 1595, 1367, 1208, 1034, 794, 733,702 cm^−1^; ^1^H-NMR (300 MHz, Acetone) δ 8.28 (d, *J* = 8.7 Hz, 1H), 7.98 (s, 1H), 7.69–7.58 (m, 2H), 7.33 (t, *J* = 7.5 Hz, 1H), 6.94 (d, *J* = 9.7 Hz, 1H), 5.94 (t, *J* = 9.3 Hz, 1H), 5.60–5.26 (m, 6H), 4.50 (t, *J* = 6.3 Hz, 1H), 4.41–4.26 (m, 3H), 4.18 (d, *J* = 6.3 Hz, 2H), 2.19 (s, 3H), 2.10–1.92 (m, 21H); ^13^C-NMR (75 MHz, Acetone-*d*_6_) δ 169.87(C), 169.75 (C), 169.67 (C), 169.25 (C), 169.21 (C), 169.15 (C), 169.04 (C), 168.38 (C), 159.72 (C), 135.91 (C), 135.60 (CH), 129.62 (CH), 128.41 (CH), 127.91 (C), 123.16 (CH), 121.09 (C), 117.49 (CH), 81.87 (CH), 81.25 (CH), 74.84 (CH), 74.51 (CH), 73.38 (CH), 71.56 (CH), 67.88 (CH), 67.81 (CH), 67.74 (CH), 66.74 (CH), 61.96 (CH_2_), 61.55 (CH_2_), 19.74 (6CH_3_), 19.61 (CH_3_), 19.20 (CH_3_). HR-MS (ESI positive, *m*/*z*): found 860.2037 ([M + Na]^+^), calc. for C_37_H_43_NO_19_NaS (M + Na): 860.2048.

*(2R,3R,4S,5R,6S)-2-(acetoxymethyl)-6-((2-oxo-1-((2R,3R,5R,6R)-3,4,5-triacetoxy-6-(acetoxymethyl)tetrahydro-2H-pyran-2-yl)-1,2-dihydroquinolin-3-yl)thio)tetrahydro-2H-pyran-3,4,5-triyl triacetate* **3b**: Following procedure C, 3-iodo *N*-β-glycosylquinolinone β-**2d** (0.0831 mmol, 50 mg) and β-thioglucose **1b** (0.2077 mmol, 76 mg) were stirred at room temperature for 3 h. The crude product was purified by silica gel flash chromatography (diethylether/Pentan: 9/1), and the product **3b** was isolated as a pale yellow solid (62 mg, yield 89%); m.p.: 221.8–223 °C; R*f* = 0.33 (diethylether/Pentan: 9/1); ${\left[\alpha \right]}_{D}^{17}$ + 23.07 (c, 1.0 in CHCl_3_); IR (neat): 1756, 1649, 1595, 1366, 1206, 1032, 914, 798,764 cm^−1^; ^1^H-NMR (300 MHz, Acetone) δ 8.27 (d, *J* = 8.8 Hz, 1H), 7.92 (s, 1H), 7.70–7.58 (m, 2H), 7.33 (t, *J* = 7.5 Hz, 1H), 6.94 (d, *J* = 9.8 Hz, 1H), 5.94 (t, *J* = 9.4 Hz, 1H), 5.58 (dd, *J* = 18.7, 9.2 Hz, 1H), 5.51–5.38 (m, 3H), 5.21–5.09 (m, 2H), 4.40–4.16 (m, 6H), 2.08–1.96 (m, 24H); ^13^C-NMR (75 MHz, Acetone) δ 169.75 (C), 169.37 (C), 169.20 (C), 169.14 (2C), 168.97 (C), 168.91 (C), 168.38 (C), 159.68 (C), 135.86 (C), 135.31 (CH), 129.63 (CH), 128.46 (CH), 127.84 (C), 123.16 (CH), 121.08 (C), 117.48 (CH), 81.30 (2CH), 75.47 (CH), 74.85 (CH), 73.42 (2CH), 69.69 (CH), 68.49 (CH), 67.88 (CH), 67.81 (CH), 62.34 (CH_2_), 61.55 (CH_2_), 19.73 (3CH3), 19.68 (2CH3), 19.61 (2CH3), 19.20 (CH3); HR-MS (ESI positive, *m*/*z*): found 860.2056 ([M + Na]^+^), calc. for C_37_H_43_NO_19_NaS (M + Na): 860.2048.

*(2R,3R,4S,5R,6S)-2-((benzoyloxy)methyl)-6-((2-oxo-1-((2R,3R,5R,6R)-3,4,5-triacetoxy-6-(acetoxymethyl)tetrahydro-2H-pyran-2-yl)-1,2-dihydroquinolin-3-yl)thio)tetrahydro-2H-pyran-3,4,5-triyl tribenzoate* **3c**: Following procedure C, 3-iodo *N*-β-glycosylquinolinone β-**2d** (0.0831 mmol, 50 mg) and *O*-benzoylated 1-thio-β-d-glucopyranose **1d** (0.2077 mmol, 128 mg) were stirred at room temperature for 3 h. The crude product was purified by silica gel flash chromatography (Cyclohexan/EtOAc: 5/5), and the product **3c** was isolated as a pale yellow solid (52 mg, yield 58%); m.p.: 221–249 °C; R*f* = 0.4 (Cyclohexan/EtOAc: 5/5); ${\left[\alpha \right]}_{D}^{17}$ + 86.95 (c, 1.0 in CHCl_3_); IR (neat): 1728, 1648, 1597, 1452, 1367, 1277,1215, 1111,1086, 1068,801,766,708 cm^−1^; ^1^H-NMR (300 MHz, Acetone-*d*_6_) δ 8.24 (d, *J* = 8.4 Hz, 1H), 8.05–7.92 (m, 7H), 7.88–7.83 (m, 2H), 7.63–7.38 (m, 14H), 7.19 (t, *J* = 7.3 Hz, 1H), 6.88 (d, *J* = 9.7 Hz, 1H), 6.21 (t, *J* = 9.3 Hz, 1H), 5.93–5.78 (m, 4H), 5.57–5.38 (m, 2H), 4.89–4.72 (m, 2H), 4.59 (dd, *J* = 12.3, 6.2 Hz, 1H), 4.39–4.25 (m, 3H), 2.09–2.02 (m, 9H), 1.93 (s, 3H);^13^C-NMR (75 MHz, Acetone-*d*_6_) δ 169.73, 169.40, 169.14, 168.23, 168.84, 165.58, 165.17, 164.97, 164.77, 159.64, 135.85, 135.75, 135.04, 133.59, 133.54, 133.45, 133.18, 129.79, 129.5–128.53 (m), 123.06, 120.95, 117.36, 81.94, 81.30, 75.86, 74.81, 74.20, 73.27, 70.44, 69.59, 67.85, 67.77, 63.41, 61.55, 19.72, 19.58, 19.01; HR-MS (ESI positive, *m*/*z*): found 1086.2856 ([M + H]^+^), calc. for C_57_H_52_NO_19_S (M + H): 1086.2854.

*(2R,3R,5R,6R)-2-(3-(((2S,3R,4R,5S,6R)-3-acetamido-4,5-diacetoxy-6-(acetoxymethyl)tetrahydro-2H-pyran-2-yl)thio)-2-oxoquinolin-1(2H)-yl)-6-(acetoxymethyl)tetrahydro-2H-pyran-3,4,5-triyl triacetate* **3d**: Following procedure C, 3-iodo *N*-β-glycosylquinolinone β-**2d** (0.083 mmol, 50 mg) and *N*-acetyl-*O*-acetylated 1-thio-β-d-glucopyranose **1c** (0.2077mmol, 76 mg) were stirred at room temperature for 3 h. The crude product was purified by silica gel flash chromatography (dichloromethan/EtOAc: 2/8). The product **3d** was isolated as a white solid (62 mg, yield 90%); m.p.: 258.9–261.5 °C; R*f* = 0.28 (dichloromethan/EtOAc: 2/8); ${\left[\alpha \right]}_{D}^{17}$ + 20 (c, 1.0 in CHCl_3_); IR (neat): 1740, 1649, 1596, 1367, 1260, 1213,1076, 796,748 cm^−1^; ^1^H-NMR (400 MHz, CDCl_3_) δ 7.97 (s, 1H), 7.89 (d, *J* = 8.7 Hz, 1H), 7.51 (t, *J* = 7.5 Hz, 2H), 7.27–7.21 (m, 1H), 6.86 (d, *J* = 9.9 Hz, 1H), 6.75 (s, 1H), 5.88 (t, *J* = 9.4 Hz, 1H), 5.47–5.30 (m, 3H), 5.22–5.17 (m, 1H), 5.06 (t, *J* = 9.7 Hz, 1H), 4.25–4.03 (m, 6H), 3.81–3.74 (m, 1H), 2.09–1.85 (m, 24H); ^13^C-NMR (101 MHz, CDCl_3_) δ 170.77 (2C), 170.62 (C), 170.47 (C), 169.85 (C), 169.56 (C), 169.43 (C), 168.66, (C) 161.38 (C), 143.41 (CH), 136.63(C), 130.77 (CH), 129.30 (CH), 124.77 (C), 123.84 (CH), 120.99 (C), 116.84 (CH), 90.76 (CH), 82.98 (CH), 81.62 (CH), 76.03 (CH), 75.13 (CH), 73.85 (CH), 73.65 (CH), 70.79 (CH), 68.81 (CH), 68.04 (CH), 67.80 (CH), 62.44 (CH_2_), 61.62 (CH_2_), 23.51 (CH_3_), 23.10 (CH_3_), 21.00 (CH_3_), 20.77 (CH_3_), 20.65 (CH_3_), 20.63 (CH_3_), 20.59 (CH_3_), 19.98 (CH_3_); HR-MS (ESI positive, *m*/*z*): found 859.2211 ([M + Na]^+^), calc. for C_37_H_44_N_2_O_18_S (M + Na): 859.2208.

*(2R,4S,5R,6S)-2-(acetoxymethyl)-6-((2-oxo-1-((2R,3R,5S,6R)-3,4,5-trihydroxy-6-(hydroxymethyl)tetrahydro-2H-pyran-2-yl)-1,2-dihydroquinolin-3-yl)thio)tetrahydro-2H-pyran-3,4,5-triyl triacetate* **3e**: Following procedure C, 3-iodo *N*-β-glycosylquinolinone β-**2g** (0.115 mmol, 50 mg) and β-thiogalactose **1a** (0.2885 mmol, 106 mg) were stirred at room temperature for 3 h. After purification of the crude reaction by HPLC preparative (conditions: H_2_O + 0.1% AF/ACN gradient from 20% to 50% in 20 min), the product **3e** was isolated as a pale yellow solid (52 mg, yield 68%); m.p.: 244–252.8 °C; R*f* = 0.4 (Cyclohexane/EtOAc: 5/5); ${\left[\alpha \right]}_{D}^{17}$ − 16 (c, 1.0 in CHCl_3_); IR (neat): 1750, 1633, 1561, 1367, 1211, 1046, 732 cm^−1^; ^1^H-NMR (300 MHz, Acetone-*d*_6_) δ 8.10 (d, *J* = 8.1 Hz, 1H), 7.92 (s, 1H), 7.65 (d, *J* = 7.6 Hz, 1H), 7.48 (t, *J* = 7.7 Hz, 1H), 7.28 (t, *J* = 7.5 Hz, 1H), 6.59 (d, *J* = 9.7 Hz, 1H), 5.55 (d, *J* = 2.9 Hz, 1H), 5.47–5.27 (m, 4H), 4.54 (t, *J* = 6.1 Hz, 1H), 4,33 (t, J=8 Hz,1H), 4.24–4.12 (m, 3H), 3.97–3.56 (m, 7H), 2.08–2.03 (m, 12H);^13^C-NMR (75 MHz, Acetone-*d*_6_) δ 169.95 (C), 169.79 (C), 169.36 (C), 159.99 (C), 135.96 (C), 133.46 (CH), 129.20 (C), 128.81 (CH), 128.02 (CH), 122.70 (CH), 121.35 (C), 118.83 (C), 117.83 (CH), 84.04 (CH), 81.38 (CH), 80.44 (CH), 78.57 (CH), 74.45 (CH), 71.65 (CH), 70.16 (CH), 69.28 (CH), 67.67(CH), 66.68(CH), 62.05 (CH_2_), 61.57 (CH_2_), 19.80 (2CH_3_), 19.74 (CH_3_), 19.63 (CH_3_); HR-MS (ESI positive, *m*/*z*): found 692.1636 ([M + Na]^+^), calc. for C_29_H_35_NO_15_NaS (M + Na): 692.1625.

*(2R,3R,4S,6S)-2-(acetoxymethyl)-6-((4-(4-methoxyphenyl)-2-oxo-1-((2R,3R,5R,6R)-3,4,5-triacetoxy-6-(acetoxymethyl)tetrahydro-2H-pyran-2-yl)-1,2-dihydroquinolin-3-yl)thio)tetrahydro-2H-pyran-3,4,5-triyl triacetate* **3f**: Following procedure C, 3-iodo-*N*-β-glycosylquinolinone β-**2f** (0.071 mmol, 50 mg) and β-thioglucose **1a** (0.1767 mmol, 65 mg) were stirred at room temperature for 3 h. The crude product was purified by silica gel flash chromatography (EtOAc/Cyclohexane: 5/5), and the product **3f** was isolated as a pale brown solid (31 mg, yield 47%); m.p.: 143.4–150.3 °C; R*f* = 0.36 (EtOAc/Cyclohexane: 5/5); ${\left[\alpha \right]}_{D}^{19}$ − 1.42 (c, 1.0 in CHCl_3_); IR (neat): 1756, 1367, 1277, 1260, 1035, 766, 749 cm^−1^; ^1^H-NMR (400 MHz, Acetone *d*_6_) δ 8.31 (d, *J* = 8.7 Hz, 1H), 7.63 (t, *J* = 8.4 Hz, 1H), 7.27 (dd, *J* = 9.1, 1.9 Hz, 1H), 7.19–7.11 (m, 3H), 7.07–7.01 (m, 3H), 6.04 (t, *J* = 9.5 Hz, 1H), 5.59 (t, *J* = 9.4 Hz, 1H), 5.49 (t, *J* = 10.3 Hz, 1H), 5.38–5.32 (m, 2H), 5.16 (t, *J* = 9.6 Hz, 1H), 5.06 (t, *J* = 9.8 Hz, 1H), 4.98–4.92 (m, 2H), 4.85 (t, *J* = 9.7 Hz, 1H), 4.35–4.30 (m, 2H), 4.17 (dd, *J* = 12.5, 2.3 Hz, 1H), 3.89 (s, 3H), 2.07 (d, *J* = 4.5 Hz, 11H), 2.00 (s, 3H), 1.96 (d, *J* = 8.2 Hz, 10H); ^13^C-NMR (101 MHz, Acetone) δ 170.88, 170.66, 170.62, 170.24, 170.18, 170.06, 169.99, 169.94, 169.90, 169.69, 169.47, 131.62, 131.57, 131.24, 129.86, 129.63, 123.66, 122.91, 118.33, 114.67, 114.16, 87.75, 82.88, 82.18, 76.70, 75.95, 75.85, 74.49, 74.36, 74.19, 71.92, 70.34, 69.45, 68.98, 68.77, 68.59, 62.97, 62.53, 55.59, 20.79, 20.65, 20.55, 20.50, 20.40; HR-MS (ESI positive, *m*/*z*): found 966.2470 ([M + Na]^+^), calc. for C_44_H_49_NO_20_NaS (M + Na): 966.2466.

*(2R,3R,4S,5R,6S)-2-(acetoxymethyl)-6-(((2R,3R,4S,5R,6S)-4,5-diacetoxy-2-(acetoxymethyl)-6-((2-oxo-1-((2R,3R,5R,6R)-3,4,5-triacetoxy-6-(acetoxymethyl)tetrahydro-2H-pyran-2-yl)-1,2-dihydroquinolin-3-yl)thio)tetrahydro-2H-pyran-3-yl)oxy)tetrahydro-2H-pyran-3,4,5-triyl triacetate* **3g**: Following procedure C, 3-iodo *N*-β-glycosylquinolinone β-**2d** (0.083 mmol, 50 mg) and β-thiocellebiose **1e** (0.2077 mmol, 136 mg) were stirred at room temperature for 3 h. After purification of the crude reaction by HPLC preparative (conditions: H_2_O + 0.1% AF/ACN gradient from 40% to 100% in 15 min), the product **3g** was isolated as a pale yellow solid (92 mg, yield 98%): m.p.: 154.3–157.8 °C; R*f* = 0.53 (dichloromethane/EtOAc: 6/4); ${\left[\alpha \right]}_{D}^{19}$ − 15 (c, 1.0 in CHCl_3_); IR (neat): 1755, 1649, 1595, 1397, 1229, 1035,910 cm^−1^; ^1^H-NMR (400 MHz, CDCl_3_) δ 7.90 (d, *J* = 8.6 Hz, 1H), 7.63 (s, 1H), 7.52–7.43 (m, 2H), 7.26–7.22 (m, 1H), 6.81 (d, *J* = 9.9 Hz, 1H), 5.86 (t, *J* = 9.4 Hz, 1H), 5.41 (t, *J* = 9.3 Hz, 1H), 5.33 (t, *J* = 9.8 Hz, 1H), 5.24 (t, *J* = 8.7 Hz, 1H), 5.12–5.03 (m, 3H), 4.96 (d, *J* = 10.0 Hz, 1H), 4.90 (t, *J* = 8.6 Hz, 1H), 4.64–4.52 (m, 1H), 4.49 (d, *J* = 7.8 Hz, 1H), 4.42 (d, *J* = 11.3 Hz, 1H), 4.33 (dd, *J* = 12.5, 4.5 Hz, 1H), 4.26–4.19 (m, 1H), 4.09 (dd, *J* = 11.9, 5.9 Hz, 1H), 4.05–3.96 (m, 2H), 3.79–3.71 (m, 2H), 3.68–3.61 (m, 1H), 2.08–2.03 (m, 10H), 2.01–1.93 (m, 23H). ^13^C-NMR (101 MHz, CDCl_3_) δ 170.57 (C), 170.49 (C), 170.33 (C), 170.27 (C), 169.86 (C), 169.81 (C), 169.73 (C), 169.48 (C), 169.40 (C), 169.14 (C), 169.08 (C), 160.26 (C), 137.79 (CH), 136.19 (C), 130.16 (CH), 128.73 (CH), 126.93 (C), 123.62 (CH), 121.04 (C), 116.84 (CH), 100.93 (CH), 82.07 (CH), 81.52 (CH), 76.94 (CH), 76.69 (CH), 75.21 (CH), 73.79 (CH), 73.62 (CH), 73.01 (CH), 72.15 (CH),71.76 (CH), 70.32 (CH), 67.95 (2CH), 67.84 (CH), 62.52 (CH_2_), 61.75 (CH_2_), 61.61 (CH_2_), 20.83 (CH_3_), 20.78 (CH_3_), 20.75 (CH_3_), 20.73 (CH_3_), 20.69 (CH_3_), 20.62 (4CH_3_), 20.23 (CH_3_), 1.10 (CH_3_); HR-MS (ESI positive, *m*/*z*): found 1148.2888 ([M + Na]^+^), calc. for C_49_H_59_NO_27_NaS (M + Na): 1148.2893.

*(2R,3R,4S,5R,6S)-2-(acetoxymethyl)-6-(((2R,3R,4S,5R,6S)-4,5-diacetoxy-2-(acetoxymethyl)-6-((2-oxo-1-((2R,3R,5S,6R)-3,4,5-trihydroxy-6-(hydroxymethyl)tetrahydro-2H-pyran-2-yl)-1,2-dihydroquinolin-3-yl)thio)tetrahydro-2H-pyran-3-yl)oxy)tetrahydro-2H-pyran-3,4,5-triyl triacetate* **3h**: Following procedure C, 3-iodo *N*-β-glycosylquinolinone β-**2g** (0.115 mmol, 50 mg) and β-thiocellobiose **1e** (0.2885 mmol, 189 mg) were stirred at room temperature for 3 h. After purification of the crude reaction by HPLC preparative (conditions: H_2_O + 0.1% AF/ACN gradient from 20% to 50% in 15 min), the product **3h** was isolated as a white solid (63 mg, yield 57%); m.p.: 249.2–251.9 °C; R*f* = 0.52 (EtOAc/MeOH: 9/1); ${\left[\alpha \right]}_{D}^{17}$ + 7.14 (c, 1.0 in CHCl_3_); IR (neat): 1740, 1629, 1590, 1366, 1212, 1033, 907, 751 cm^−1^; ^1^H-NMR (300 MHz, Acetone-*d*_6_) δ 8.09 (d, *J* = 8.0 Hz, 1H), 7.82 (s, 1H), 7.65 (d, *J* = 7.6 Hz, 1H), 7.48 (t, *J* = 7.90 Hz, 1H), 7.28 (t, *J* = 7.3 Hz, 1H), 6.58 (d, *J* = 9.7 Hz, 1H), 5.43–5.23 (m, 4H), 5.14–5,03 (m, 2H), 4.94–4.84 (m, 2H), 4.60–4.30 (m, 6H), 4.25–4.14 (m, 3H), 3.96–3.56 (m, 8H), 2.05–1.92 (m, 16H); ^13^C-NMR (75 MHz, Acetone-*d*_6_) δ 169.97 (C), 169.83 (C), 169.41 (C), 169.31 (C), 169.23 (C), 169.03 (C), 168.82 (C), 159.87 (C), 135.88 (C), 132.79 (CH), 130.77 (C), 128.77 (CH), 128.00 (CH), 122.63 (CH), 121.34 (C), 117.80 (CH), 100.53 (CH), 84.05 (CH), 80.55 (CH), 80.46 (CH), 78.56 (CH), 76.73 (CH), 76.45 (CH), 73.33 (CH), 72.78 (CH), 71.57 (CH), 71.53(CH), 70.25 (CH), 69.80 (CH), 69.35 (CH), 68.03 (CH), 62.69 (CH_2_), 61.67 (CH_2_), 61.57 (CH_2_), 19.93 (CH_3_), 19.84 (CH_3_), 19.76 (CH_3_), 19.71 (CH_3_), 19.69 (CH_3_), 19.61 (CH_3_), 19.52 (CH_3_); HR-MS (ESI positive, *m*/*z*): found 980.2461 ([M + Na]^+^), calc. for C_41_H_51_NO_23_NaS (M + Na): 980.2470.

*(2R,3R,4S,5R,6R)-2-(acetoxymethyl)-6-(((2R,3S,4S,5R,6R)-4,5-diacetoxy-2-(acetoxymethyl)-6-(((2R,3S,4S,5R,6S)-4,5-diacetoxy-2-(acetoxymethyl)-6-((2-oxo-1-((2R,3R,5R,6R)-3,4,5-triacetoxy-6-(acetoxymethyl)tetrahydro-2H-pyran-2-yl)-1,2-dihydroquinolin-3-yl)thio)tetrahydro-2H-pyran-3-yl)oxy)tetrahydro-2H-pyran-3-yl)oxy)tetrahydro-2H-pyran-3,4,5-triyl triacetate* **3i**: Following procedure C, 3-iodo *N*-β-glycosylquinolinone β-**2d** (0.083 mmol, 50 mg) and β-thiomalthotriose **1f** (0.2077mmol, 196 mg) were stirred at room temperature for 3 h. After purification of the crude reaction by HPLC preparative (conditions: H_2_O + 0.1% AF/MeOH gradient from 50% to 100% in 15 min), the product **3i** was isolated as a white solid (116.25 mg, yield 98%); m.p.: 110.8–134.3 °C; R*f* = 0.19 (Cyclohexane/EtOAc: 4/6); ${\left[\alpha \right]}_{D}^{17}$ + 40 (c, 1.0 in CHCl_3_); IR (neat): 1756, 1649, 1367, 1260, 1208, 1011, 794, 708, 702 cm^−1^; ^1^H-NMR (300 MHz, CDCl_3_) δ 7.93 (d, *J* = 8.6 Hz, 1H), 7.73 (s, 1H), 7.54 (t, *J* = 9.1 Hz, 2H), 7.31 (d, *J* = 7.4 Hz, 1H), 6.85 (d, *J* = 9.9 Hz, 1H), 5.91 (t, *J* = 9.5 Hz, 1H), 5.45–5.27 (m, 8H), 5.12–4.94 (m, 4H), 4.86 (dd, *J* = 10.5, 4.0 Hz, 1H), 4.75 (dd, *J* = 10.3, 4.0 Hz, 1H), 4.49–4.18 (m, 8H), 3.99–3.89 (m, 4H), 2.12–1.96 (m, 42H); ^13^C-NMR (75 MHz, CDCl_3_) δ 170.68, (C) 170.59 (C), 170.52 (C), 170.39 (C), 170.18 (C), 170.12 (C), 170.00 (C), 169.86 (C), 169.75 (C), 169.69 (C), 169.61 (C), 169.51 (C), 169.39 (C), 168.97 (C), 144.13 (C), 136.14 (C), 130.09 (CH), 126.52 (C), 123.61 (CH), 120.99 (C), 116.67 (CH), 95.90 (CH), 95.75 (CH), 81.76 (CH), 81.43 (CH), 76.15 (CH), 75.98 (CH), 75.76 (CH), 75.13 (CH), 74.17 (CH), 73.72 (CH), 72.67 (CH), 71.71 (CH), 70.81 (CH), 70.36 (CH), 70.06 (CH), 69.38 (CH), 69.02 (CH), 68.59 (CH), 67.89 (CH), 68.19 (CH), 67.78 (CH), 67.56 (CH), 63.50 (CH_2_), 62.46 (CH_2_), 61.66 (CH_2_), 61.42 (CH_2_), 20.84 (2CH_3_), 20.81 (CH_3_), 20.77 (CH_3_), 20.56 (9CH_3_), 20.17 (CH_3_); HR-MS (ESI positive, *m*/*z*): found 1436.3730 ([M + Na]^+^), calc. for C_61_H_75_NO_35_NaS (M + Na): 1436.3738.

*(2R,3R,4S,5R,6S)-2-(acetoxymethyl)-6-((2-oxo-1-(((2R,3S,4R,5S,6S)-3,4,5-triacetoxy-6-(acetoxymethyl)tetrahydro-2H-pyran-2-yl)-1,2-dihydroquinolin-3-yl)thio)tetrahydro-2H-pyran-3,4,5-triyl triacetate* **3j**: Following procedure C, 3-iodo *N*-α-glycosylquinolinone α-**2e** (0.083 mmol, 50 mg) and β-thioglucose **1a** (0.2077 mmol, 76 mg) were stirred at room temperature for 3 h. After purification of the crude reaction by HPLC preparative (conditions: H_2_O + 0.1% AF/ACN gradient from 40 to 100% in 15 min), the product **3j** was isolated as a pale yellow solid (26 mg, yield 35%); m.p.: 224–225 °C; R*f* = 0.35 (EtOAc/heptan: 7/3); ${\left[\alpha \right]}_{D}^{19}$ + 4.44 (c, 1.0 in CHCl_3_); IR (neat): 1755, 1641, 1367, 1259, 1031, 913, 799, 748 cm^−1^; ^1^H-NMR (400 MHz, Acetone-*d*_6_) δ 7.87–7.77 (m, 2H), 7.54 (d, *J* = 7.8 Hz, 1H), 7.44 (t, *J* = 8.07 Hz, 1H), 7.19 (t, *J* = 7.5 Hz, 1H), 6.71 (d, *J* = 5.9 Hz, 1H), 5.84 (t, *J* = 6.12 Hz,1H), 5.33–5.22 (m, 3H), 5.08 (t, *J* = 7.3 Hz, 1H), 5.01 (dd, *J* = 18.4, 9.4 Hz, 2H), 4.57–4.52 (m, 1H), 4.34 (dd, *J* = 12.5, 5.8 Hz, 1H), 4.18–4.05 (m, 4H), 1.97 (s, 2H), 1.93–1.88 (m, 13H), 1.86–1.82 (m, 9H); ^13^C-NMR (101 MHz, Acetone-*d*_6_) δ 170.71 (C), 170.70 (C), 170.29 (C), 170.19 (C), 170.03 (C), 170.01 (C), 169.96 (C), 169.92 (C), 161.31 (C), 138.86 (C), 136.24 (CH), 130.31 (CH), 129.88 (C), 128.96 (CH), 124.01 (CH), 122.29 (C), 117.77 (CH),82.25 (CH), 80.36 (CH), 76.37 (CH), 74.67 (CH), 74.37 (CH), 70.52 (CH), 70.41, 69.38, 68.58, 63.26 (CH_2_), 62.23 (CH_2_), 20.83 (CH_3_), 20.79 (CH_3_), 20.70 (CH_3_), 20.64 (CH_3_), 20.62 (CH_3_), 20.60 (CH_3_), 20.53 (CH_3_), 20.30 (CH_3_); HR-MS (ESI positive, *m*/*z*): found 860.2042 ([M + Na]^+^), calc. for C_37_H_43_NO_19_NaS (M + Na): 860.2048.

*1-((2R,3R,5S,6R)-3,4,5-trihydroxy-6-(hydroxymethyl)tetrahydro-2H-pyran-2-yl)-3-(((2S,3R,4S,5S,6R)-3,4,5-trihydroxy-6-(hydroxymethyl)tetrahydro-2H-pyran-2-yl)thio)quinolin-2(1H)-one* **4a**: Following procedure D, a mixture of **3b** (20 mg, 1.0 equiv.) and K_2_CO_3_ (2 mg, 0.5 equiv.) in methanol (2 mL) was stirred under argon at room temperature for 1 h. The crude mixture was then filtered through celite, washed with 10 mL of methanol, and filtered again. The filtrate was concentrated under reduced pressure at 25. The product **4a** was isolated as a white solid (12 mg, yield 98%); m.p.: 187–197.8 °C; Rf = 0.52 (EtOAc/MeOH: 9/1); ${\left[\alpha \right]}_{D}^{19}$ − 63.15 (c, 1.0 in CHCl_3_); IR (neat): 1277, 1281, 798, 766, 749 cm^−1^; ^1^H-NMR (400 MHz, DMSO-*d*_6_) δ 7.95 (d, *J* = 8.6 Hz, 1H), 7.87 (s, 1H), 7.58 (d, *J* = 7.6 Hz, 1H), 7.45 (t, *J* = 8.0 Hz, 1H), 7.24 (t, *J* = 7.5 Hz, 1H), 6.31 (d, *J* = 9.7 Hz, 1H), 4.75 (d, *J* = 9.6 Hz, 1H), 4.04 (t, *J* = 9 Hz; 2H), 3.74 (d, *J* = 10.6 Hz, 3H), 3.24–3.13 (m, 15H); ^13^C-NMR (101 MHz, DMSO-*d*_6_) δ 160.08 (C), 135.24 (C), 133.12 (CH), 130.37 (C), 128.51 (CH), 128.23 (CH), 122.82 (CH), 121.52 (C), 117.63 (CH), 84.15 (CH), 84.02 (CH), 81.38 (CH), 80.89 (CH), 78.52 (CH), 78.18 (CH), 72.78 (CH), 70.22 (CH), 69.82 (CH), 68.91 (CH), 61.20 (CH_2_), 61.08 (CH_2_); HR-MS (ESI positive, *m*/*z*): found 524.1205 ([M + Na]^+^), calc. for C_21_H_27_NO_11_NaS (M + Na): 524.1203.

## 4. Conclusions

In summary, we have successfully developed an efficient method to synthesize various *bis* β-*N*,*S*-glycosyl quinolin-2-ones via the palladium-catalyzed coupling of α- or β-mono-, di-, and poly-thiosugar derivatives with α- or β-3-iodo-*N*-glycosylquinolin-2-ones. Efforts are now in progress to synthesize a large library of analogues through this strategy to study their biological activity. We expect this simple and general methodology to be of broad utility for the synthesis and development of new medicinal agents.

## Figures and Tables

**Table 1:** Survey of reaction conditions for the coupling of tetra-*O*-acetylated 1-thio-β-d-galactopyranose **1a** with 3-halo *N*-glucosylquinolinone β-**2a**,**d**.

Entry	Comp. β-2	1a (equiv.)	Time (h)	Temperature (°C)	Conversion Rate *^a^*	Yield (%) *^b^*
1	**2a**	1.5	5	r.t.	0	-
2	**2a**	1.5	5	60	36%	-
3	**2a**	2.5	1	100	42%	-
4	**2d**	2.5	3	r.t.	100%	70%
5	**2d**	1.5	3	r.t.	40%	-

*^a^* Conversion rate was determined by ^1^H-NMR in the crude reaction mixture based on the chemical shift (ppm) of the proton signal H^4^ for haloquinolinone β-**2a**,**b** (δ = 8.35) and **3a** (δ = 8.27); *^b^* Yield of isolated **3a**.

## Supplementary Materials

The supplementary materials are available online. Spectra for all synthesized compounds are available online.
