# Supplementary material for: Convergent Synthesis of N,S-bis Glycosylquinolin-2-ones via a Pd-G3-XantPhos Precatalyst Catalysis
Source: Molecules. 2018 Feb 26;23(3):519. doi: 10.3390/molecules23030519 (PMC6017768; doi:10.3390/molecules23030519)
Supplement: Supplementary file 1 [file molecules-23-00519-s001.pdf]

Convergent synthesis of -N-S- bis-glycosylquinolin-2-ones *via* a Pd-G3-XantPhos precatalyst catalysis

Wafa Radjdal,[2] Nada Ibrahim,[1] Belkacem Benmerad,[2] Mouad Alami[1] and Samir Messaoudi[1]

[1] Univ. Paris-Sud, CNRS, University Paris-Saclay, Châtenay-Malabry, France
nada.makky@u-psud.fr (N. I); mouad.alami@u-psud.fr (M.A)
[2] Laboratoire de Physico-Chimie des Matériaux et Catalyse, Faculté des Sciences Exactes, Université de Bejaia, 0600 Bejaia, Algeria. wafasmanalyse@gmail.com (W.R); benmeradbelka@yahoo.fr (B.B)

Supporting Information

Table of contents

^{1}H and ^{13}C NMR spectra of compounds 2a-g... 2
^{1}H and ^{13}C NMR spectra of compounds 3a-j... 15
^{1}H and ^{13}C NMR spectra of compounds 4a... 36

(2R,3R,4S,5R,6R)-2-(acetoxymethyl)-6-(3-bromo-2-oxoquinolin-1(2H)-yl)tetrahydro-2H-pyran-3,4,5-triyl triacetate ***2a*** **(in $CDCl_3$, 300 MHz):**

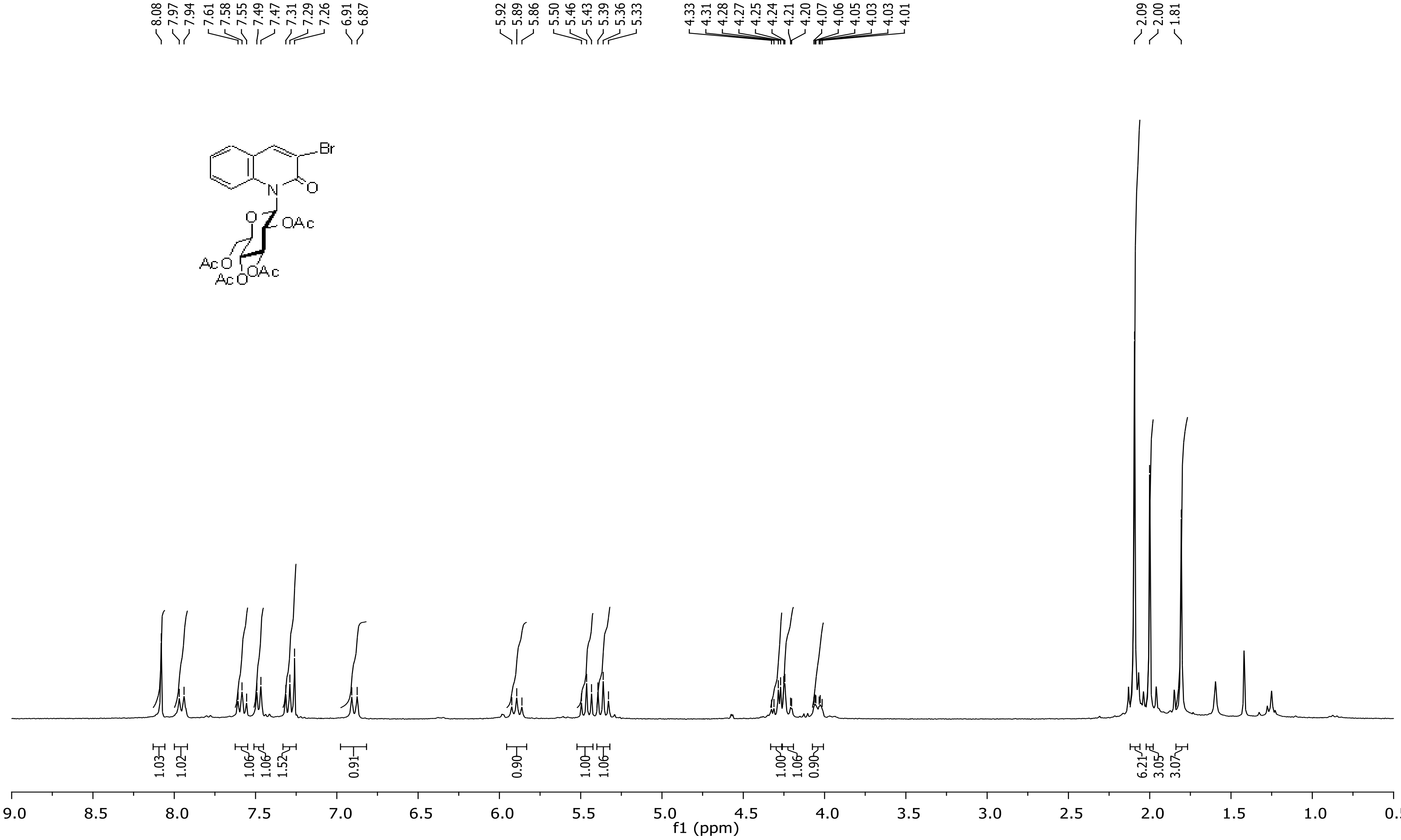

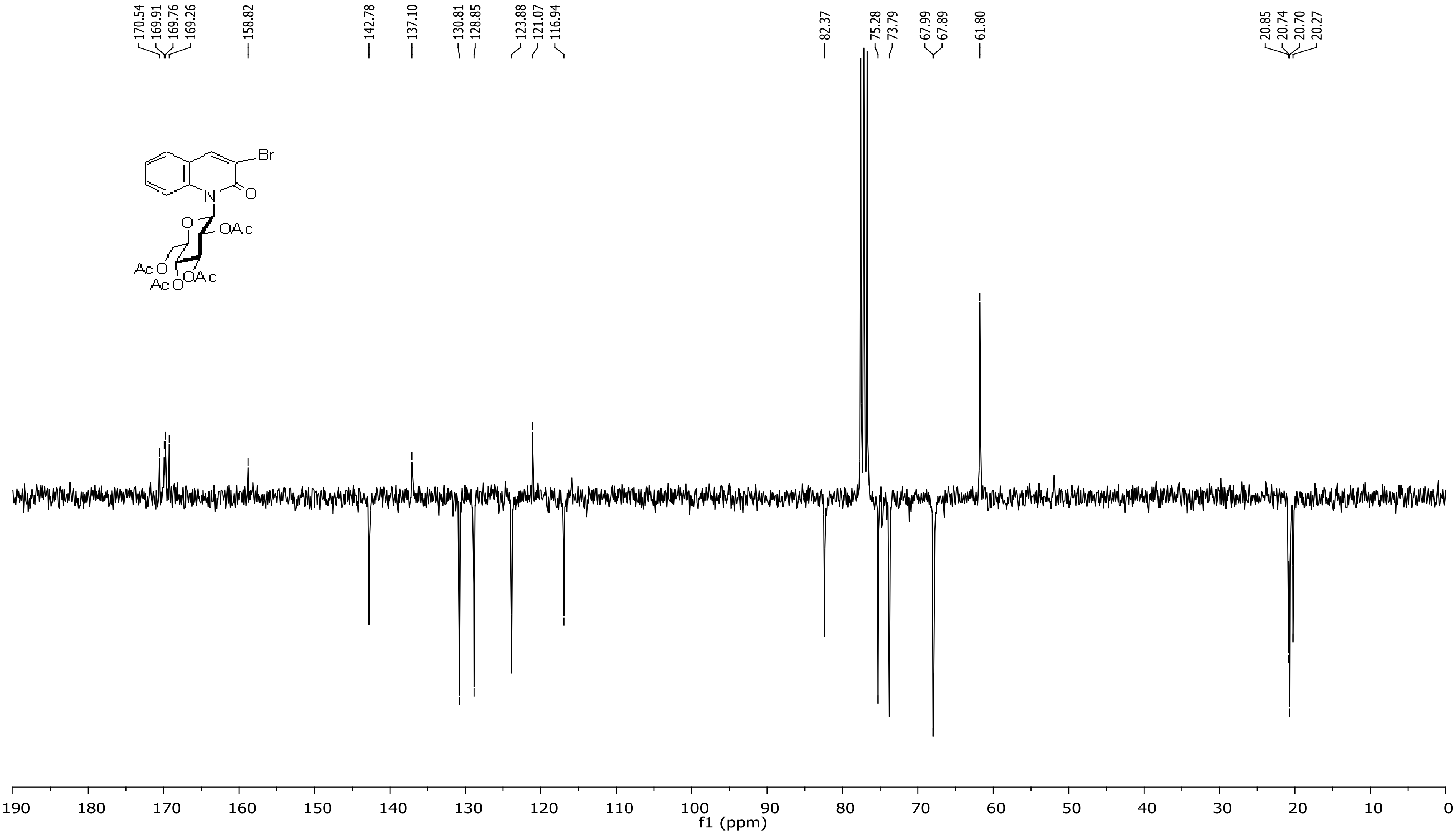
170.54
169.91
169.76
169.26
158.82
142.78
137.10
130.81
128.85
123.88
121.07
116.94
82.37
75.28
73.79
67.99
67.89
61.80
20.85
20.74
20.70
20.27
Br
N
O
OAc
AcO
AcO
OAc
190 180 170 160 150 140 130 120 110 100 90 80 70 60 50 40 30 20 10 0
f1 (ppm)

(2S,3S,4R,5S,6R)-2-(acetoxymethyl)-6-(3-bromo-2-oxoquinolin-1(2H)-yl)tetrahydro-2H-pyran-3,4,5-triyl triacetate **2b** **(in $CDCl_3$, 300 MHz):**

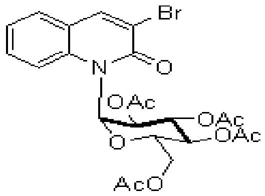

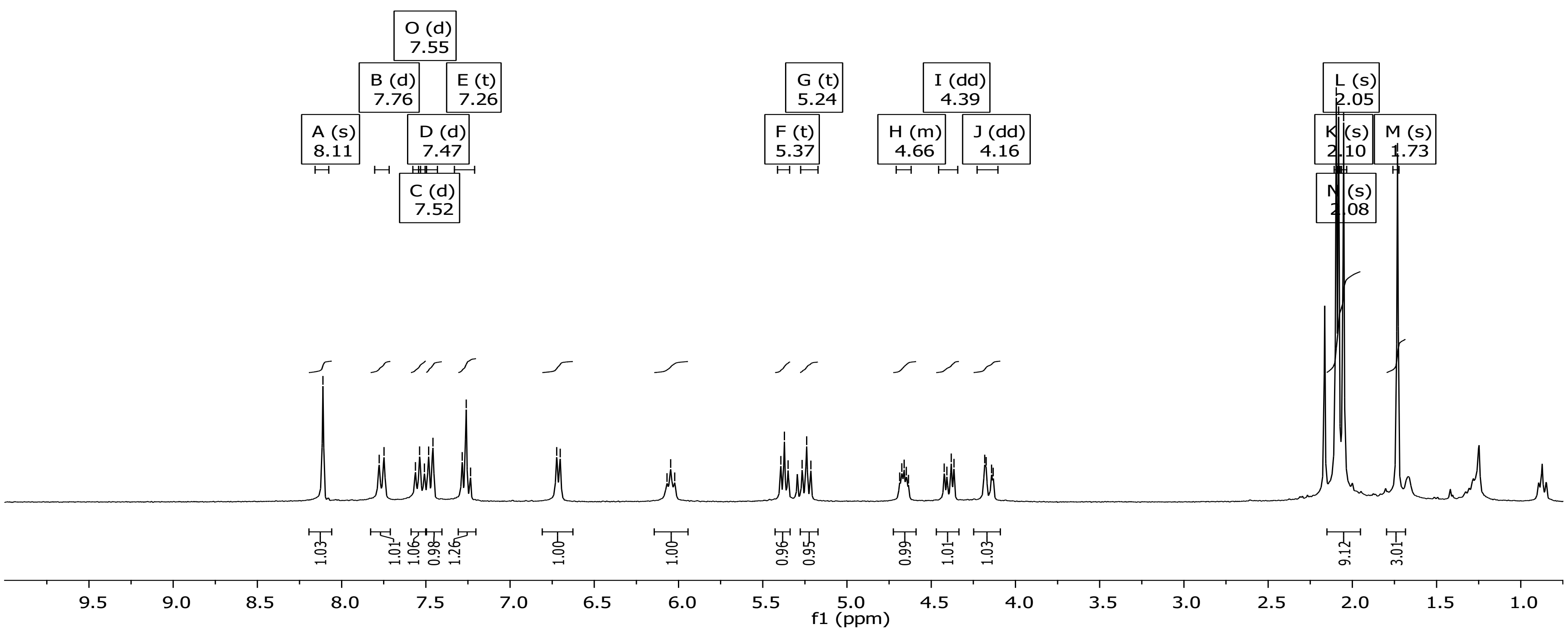

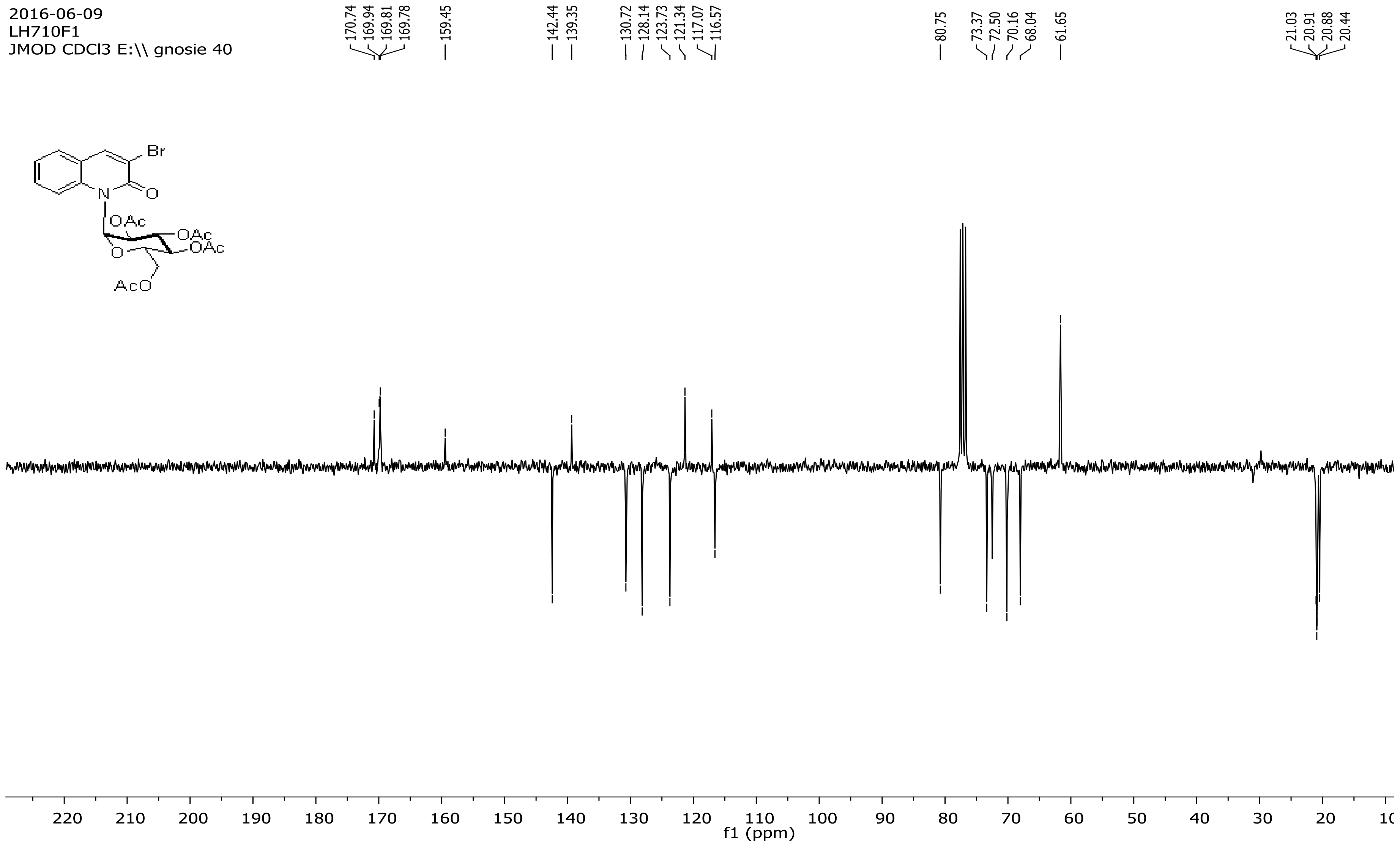
2016-06-09
LH710F1
JMOD CDCl3 E:\\ gnosie 40
170.74
169.94
169.81
169.78
159.45
142.44
139.35
130.72
128.14
123.73
121.34
117.07
116.57
80.75
73.37
72.50
70.16
68.04
61.65
21.03
20.91
20.88
20.44
Br
N
O
OAc
OAc
OAc
O
AcO
220 210 200 190 180 170 160 150 140 130 120 110 100 90 80 70 60 50 40 30 20 10
f1 (ppm)

(2R,3R,4S,5R,6R)-2-(acetoxymethyl)-6-(3-bromo-4-(4-methoxyphenyl)-2-oxoquinolin-1(2H)-yl)tetrahydro-2H-pyran-3,4,5-triyl triacetate ***2c*** **(in $CDCl_3$, 300 MHz):**

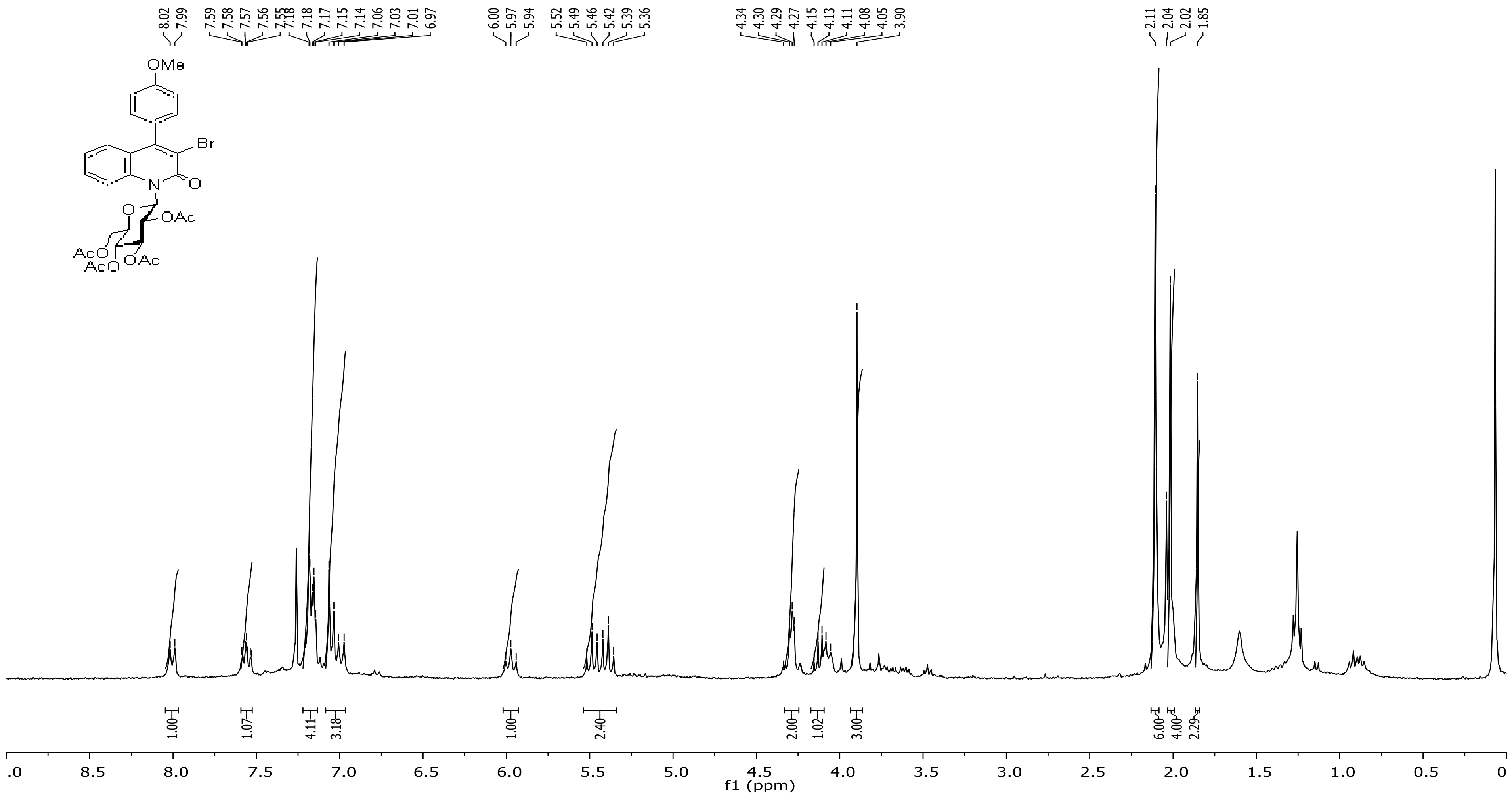

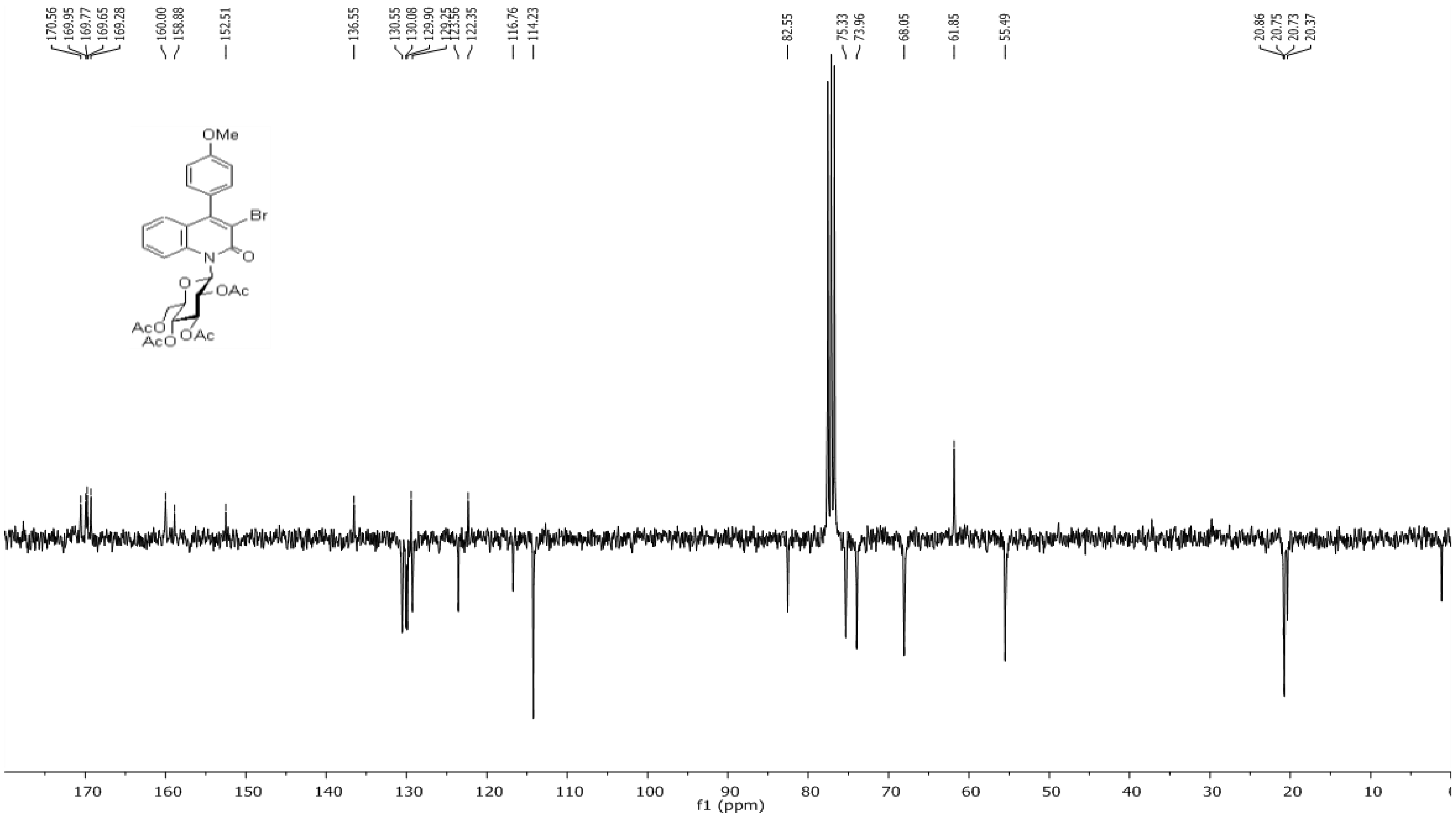
170.56
169.95
169.77
169.65
169.28
160.00
158.88
152.51
136.55
130.55
130.08
129.90
129.25
123.56
122.35
116.76
114.23
82.55
75.33
73.96
68.05
61.85
55.49
20.86
20.75
20.73
20.37
OMe
Br
N
O
OAc
AcO
AcO
OAc
170
160
150
140
130
120
110
100
90
80
70
60
50
40
30
20
10
f1 (ppm)

(2R,3R,4S,5R,6R)-2-(acetoxymethyl)-6-(3-iodo-2-oxoquinolin-1(2H)-yl)tetrahydro-2H-pyran-3,4,5-triyl triacetate **2d** **(in $CDCl_3$, 300 MHz):**

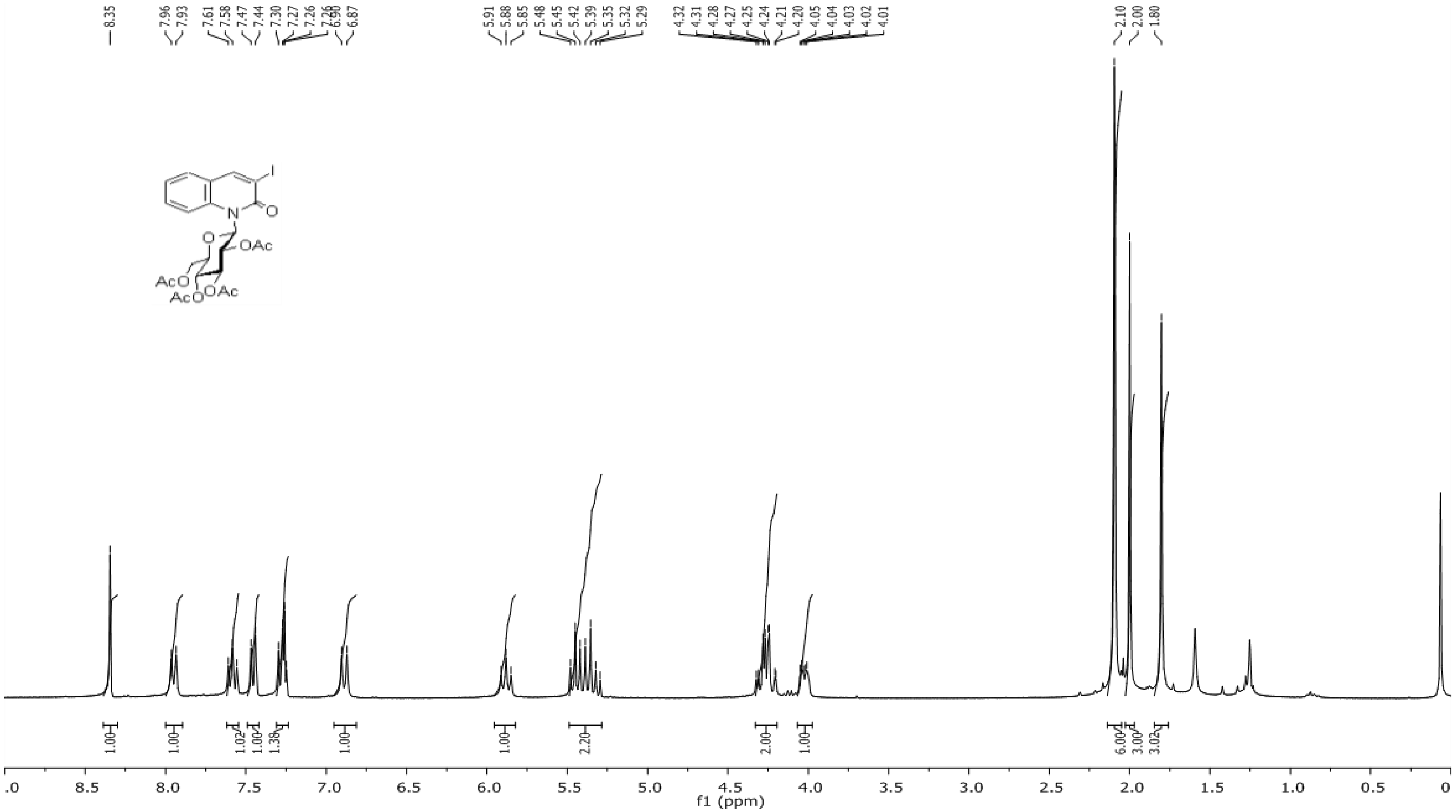

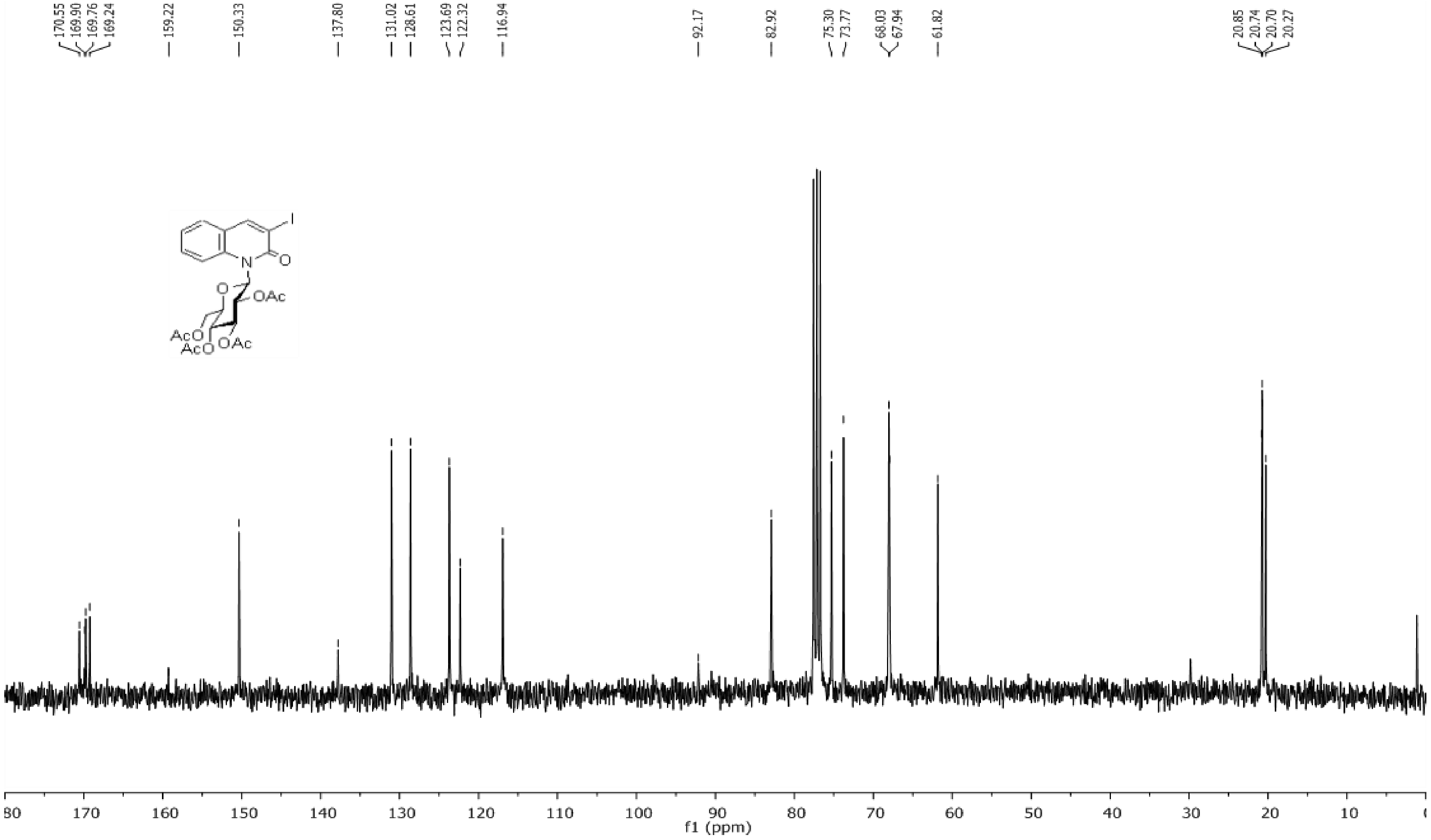
170.55
169.90
169.76
169.24
159.22
150.33
137.80
131.02
128.61
123.69
122.32
116.94
92.17
82.92
75.30
73.77
68.03
67.94
61.82
20.85
20.74
20.70
20.27
OAc
AcO
AcO
OAc
f1 (ppm)

(2S,3S,4R,5S,6R)-2-(acetoxymethyl)-6-(3-iodo-2-oxoquinolin-1(2H)-yl)tetrahydro-2H-pyran-3,4,5-triyl triacetate ***2e*** **(in $CDCl_3$, 300 MHz):**

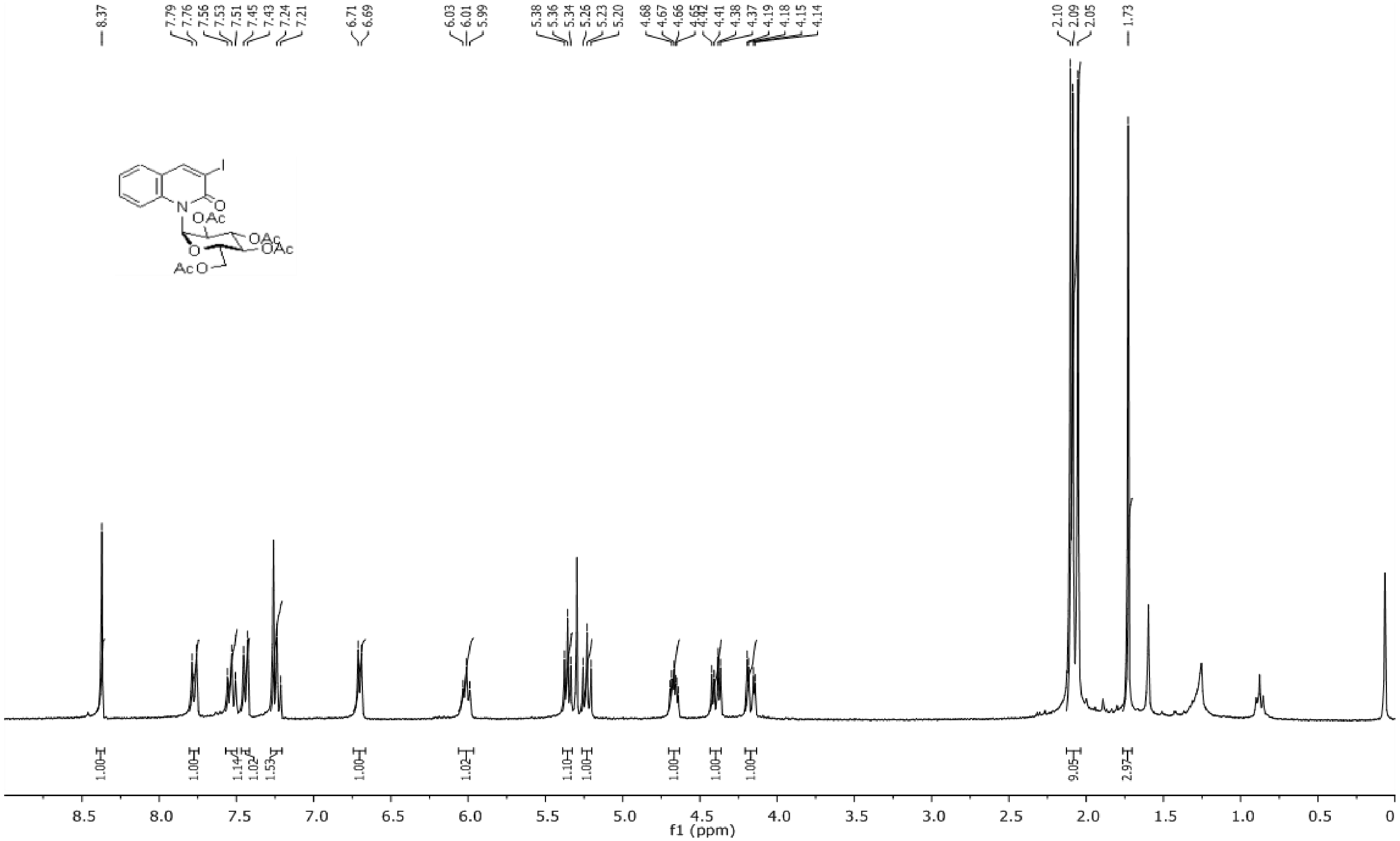

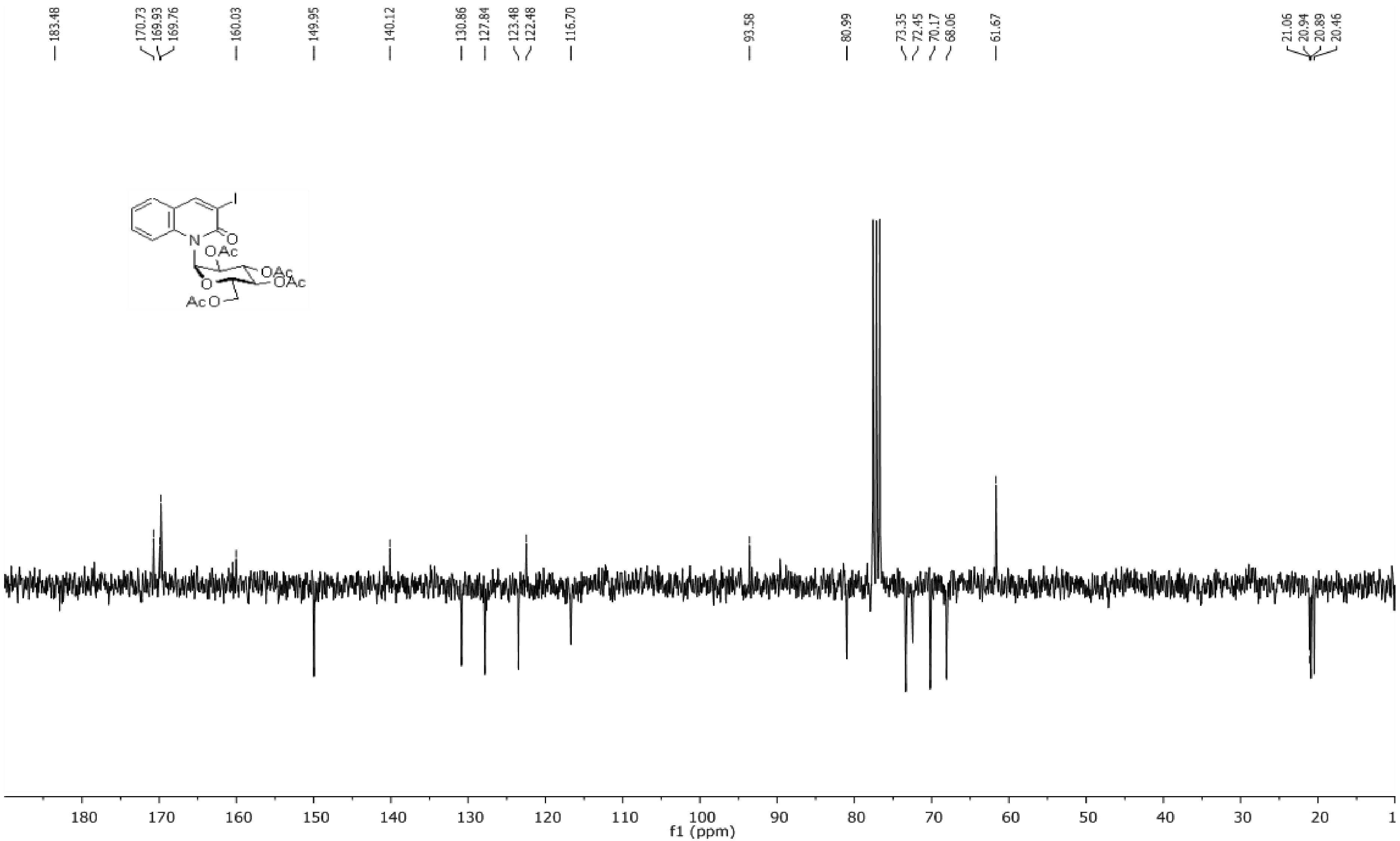
183.48
170.73
169.93
169.76
160.03
149.95
140.12
130.86
127.84
123.48
122.48
116.70
93.58
80.99
73.35
72.45
70.17
68.06
61.67
21.06
20.94
20.89
20.46
I
N
O
OAc
OAc
OAc
AcO
180 170 160 150 140 130 120 110 100 90 80 70 60 50 40 30 20 1
f1 (ppm)

(2R,3R,4S,5R,6R)-2-(acetoxymethyl)-6-(3-iodo-4-(4-methoxyphenyl)-2-oxoquinolin-1(2H)-yl)tetrahydro-2H-pyran-3,4,5-triyl triacetate **2f (in Acetone, 300 MHz):**

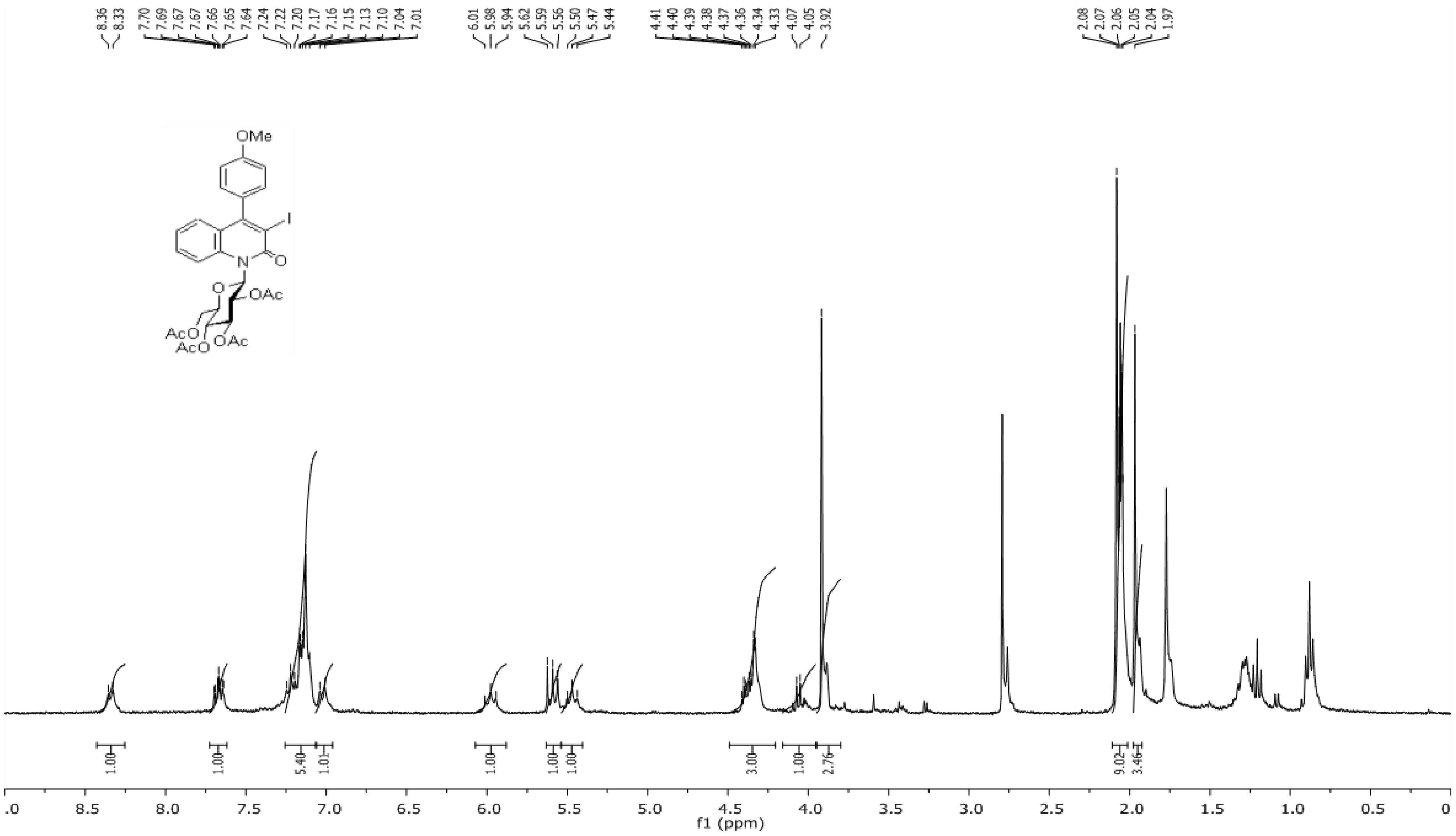

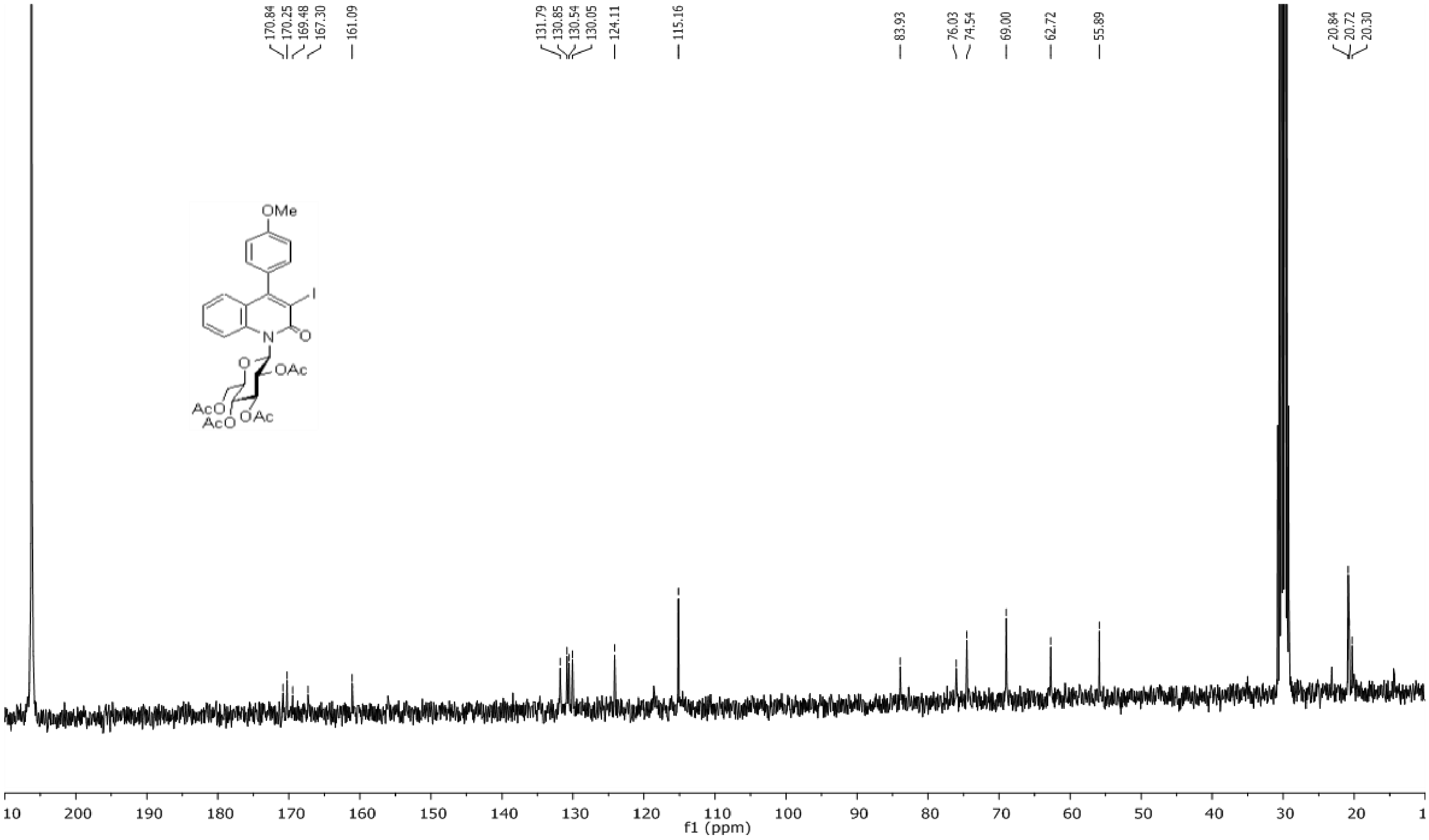

170.84
170.25
169.48
167.30
161.09
131.79
130.85
130.54
130.05
124.11
115.16
83.93
76.03
74.54
69.00
62.72
55.89
20.84
20.72
20.30
OMe
I
N
O
OAc
AcO
AcO
OAc
f1 (ppm)

3-iodo-1-((2R,3R,4S,5S,6R)-3,4,5-trihydroxy-6-(hydroxymethyl)tetrahydro-2H-pyran-2-yl)quinolin-2(1H)-one ***2g*** **(in MeOD), 300 MHz):**

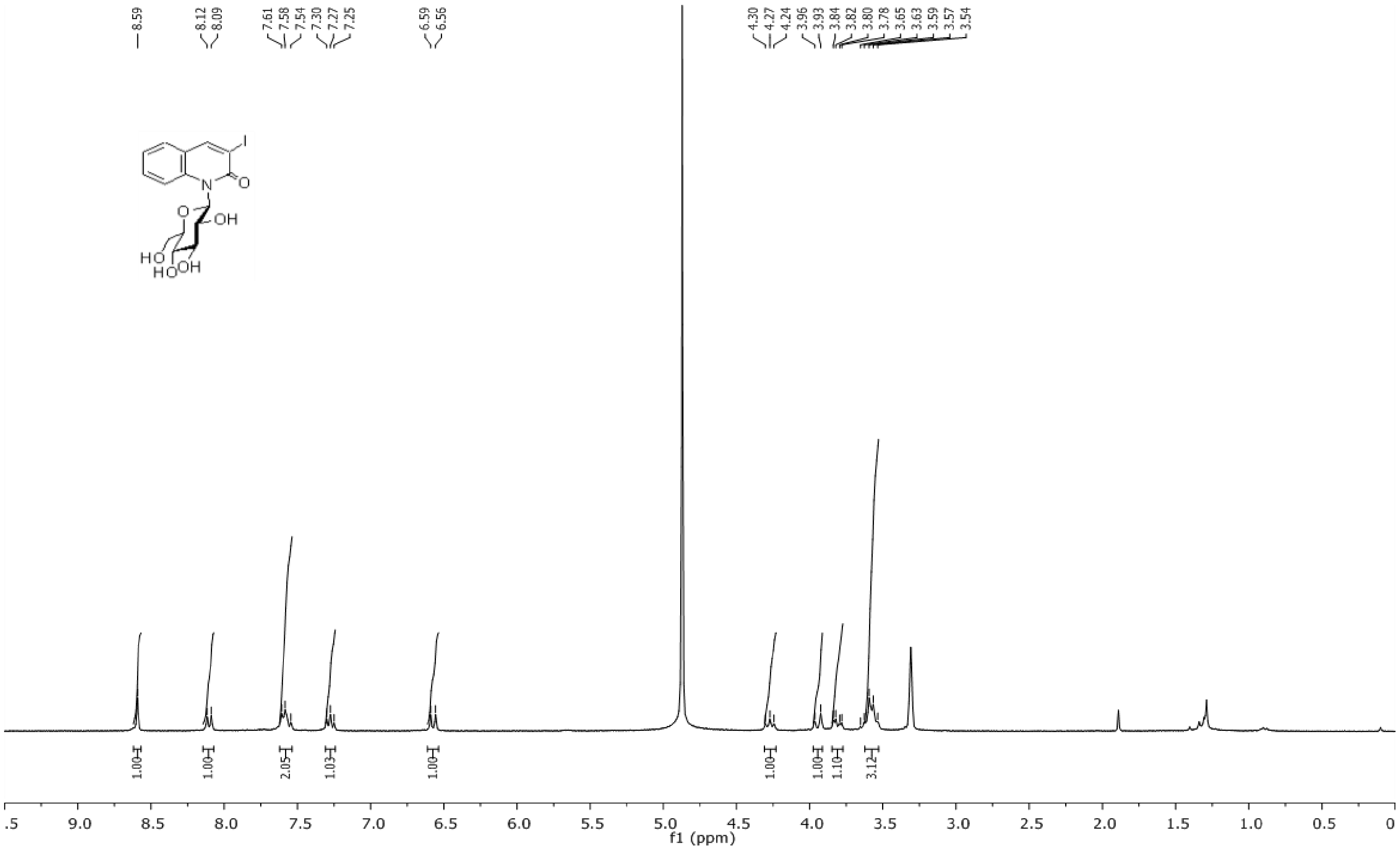

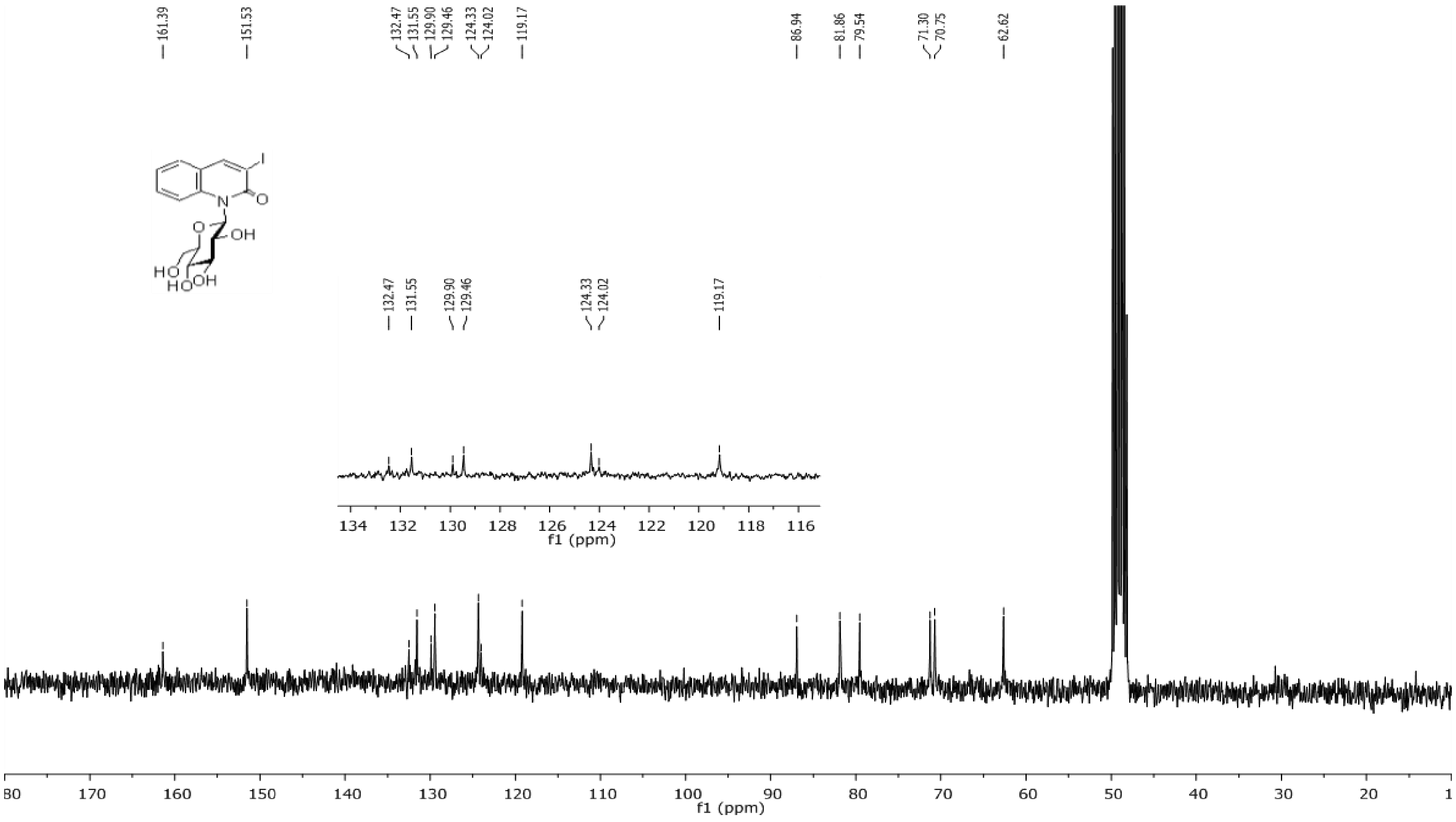
161.39
151.53
132.47
131.55
129.90
129.46
124.33
124.02
119.17
86.94
81.86
79.54
71.30
70.75
62.62
I
O
N
O
OH
HO
HO
OH
134 132 130 128 126 124 122 120 118 116
f1 (ppm)
80 170 160 150 140 130 120 110 100 90 80 70 60 50 40 30 20 1
f1 (ppm)

(2R,3S,4S,5R,6S)-2-(acetoxymethyl)-6-((2-oxo-1-((2R,3R,5R,6R)-3,4,5-triacetoxy-6-(acetoxymethyl)tetrahydro-2H-pyran-2-yl)-1,2-dihydroquinolin-3-yl)thio)tetrahydro-2H-pyran-3,4,5-triyl triacetate 3a **(in Acetone, 300 MHz):**

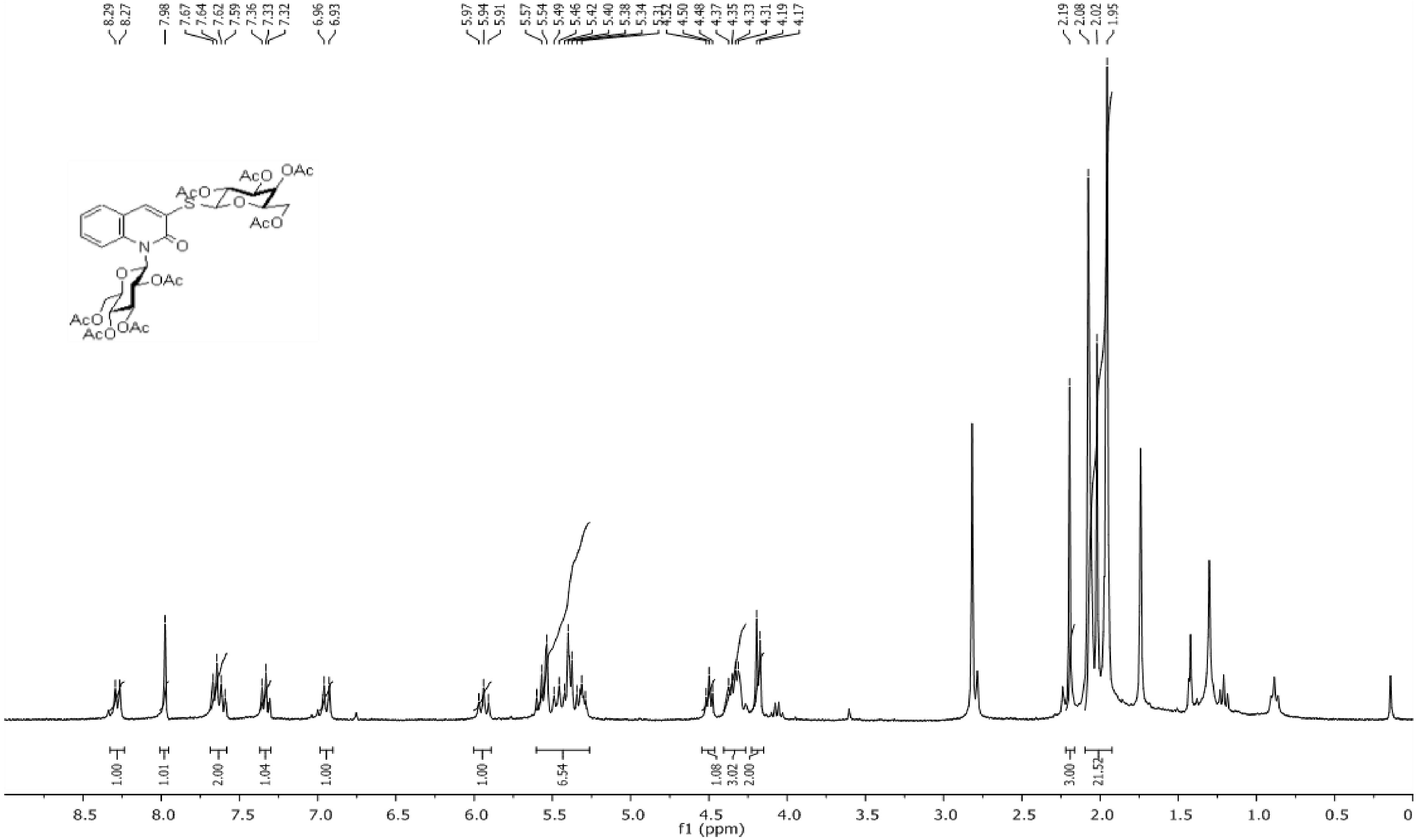

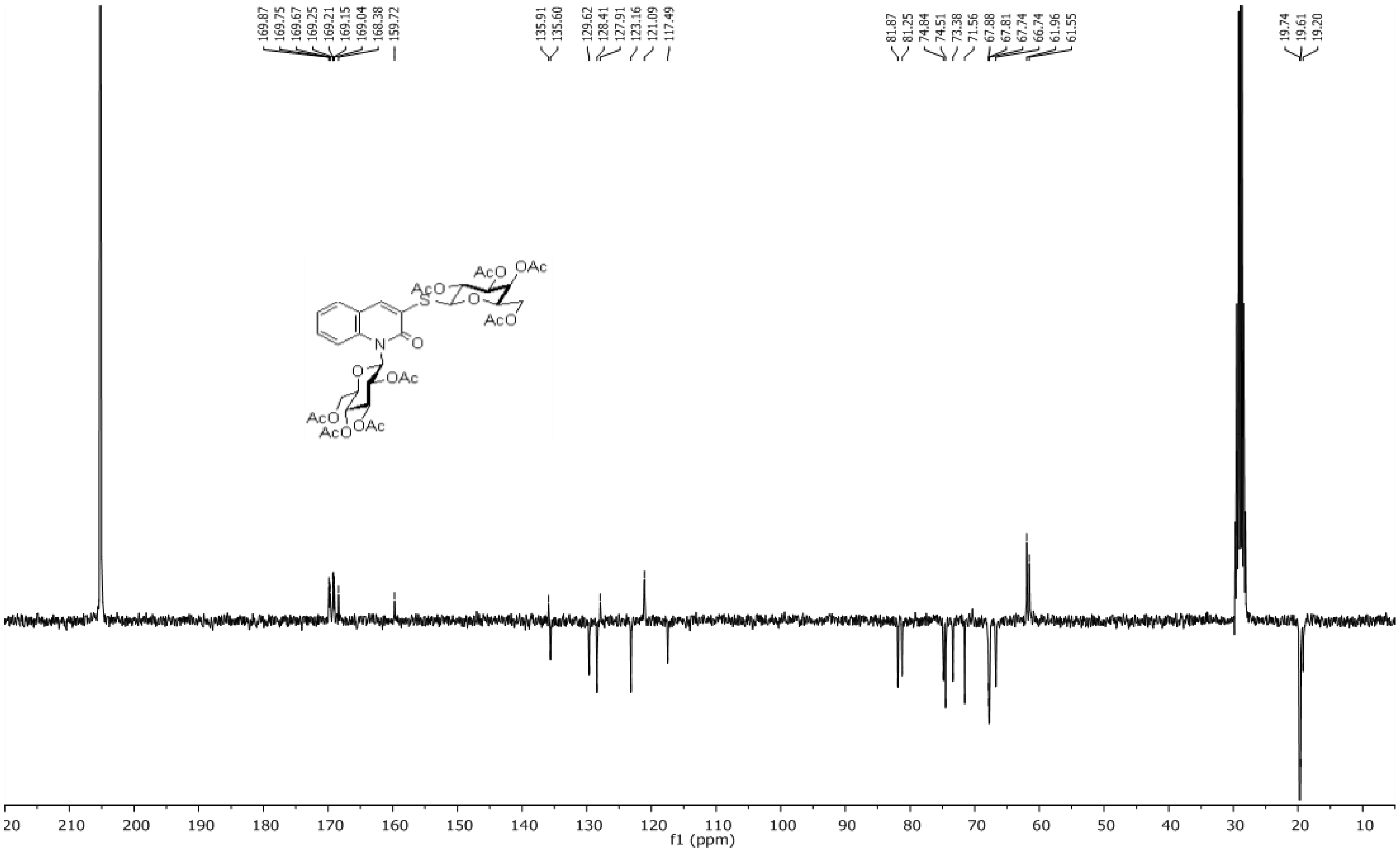

169.87
169.75
169.67
169.25
169.21
169.15
169.04
168.38
159.72
135.91
135.60
129.62
128.41
127.91
123.16
121.09
117.49
81.87
81.25
74.84
74.51
73.38
71.56
67.88
67.81
67.74
66.74
61.96
61.55
19.74
19.61
19.20
AcO
OAc
S
O
N
OAc
f1 (ppm)

***(2R,3R,4S,5R,6S)-2-(acetoxymethyl)-6-((2-oxo-1-((2R,3R,5R,6R)-3,4,5-triacetoxy-6-(acetoxymethyl)tetrahydro-2H-pyran-2-yl)-1,2-dihydroquinolin-3-yl)thio)tetrahydro-2H-pyran-3,4,5-triyl triacetate* 3b (in Acetone, 300 MHz):**

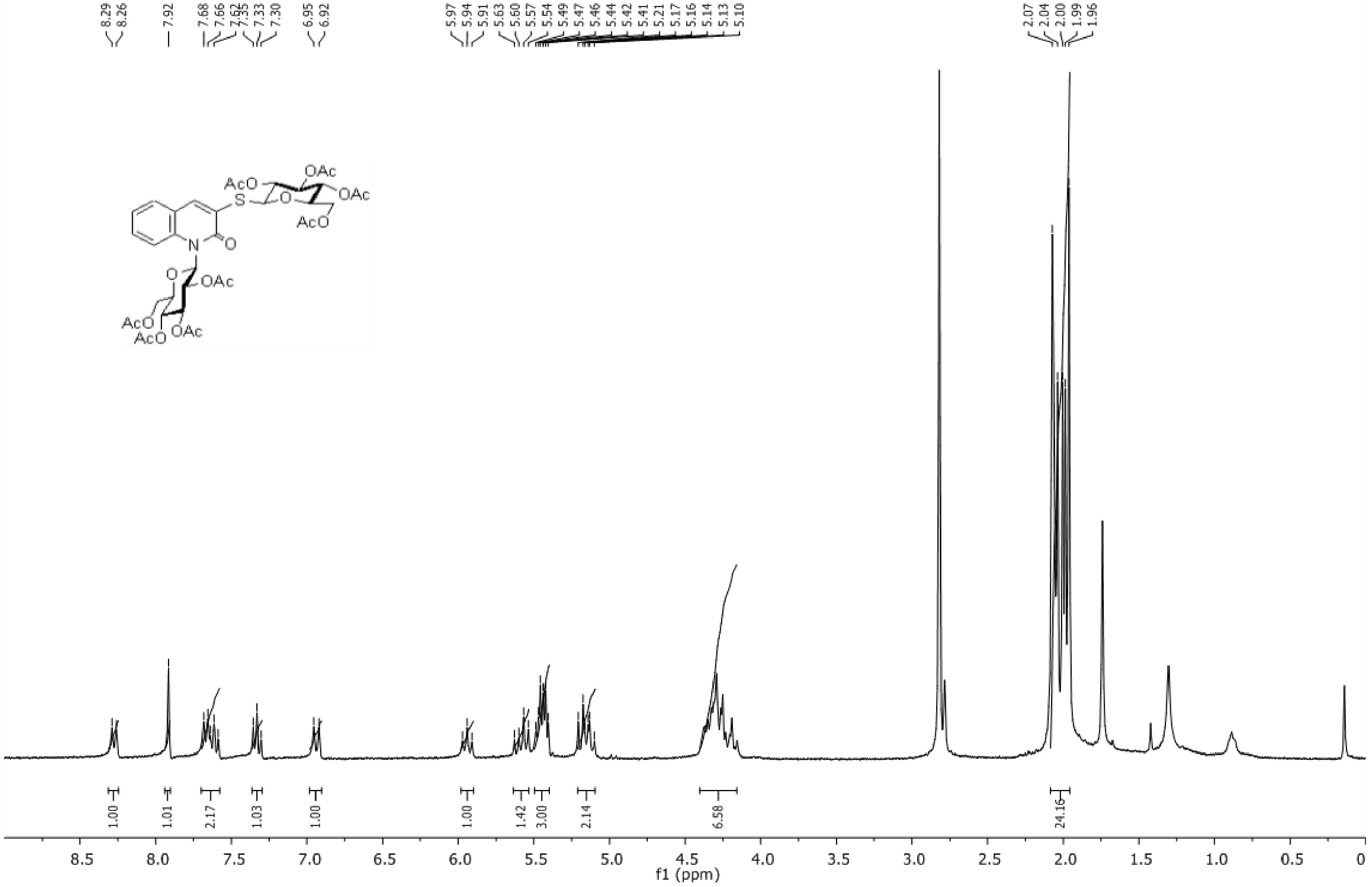

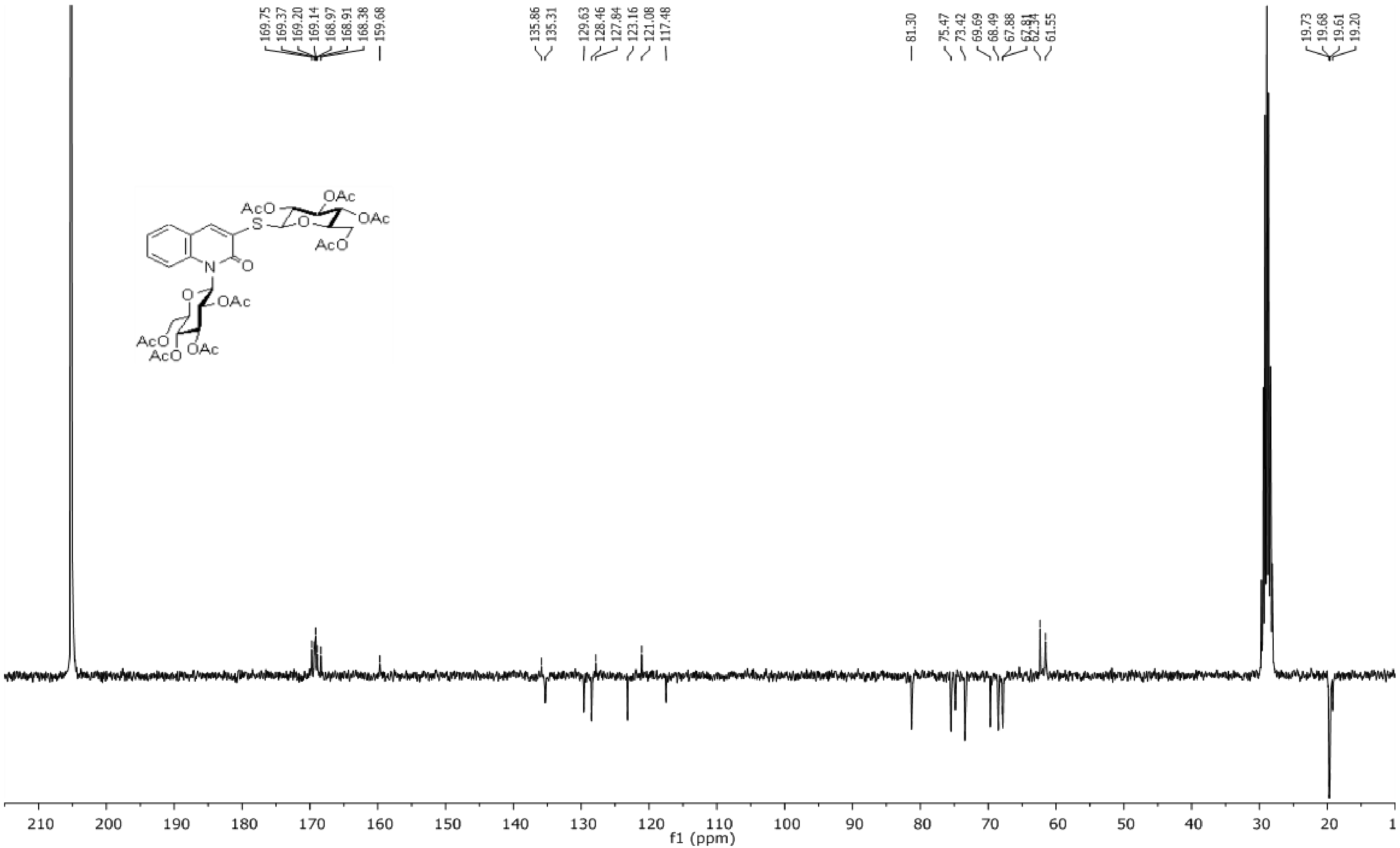
169.75
169.37
169.20
169.14
168.97
168.91
168.38
159.68
135.86
135.31
129.63
128.46
127.84
123.16
121.08
117.48
81.30
75.47
73.42
69.69
68.49
67.88
62.34
61.55
19.73
19.68
19.61
19.20
AcO
OAc
OAc
AcO
S
O
N
O
OAc
AcO
AcO
OAc
210
200
190
180
170
160
150
140
130
120
110
100
90
80
70
60
50
40
30
20
f1 (ppm)

2R,3R,4S,5R,6S)-2-((benzoyloxy)methyl)-6-((2-oxo-1-((2R,3R,5R,6R)-3,4,5-triacetoxy-6-(acetoxymethyl)tetrahydro-2H-pyran-2-yl)-1,2-dihydroquinolin-3-yl)thio)tetrahydro-2H-pyran-3,4,5-triyl tribenzoate **3c (in Acetone, 300 MHz):**

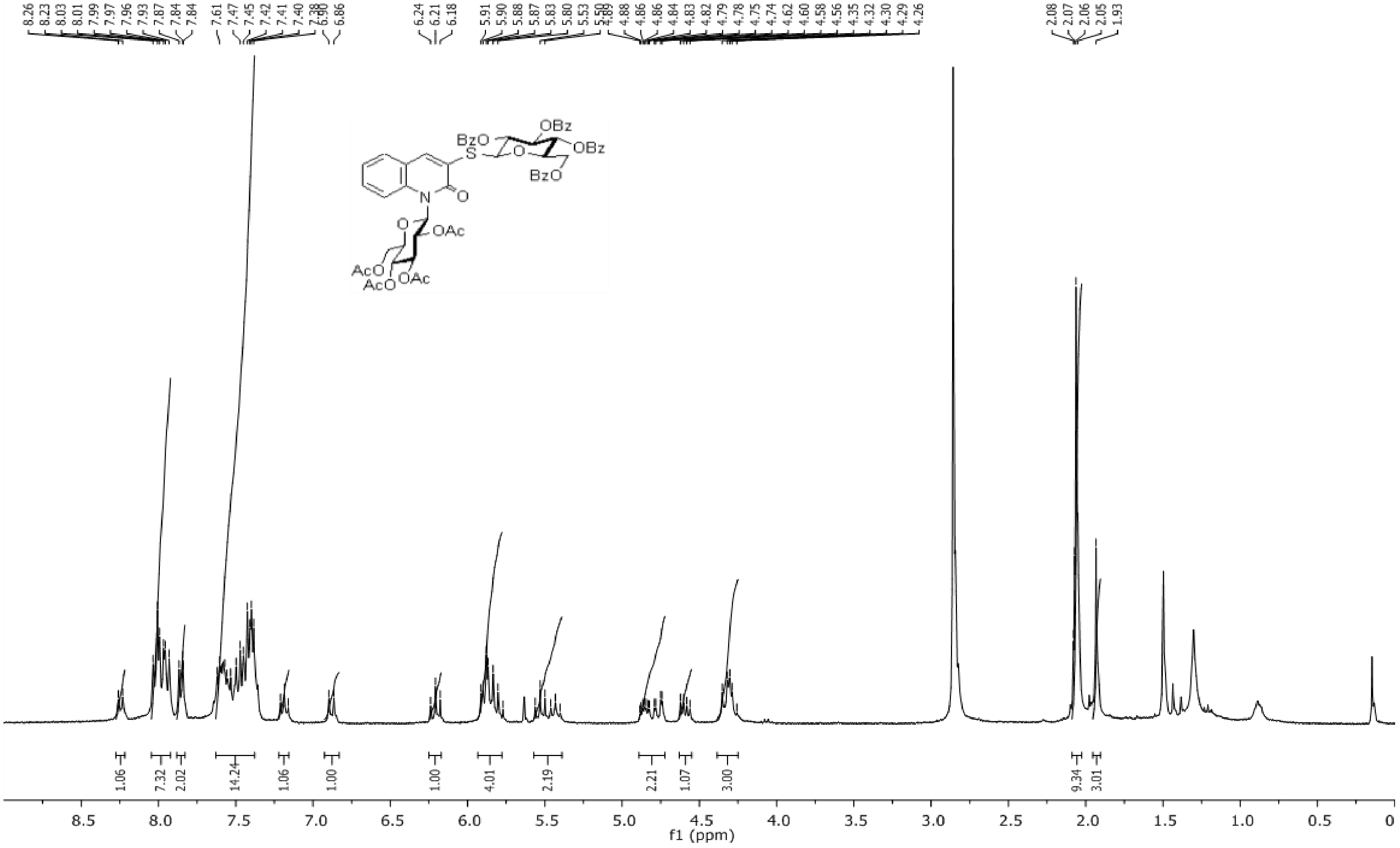

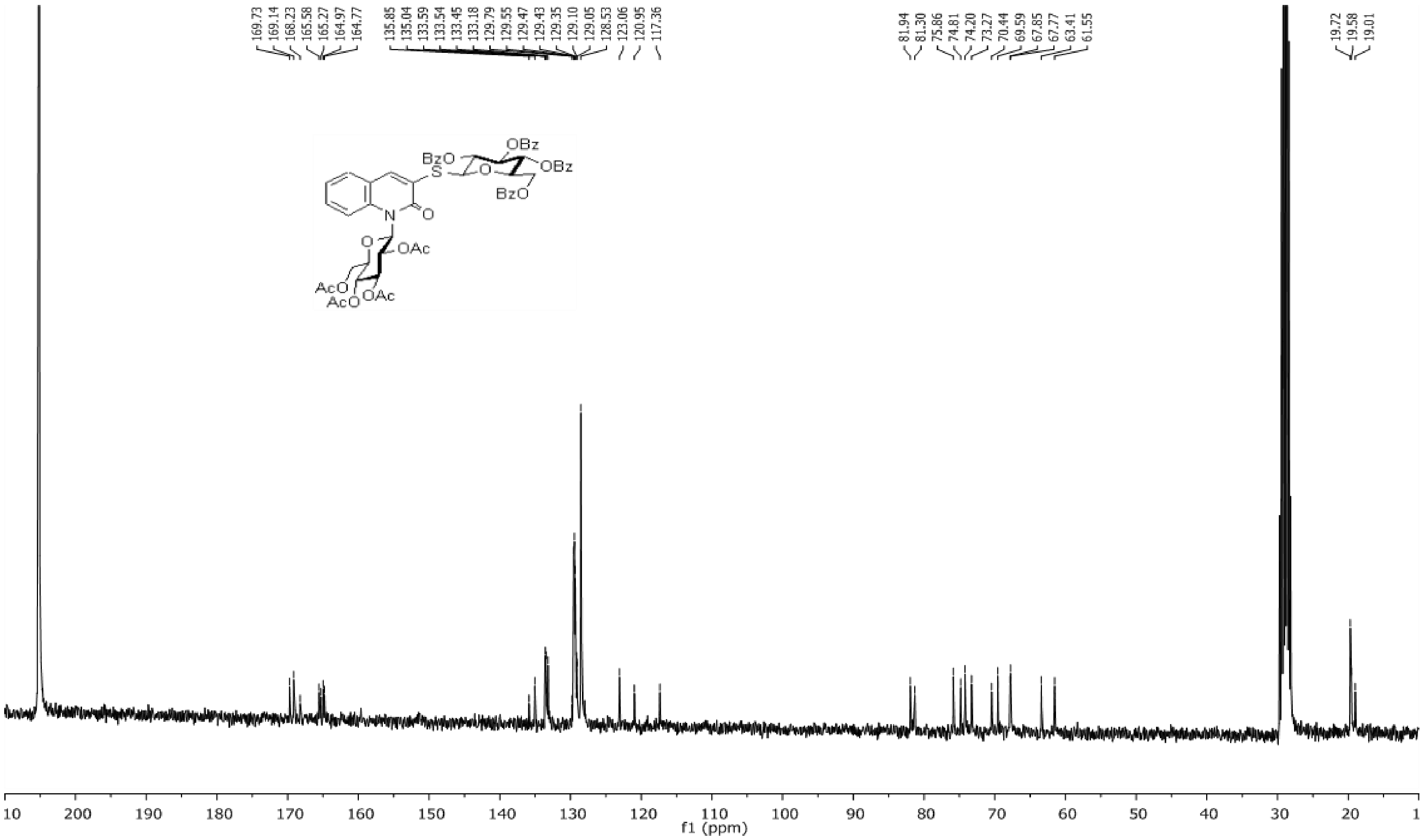
169.73
169.14
168.23
165.58
165.27
164.97
164.77
135.85
135.04
133.59
133.54
133.45
133.18
129.79
129.55
129.47
129.43
129.35
129.10
129.05
128.53
123.06
120.95
117.36
81.94
81.30
75.86
74.81
74.20
73.27
70.44
69.59
67.85
67.77
63.41
61.55
19.72
19.58
19.01
OBz
BzO
S
O
OBz
BzO
N
O
O
OAc
AcO
AcO
OAc
10 200 190 180 170 160 150 140 130 120 110 100 90 80 70 60 50 40 30 20 1
f1 (ppm)

(2R,3R,5R,6R)-2-(3-(((2S,3R,4R,5S,6R)-3-acetamido-4,5-diacetoxy-6-(acetoxymethyl)tetrahydro-2H-pyran-2-yl)thio)-2-oxoquinolin-1(2H)-yl)-6-(acetoxymethyl)tetrahydro-2H-pyran-3,4,5-triyl triacetate **3d** **(in** $CDCl_3$**, 300 MHz):**

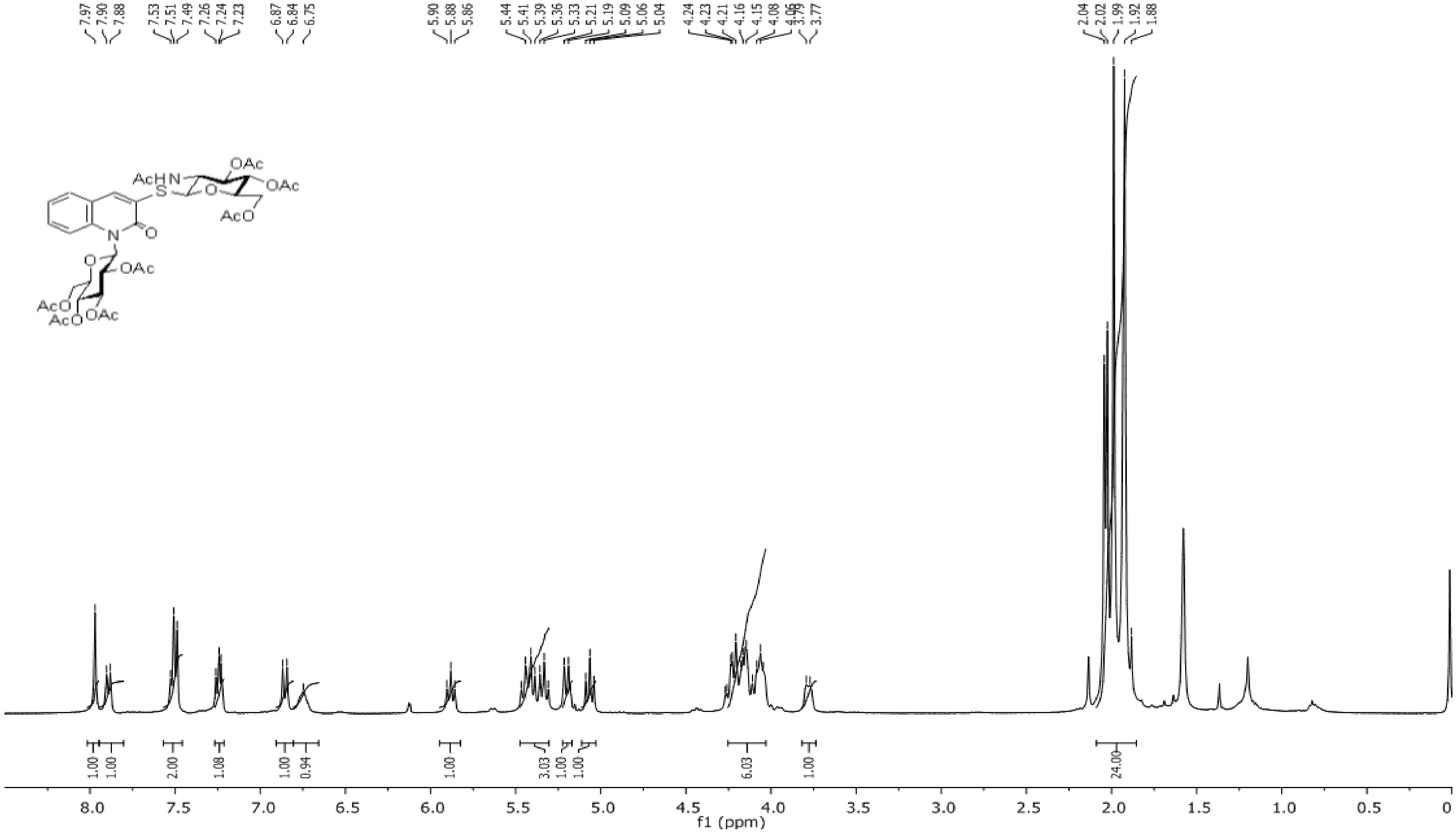

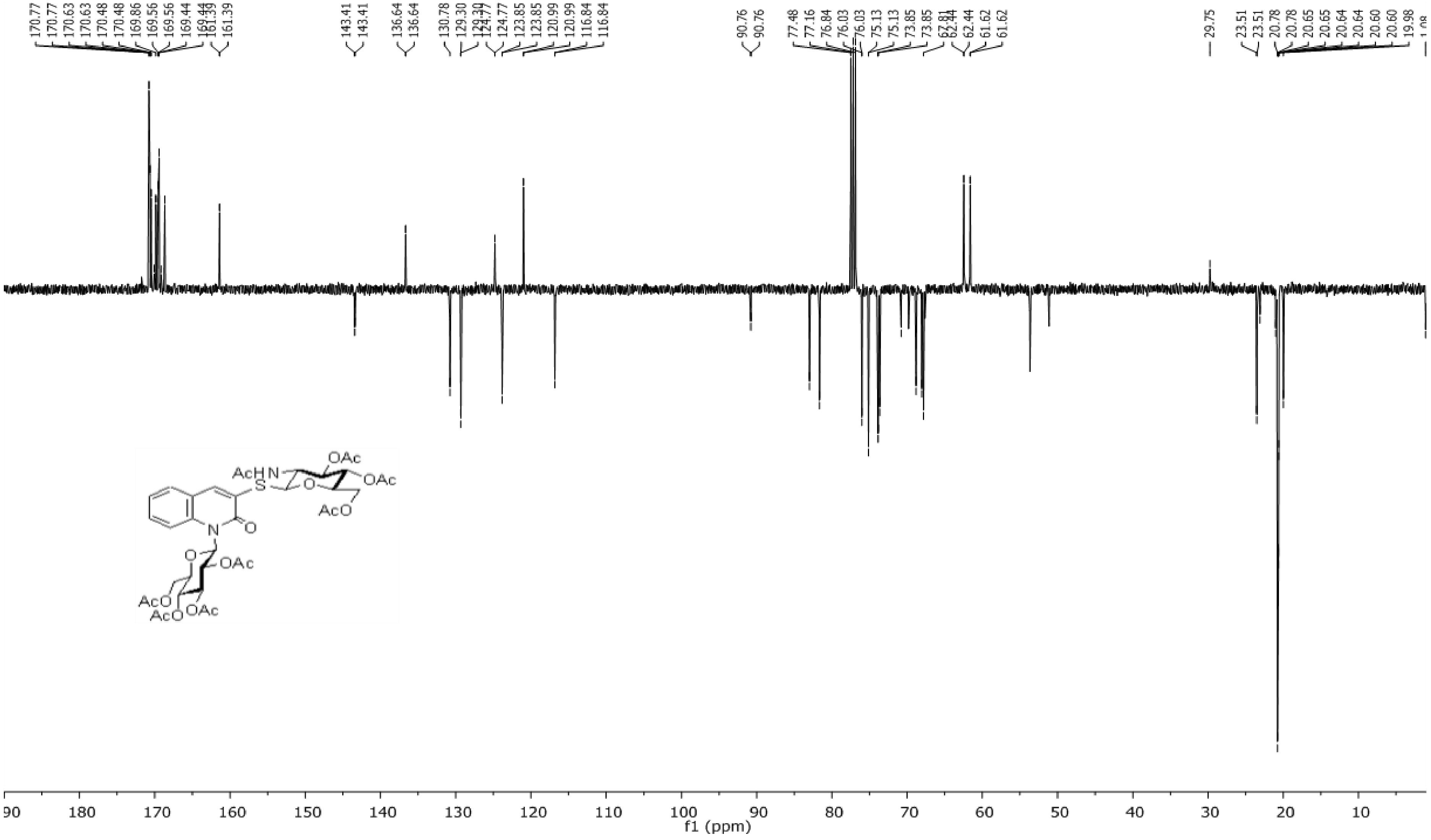
170.77
170.77
170.63
170.63
170.48
170.48
169.86
169.56
169.56
169.44
161.39
161.39
143.41
143.41
136.64
136.64
130.78
129.30
124.77
124.77
123.85
123.85
120.99
120.99
116.84
116.84
90.76
90.76
77.48
77.16
76.84
76.03
76.03
75.13
75.13
73.85
73.85
62.44
62.44
61.62
61.62
29.75
23.51
23.51
20.78
20.78
20.65
20.65
20.64
20.64
20.60
20.60
19.98
AcHN
OAc
S
O
OAc
AcO
N
O
O
OAc
AcO
AcO
OAc
90
180
170
160
150
140
130
120
110
100
90
80
70
60
50
40
30
20
10
f1 (ppm)

(2R,4S,5R,6S)-2-(acetoxymethyl)-6-((2-oxo-1-((2R,3R,5S,6R)-3,4,5-trihydroxy-6-(hydroxymethyl)tetrahydro-2H-pyran-2-yl)-1,2-dihydroquinolin-3-yl)thio)tetrahydro-2H-pyran-3,4,5-triyl triacetate **3[e] (in Acetone, 300 MHz):**

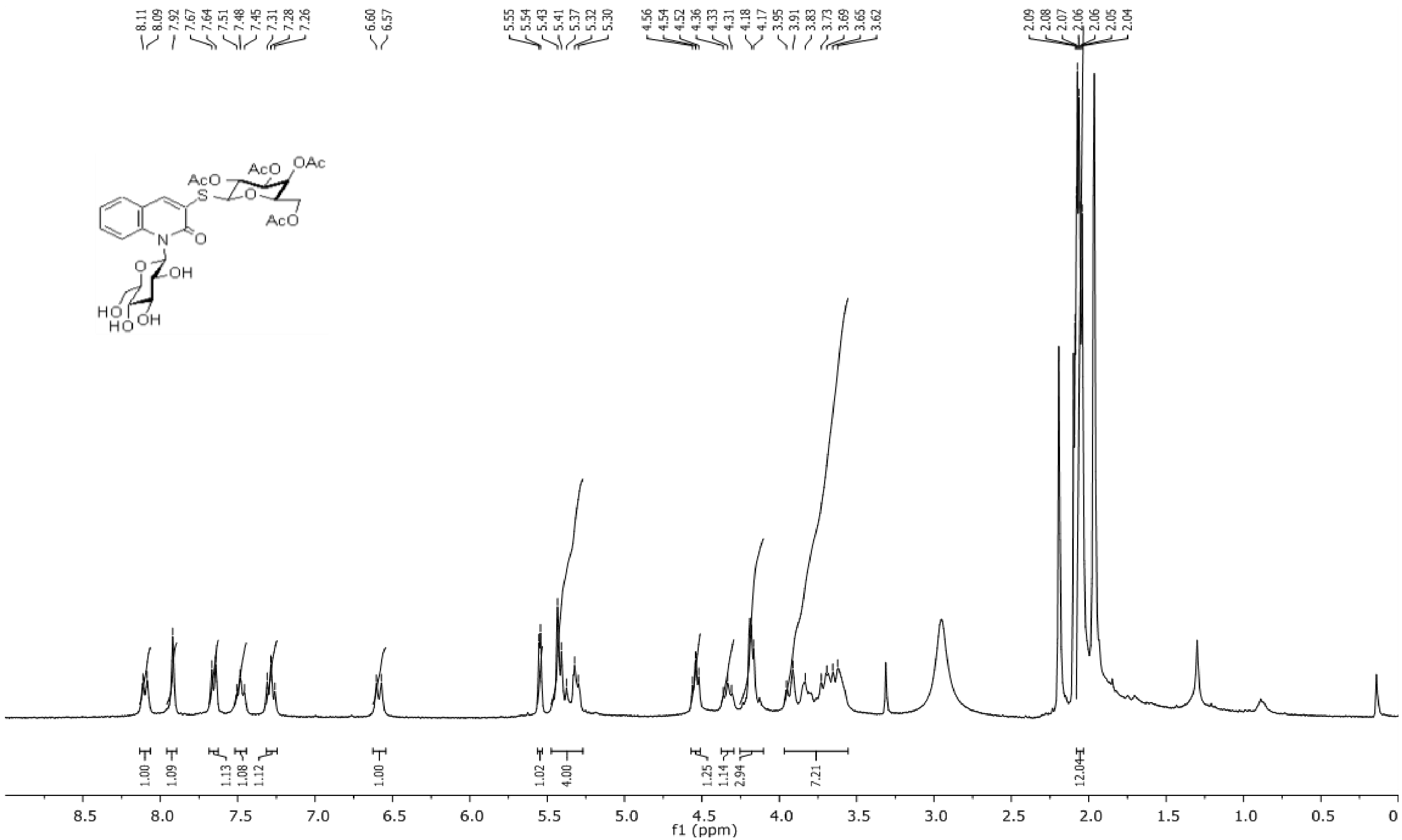

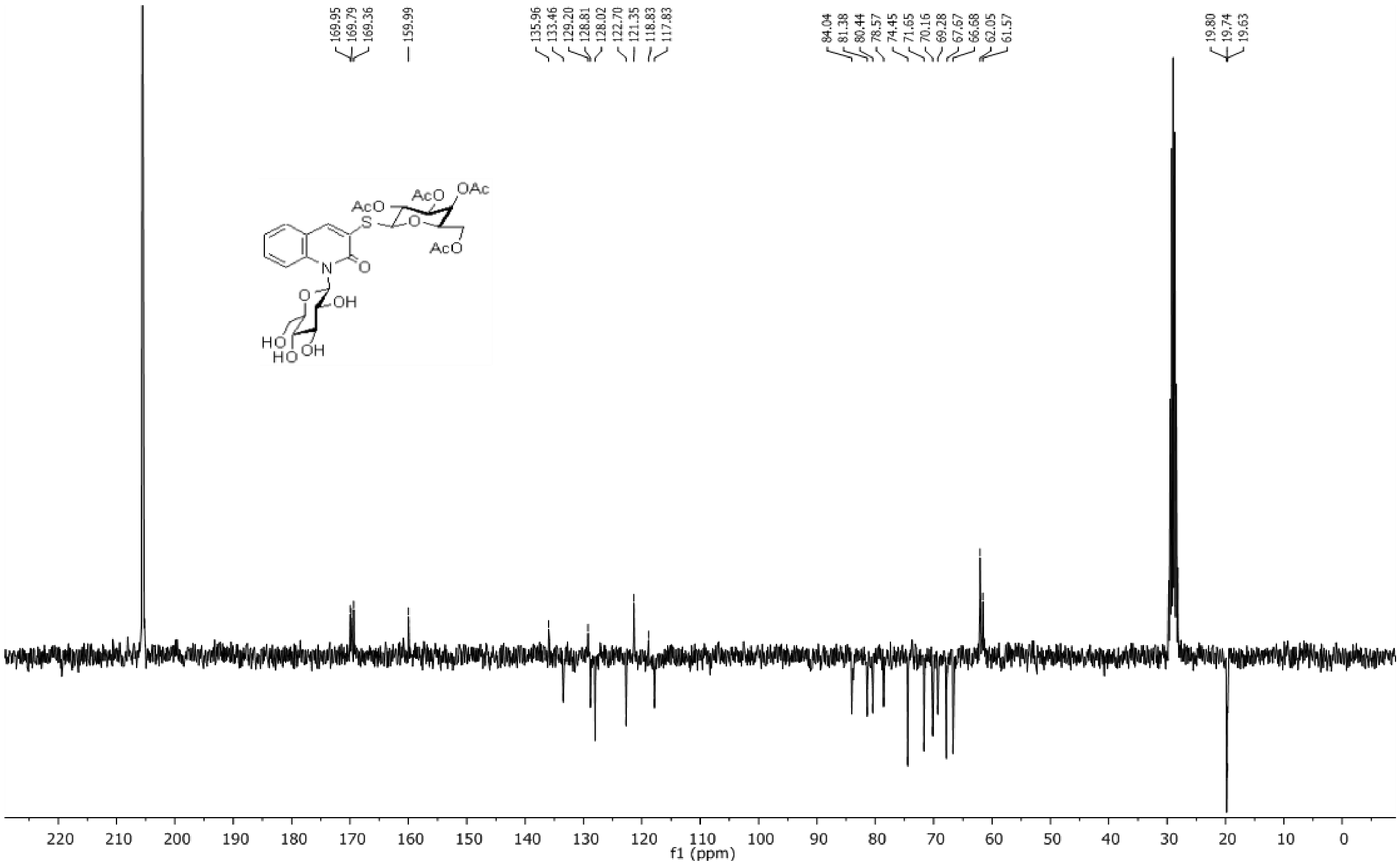
169.95
169.79
169.36
159.99
135.96
133.46
129.20
128.81
128.02
122.70
121.35
118.83
117.83
84.04
81.38
80.44
78.57
74.45
71.65
70.16
69.28
67.67
66.68
62.05
61.57
19.80
19.74
19.63
AcO
OAc
AcO
AcO
S
O
N
O
O
OH
HO
HO
OH
220 210 200 190 180 170 160 150 140 130 120 110 100 90 80 70 60 50 40 30 20 10 0
f1 (ppm)

***(2R,3R,4S,6S)-2-(acetoxymethyl)-6-((4-(4-methoxyphenyl)-2-oxo-1-((2R,3R,5R,6R)-3,4,5-triacetoxy-6-(acetoxymethyl)tetrahydro-2H-pyran-2-yl)-1,2-dihydroquinolin-3-yl)thio)tetrahydro-2H-pyran-3,4,5-triyl triacetate* 3f (in Acetone, 300 MHz):**

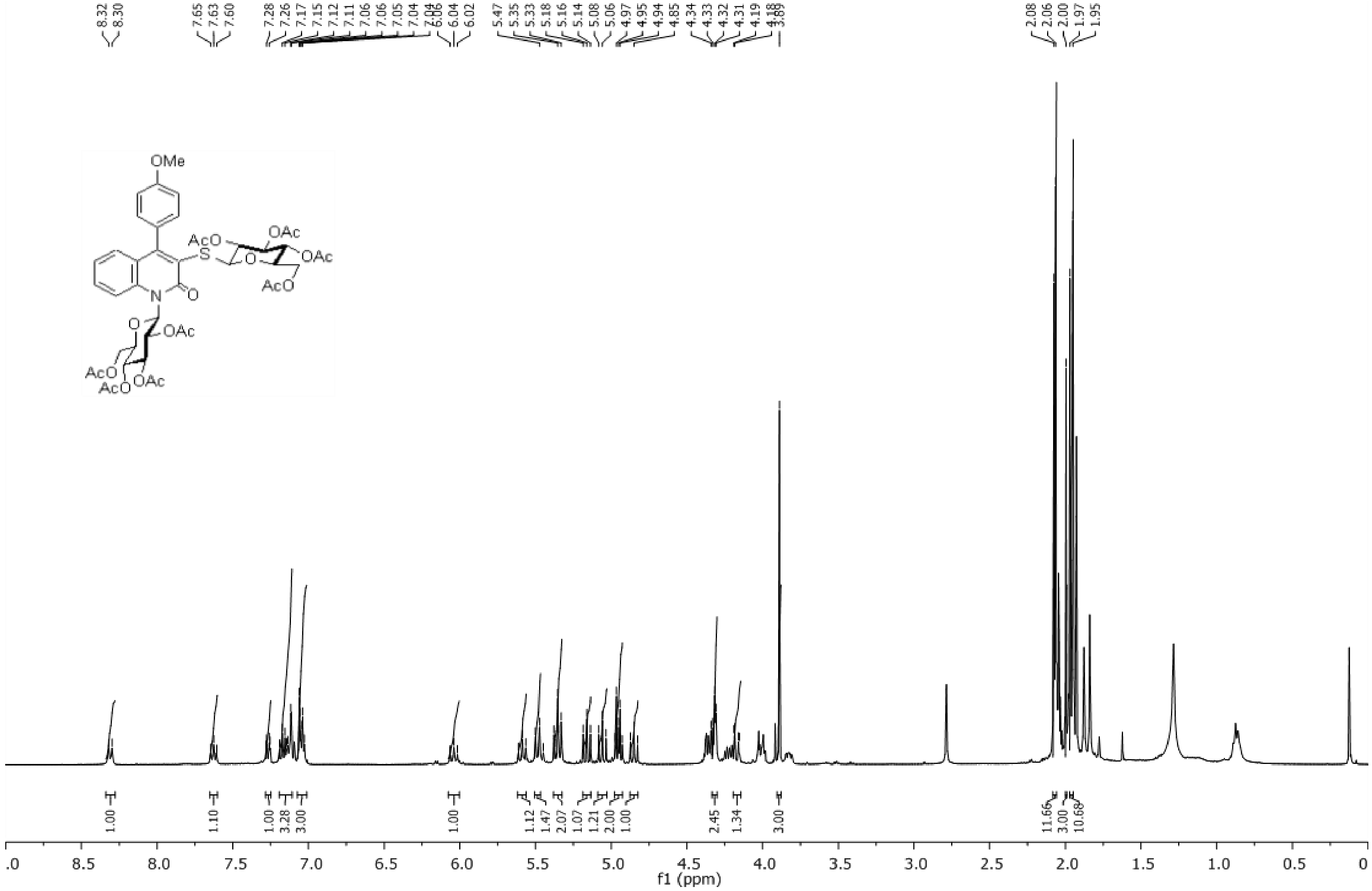

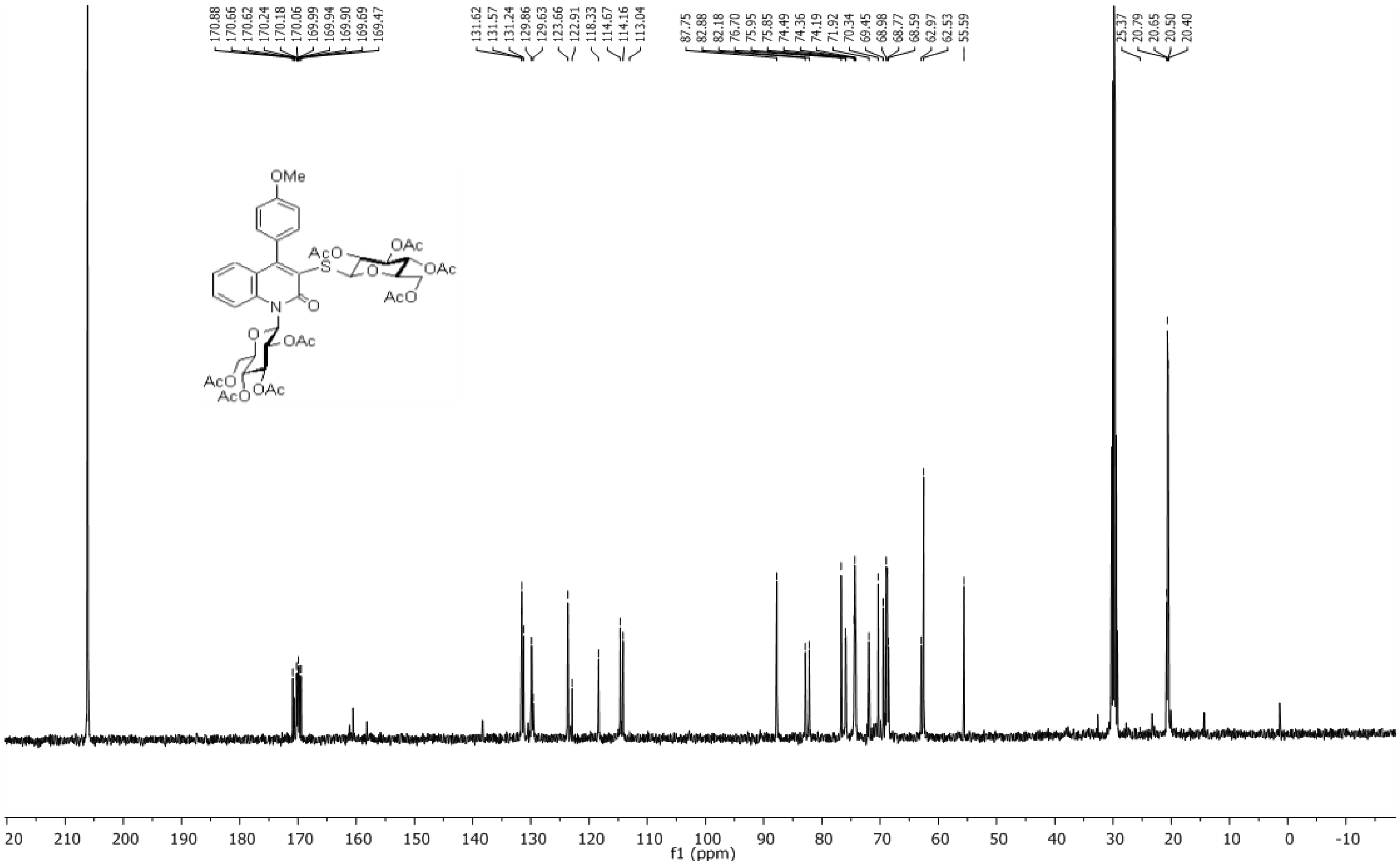
170.88
170.66
170.62
170.24
170.18
170.06
169.99
169.94
169.90
169.69
169.47
131.62
131.57
131.24
129.86
129.63
123.66
122.91
118.33
114.67
114.16
113.04
87.75
82.88
82.18
76.70
75.95
75.85
74.49
74.36
74.19
71.92
70.34
69.45
68.98
68.77
68.59
62.97
62.53
55.59
25.37
20.79
20.65
20.50
20.40
OMe
AcO
S
OAc
OAc
AcO
N
O
OAc
AcO
AcO
OAc
f1 (ppm)

(2R,3R,4S,5R,6S)-2-(acetoxymethyl)-6-(((2R,3R,4S,5R,6S)-4,5-diacetoxy-2-(acetoxymethyl)-6-((2-oxo-1-((2R,3R,5R,6R)-3,4,5-triacetoxy-6-(acetoxymethyl)tetrahydro-2H-pyran-2-yl)-1,2-dihydroquinolin-3-yl)thio)tetrahydro-2H-pyran-3-yl)oxy)tetrahydro-2H-pyran-3,4,5-triyl triacetate **3g (in** $CDCl_3$**, 300 MHz):**

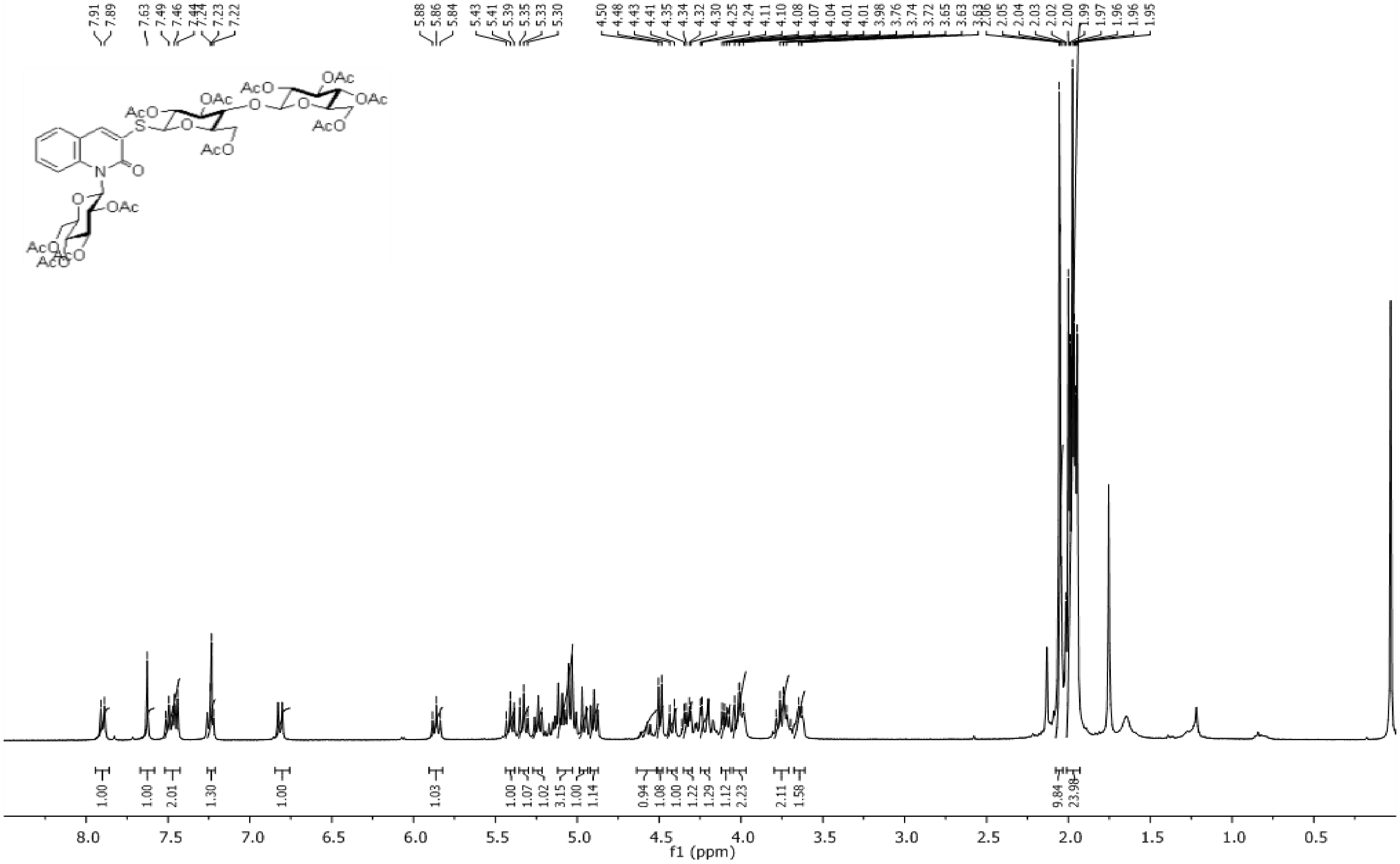

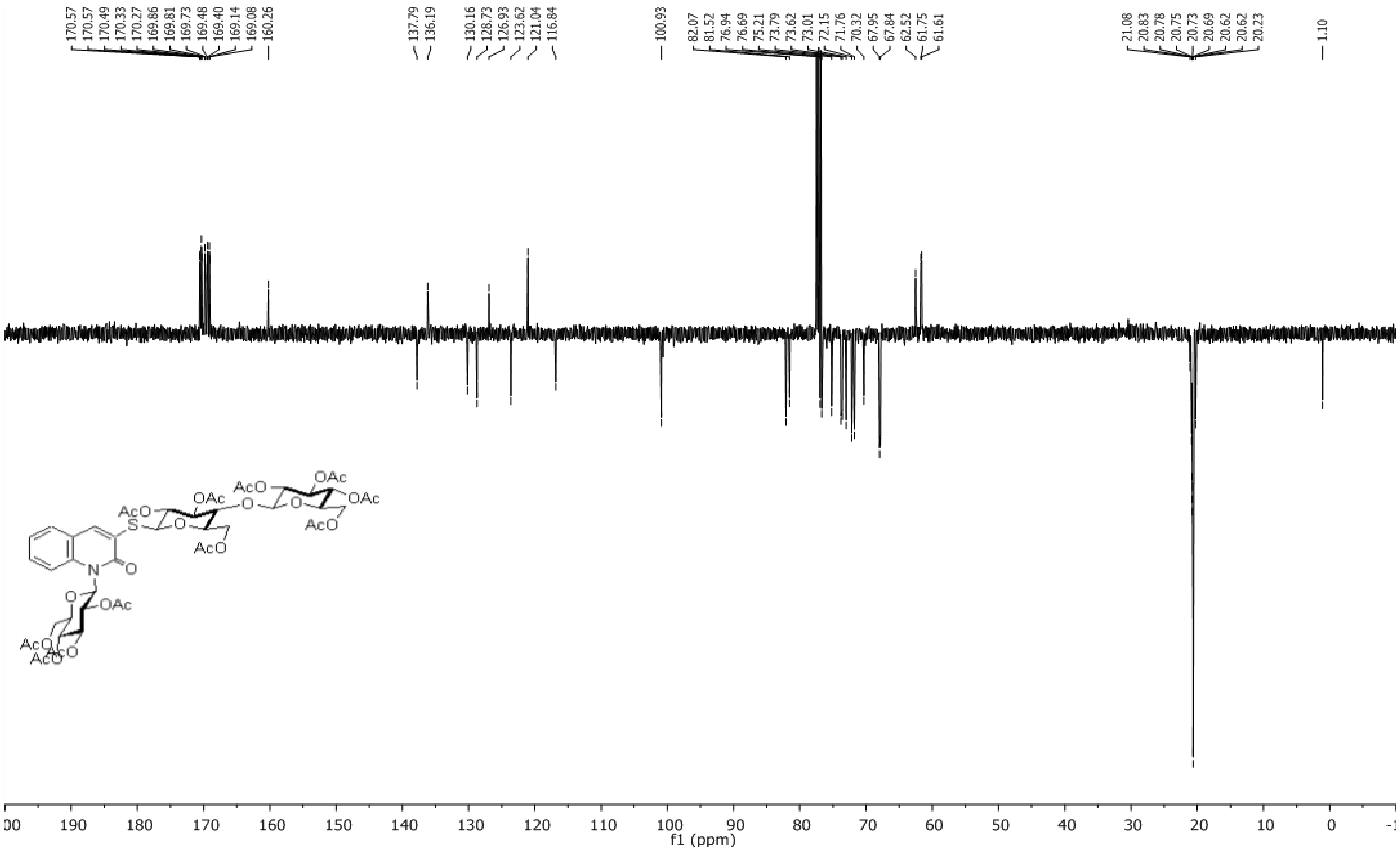
170.57
170.57
170.49
170.33
170.27
169.86
169.81
169.73
169.48
169.40
169.14
169.08
160.26
137.79
136.19
130.16
128.73
126.93
123.62
121.04
116.84
100.93
82.07
81.52
76.94
76.69
75.21
73.79
73.62
73.01
72.15
71.76
70.32
67.95
67.84
62.52
61.75
61.61
21.08
20.83
20.78
20.75
20.73
20.69
20.62
20.62
20.23
1.10
f1 (ppm)

***(2R,3R,4S,5R,6S)-2-(acetoxymethyl)-6-(((2R,3R,4S,5R,6S)-4,5-diacetoxy-2-(acetoxymethyl)-6-((2-oxo-1-((2R,3R,5S,6R)-3,4,5-trihydroxy-6-(hydroxymethyl)tetrahydro-2H-pyran-2-yl)-1,2-dihydroquinolin-3-yl)thio)tetrahydro-2H-pyran-3-yl)oxy)tetrahydro-2H-pyran-3,4,5-triyl triacetate* 3h (in Acetone, 300 MHz):**

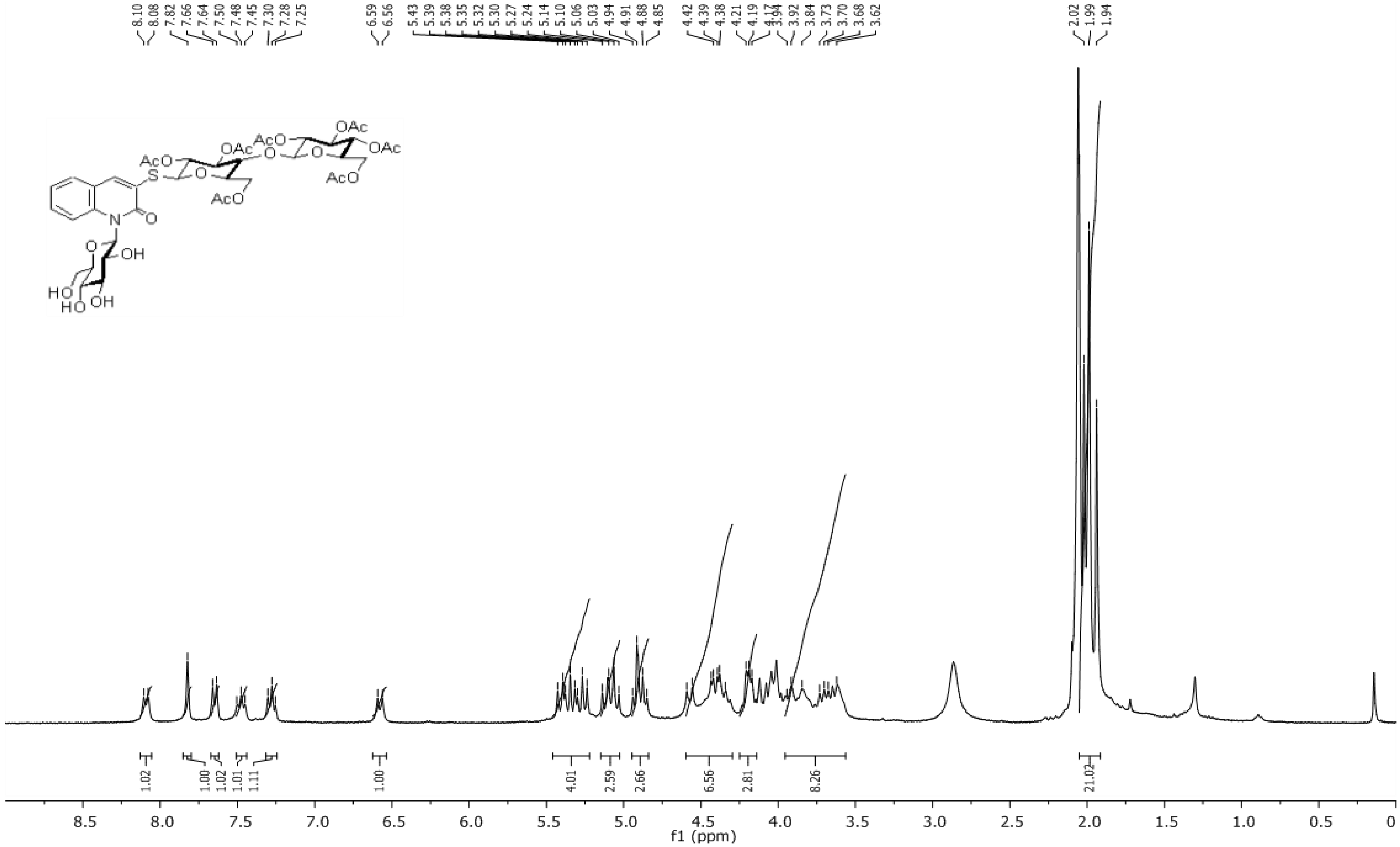

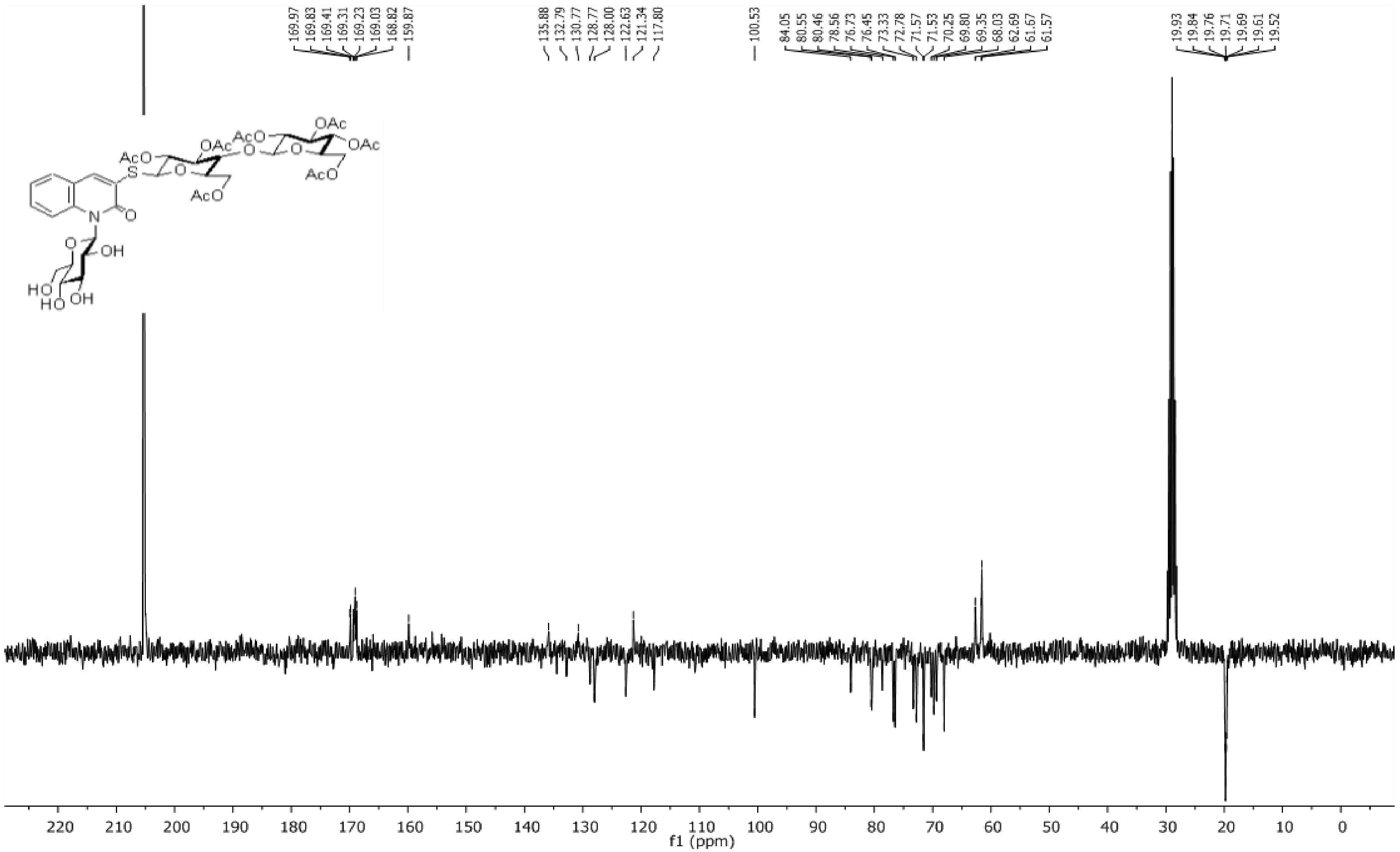
169.97
169.83
169.41
169.31
169.23
169.03
168.82
159.87
135.88
132.79
130.77
128.77
128.00
122.63
121.34
117.80
100.53
84.05
80.55
80.46
78.56
76.73
76.45
73.33
72.78
71.57
71.53
70.25
69.80
69.35
68.03
62.69
61.67
61.57
19.93
19.84
19.76
19.71
19.69
19.61
19.52
f1 (ppm)

(2R,3R,4S,5R,6R)-2-(acetoxymethyl)-6-(((2R,3S,4S,5R,6R)-4,5-diacetoxy-2-(acetoxymethyl)-6-(((2R,3S,4S,5R,6S)-4,5-diacetoxy-2-(acetoxymethyl)-6-((2-oxo-1-((2R,3R,5R,6R)-3,4,5-triacetoxy-6-(acetoxymethyl)tetrahydro-2H-pyran-2-yl)-1,2-dihydroquinolin-3-yl)thio)tetrahydro-2H-pyran-3-yl)oxy)tetrahydro-2H-pyran-3-yl)oxy)tetrahydro-2H-pyran-3,4,5-triyl triacetate **3i** **(in** CDCl$_3$, **300 MHz):**

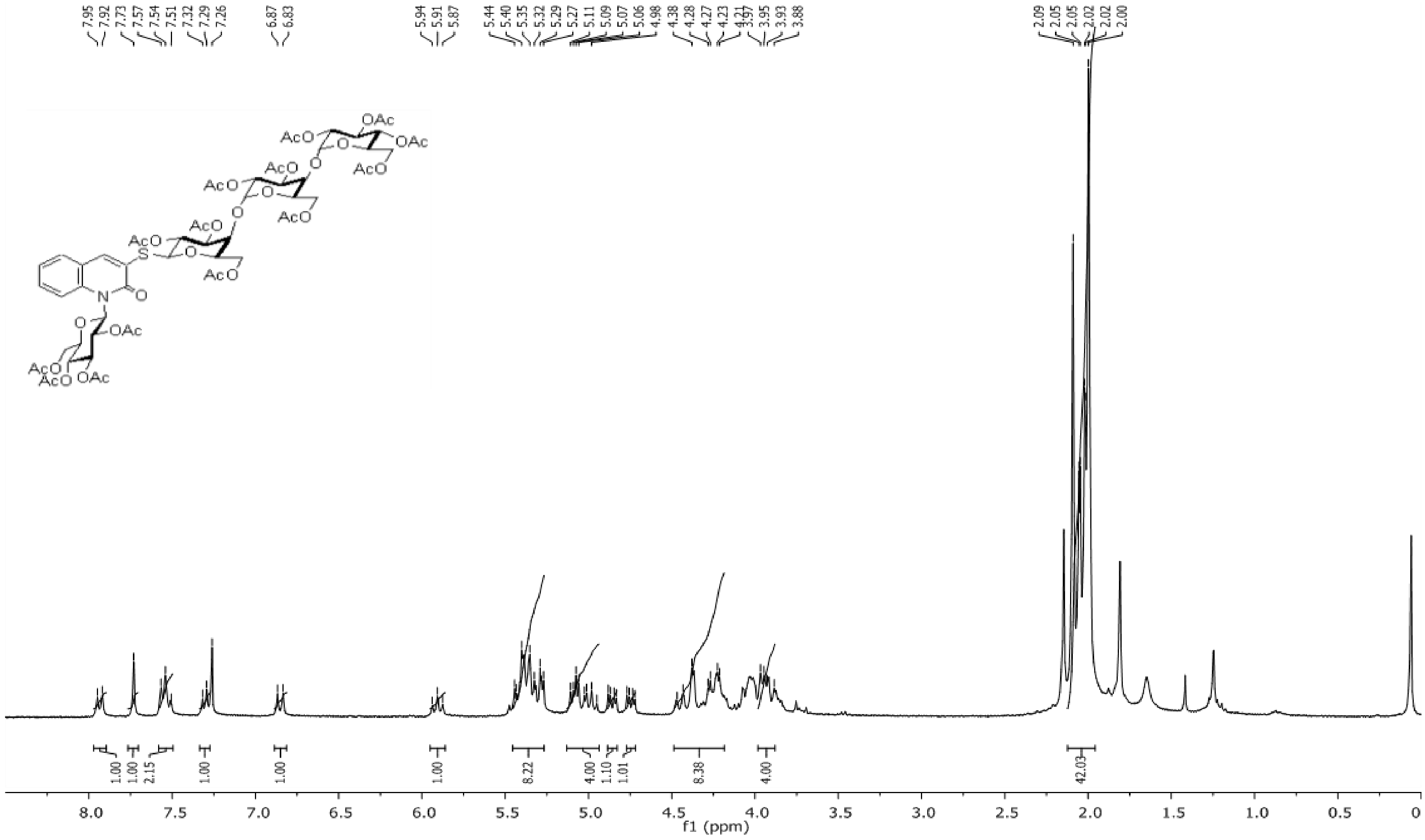

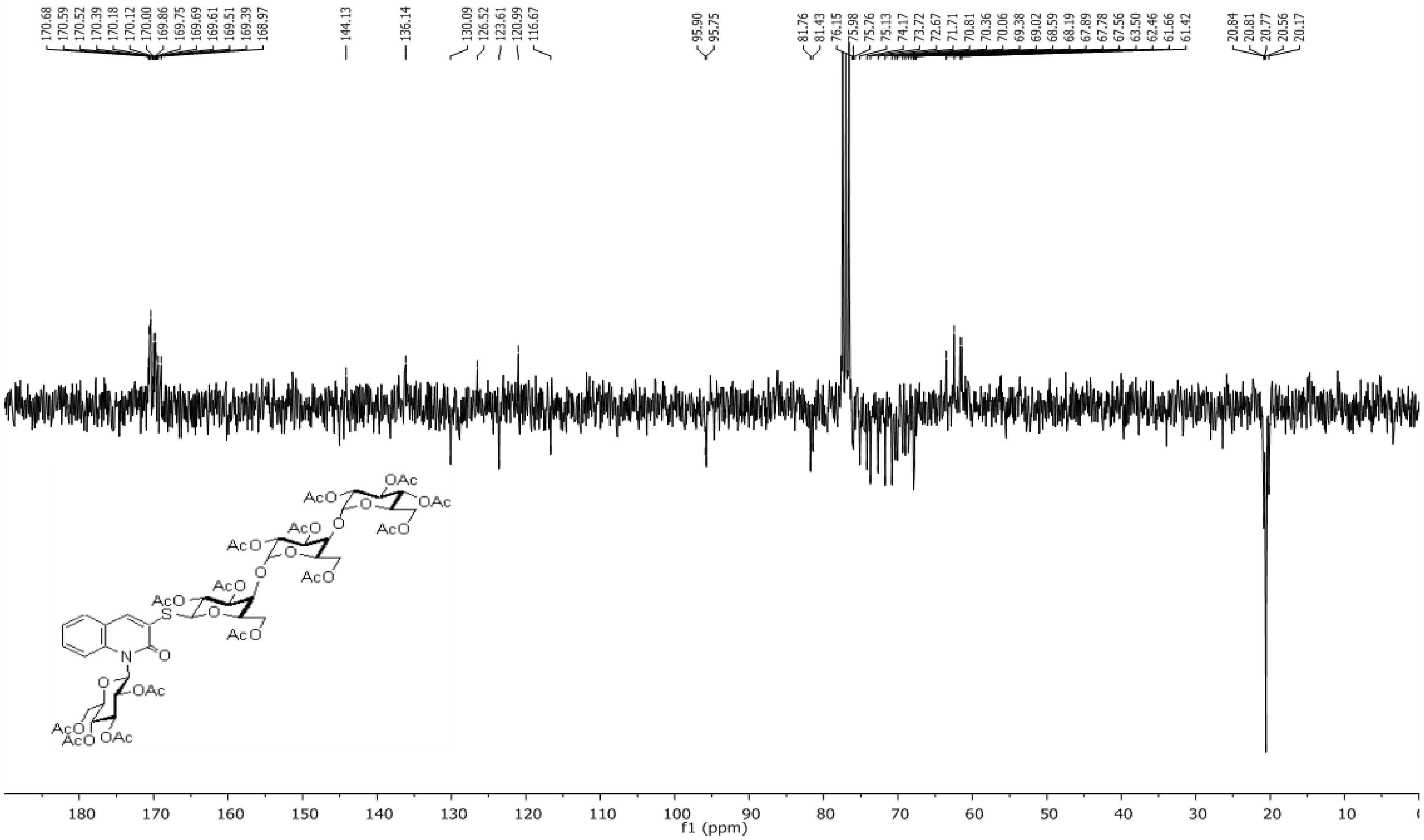
170.68
170.59
170.52
170.39
170.18
170.12
170.00
169.86
169.75
169.69
169.61
169.51
169.39
168.97
144.13
136.14
130.09
126.52
123.61
120.99
116.67
95.90
95.75
81.76
81.43
76.15
75.98
75.76
75.13
74.17
73.72
72.67
71.71
70.81
70.36
70.06
69.38
69.02
68.59
68.19
67.89
67.78
67.56
63.50
62.46
61.66
61.42
20.84
20.81
20.77
20.56
20.17
AcO
OAc
S
N
O
f1 (ppm)

2R,3R,4S,5R,6S)-2-(acetoxymethyl)-6-((2-oxo-1-((2R,3S,4R,5S,6S)-3,4,5-triacetoxy-6-(acetoxymethyl)tetrahydro-2H-pyran-2-yl)-1,2-dihydroquinolin-3-yl)thio)tetrahydro-2H-pyran-3,4,5-triyl triacetate **3j (in Acetone, 300 MHz):**

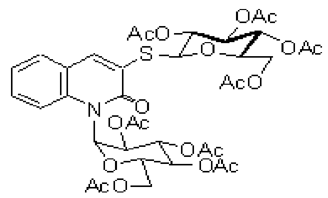

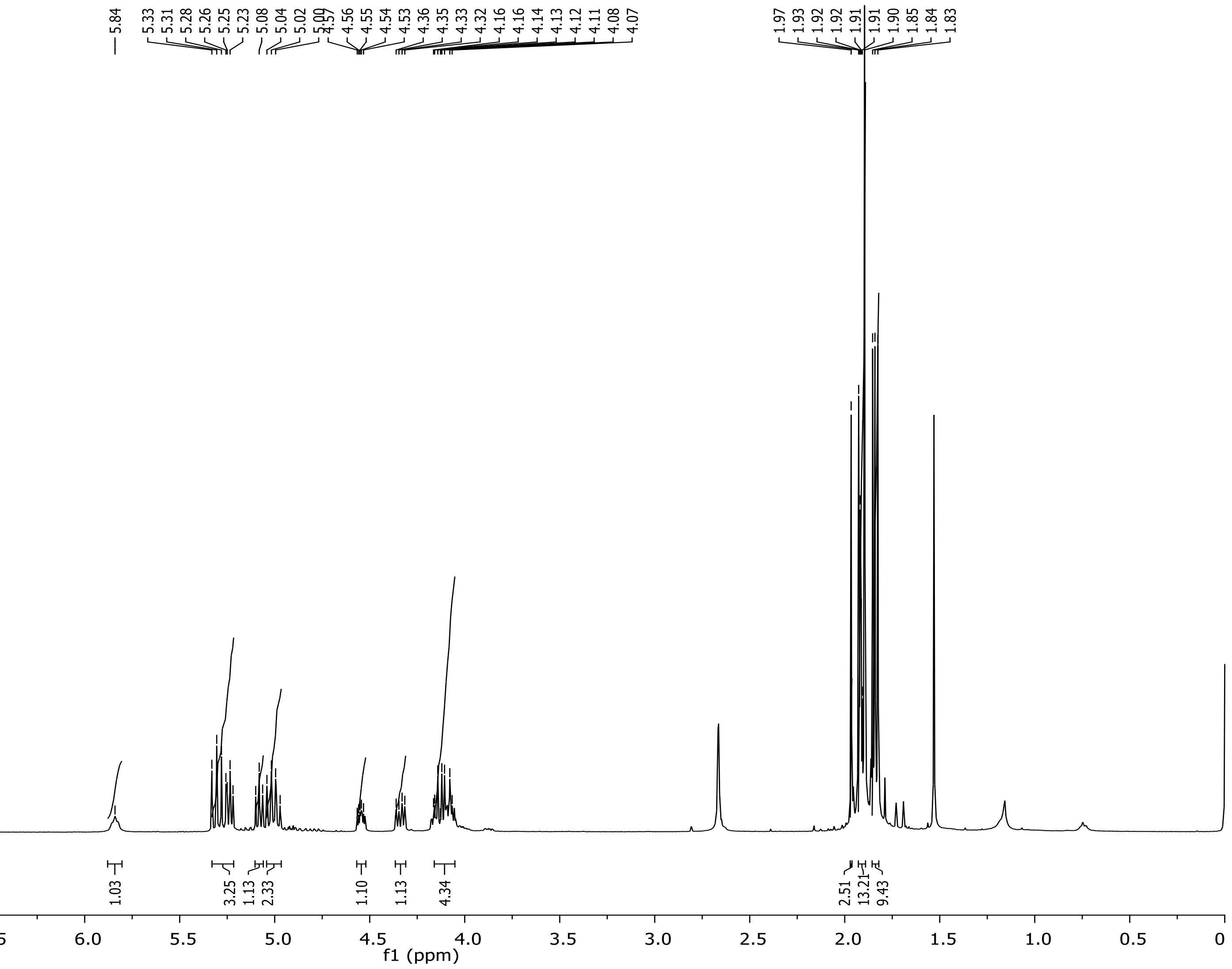

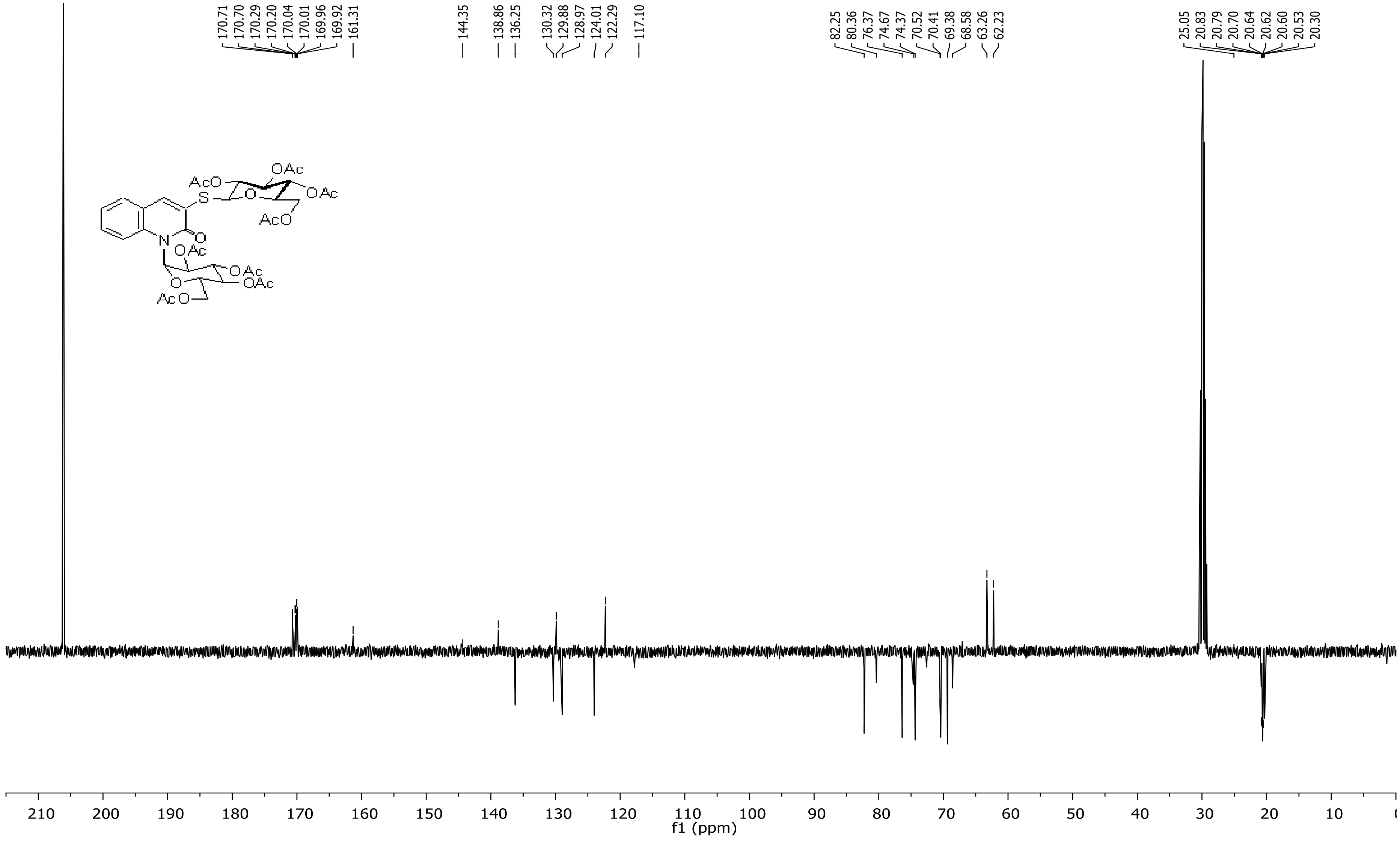
170.71
170.70
170.29
170.20
170.04
170.01
169.96
169.92
161.31
144.35
138.86
136.25
130.32
129.88
128.97
124.01
122.29
117.10
82.25
80.36
76.37
74.67
74.37
70.52
70.41
69.38
68.58
63.26
62.23
25.05
20.83
20.79
20.70
20.64
20.62
20.60
20.53
20.30
AcO
OAc
OAc
S
O
AcO
N
O
OAc
OAc
OAc
O
AcO
210 200 190 180 170 160 150 140 130 120 110 100 90 80 70 60 50 40 30 20 10
f1 (ppm)

1-((2R,3R,5S,6R)-3,4,5-trihydroxy-6-(hydroxymethyl)tetrahydro-2H-pyran-2-yl)-3-(((2S,3R,4S,5S,6R)-3,4,5-trihydroxy-6-(hydroxymethyl)tetrahydro-2H-pyran-2-yl)thio)quinolin-2(1H)-one **(in DMSO-*d6*, 300 MHz): 4a**

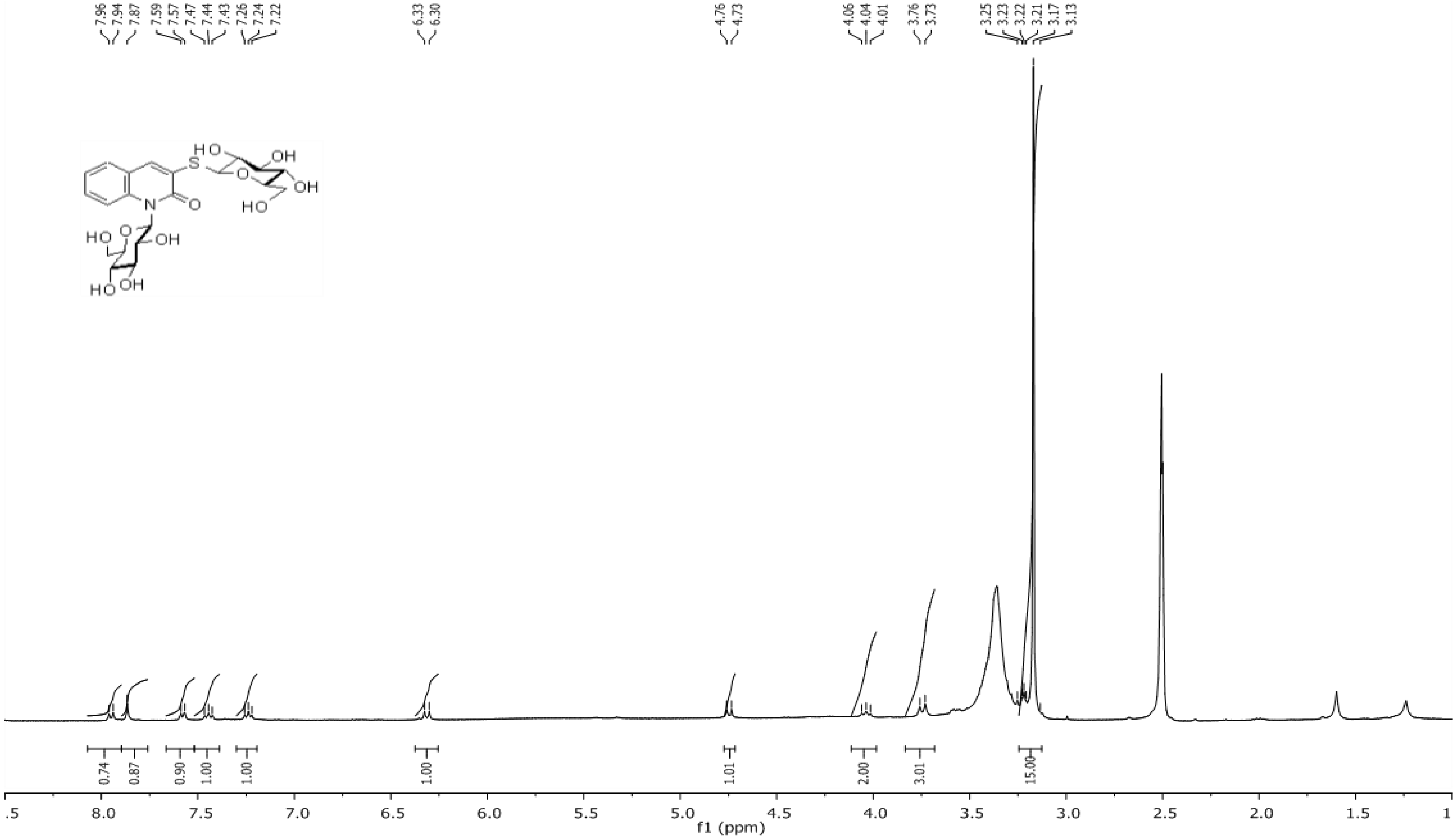

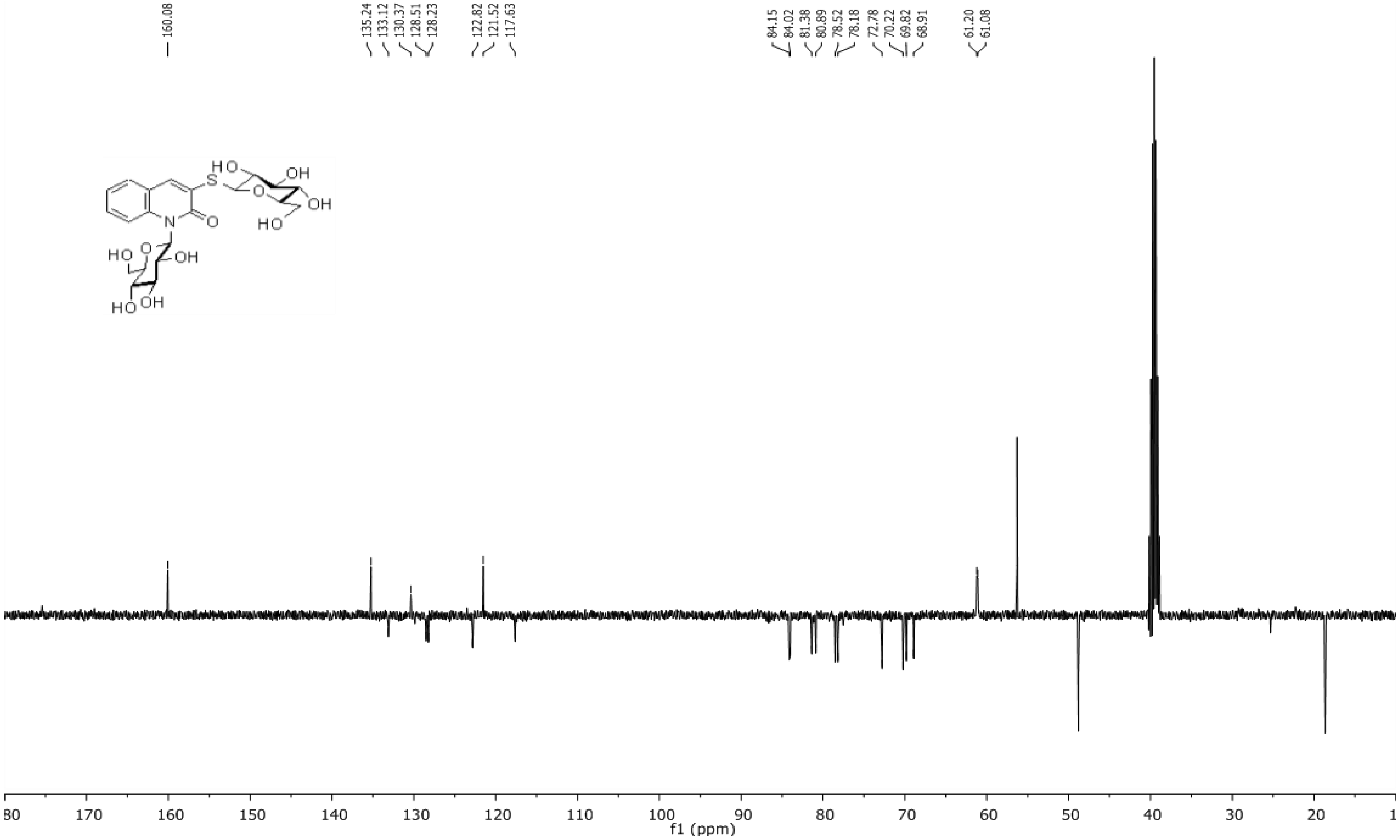
160.08
135.24
133.12
130.37
128.51
128.23
122.82
121.52
117.63
84.15
84.02
81.38
80.89
78.52
78.18
72.78
70.22
69.82
68.91
61.20
61.08
170
160
150
140
130
120
110
100
90
80
70
60
50
40
30
20
f1 (ppm)
